# Global variation in antibiotic prescribing guidelines and the implications for decreasing AMR in the future

**DOI:** 10.3389/fphar.2025.1600787

**Published:** 2025-08-07

**Authors:** Emmama Jamil, Zikria Saleem, Brian Godman, Matti Ullah, Afreenish Amir, Abdul Haseeb, Johanna C. Meyer, Muhammad Usman Qamar, Safa S. Almarzoky Abuhussain

**Affiliations:** ^1^ Department of Pharmacy Practice, Faculty of Pharmacy, Bahauddin Zakariya University, Multan, Pakistan; ^2^ Department of Pharmacy Practice, Faculty of Pharmacy, Hamdard University Islamabad Campus, Islamabad, Pakistan; ^3^ Department of Pharmacy Practice, College of Pharmacy, Qassim University, Qassim, Saudi Arabia; ^4^ Strathclyde Institute of Pharmacy and Biomedical Sciences, University of Strathclyde, Glasgow, United Kingdom; ^5^ Department of Public Health Pharmacy and Management, School of Pharmacy, Sefako Makgatho Health Sciences University, Ga-Rankuwa, South Africa; ^6^ Centre for Neonatal and Paediatric Infection, Institute for Infection and Immunity, City St. George’s, University of London, London, United Kingdom; ^7^ National Institute of Health (NIH), Islamabad, Pakistan; ^8^ Clinical Pharmacy Department, Al Rayan National College of Health Sciences and Nursing, Al Madinah Al Munawarrah, Madinah, Saudi Arabia; ^9^ South African Vaccination and Immunisation Centre, Sefako Makgatho Health Sciences University, Ga-Rankuwa, South Africa; ^10^ Institute of Microbiology, Faculty of Life Sciences, Government College University Faisalabad, Faisalabad, Pakistan; ^11^ Department of Pharmaceutical Practices, College of Pharmacy, Umm Al-Qura University, Makkah, Saudi Arabia

**Keywords:** antibiotic prescribing guidelines, antimicrobial resistance, AWaRe classification, GRADE methodology, low- and middle-income countries, patient counselling, World Health Organization regions

## Abstract

**Introduction:**

Antimicrobial resistance (AMR) has become a global burden, with inappropriate antibiotic prescribing being an important contributing factor. Antibiotic prescribing guidelines play an important role in improving the quality of antibiotic use, provided they are evidence-based and regularly updated. As a result, they help reduce AMR, which is a critical challenge in low- and middle-income countries (LMICs). Consequently, the objective of this study was to evaluate local, national, and international antibiotic prescribing guidelines currently available—especially among LMICs—and previous challenges, in light of the recent publication of the WHO AWaRe book, which provides future direction.

**Methodology:**

Google Scholar and PubMed searches were complemented by searching official country websites to identify antibiotic prescribing guidelines, especially those concerning empiric treatment of bacterial infections, for this narrative review. Data were collected on the country of origin, income level, guideline title, year of publication, development methodology, issuing organization, target population, scope, and coverage. In addition, documentation on implementation strategies, compliance, monitoring of outcome measures, and any associated patient education or counseling efforts were reviewed to assess guideline utilization.

**Results/findings:**

A total of 181 guidelines were included, with the majority originating from high-income countries (109, 60.2%), followed by lower-middle-income (40, 22.1%), low-income (18, 9.9%), and upper-middle-income (14, 7.7%) countries. The GRADE methodology was used in only 20.4% of the sourced guidelines, predominantly in high-income countries. Patient education was often underemphasized, particularly in LMICs. The findings highlighted significant disparities in the development, adaptation, and implementation of guidelines across different WHO regions, confirming the previously noted lack of standardization and comprehensiveness in LMICs.

**Conclusion:**

Significant disparities exist in the availability, structure, and methodological rigor of antibiotic prescribing guidelines across countries with different income levels. Advancing the development and implementation of standardized, context-specific guidelines aligned with the WHO AWaRe framework—and supported by equity-focused reforms—can significantly strengthen antimicrobial stewardship and help address the public health challenge of AMR.

## 1 Introduction

Inappropriate antibiotic usage is now a critical global health issue as it is a leading cause of antimicrobial resistance (AMR), which results in the reduced effectiveness of antimicrobials (Godman et al., 2021; Aldarhami, 2023; Baran et al., 2023; Ho et al., 2024). According to current estimates, AMR causes nearly 1.17 million deaths annually, with the number of deaths likely to more than double by 2050 if key issues and challenges are not addressed (Murray et al., 2022; Do et al., 2023). Infections caused by resistant bacteria increase morbidity and treatment costs, along with higher mortality and the risk of such infections spreading (Dadgostar, 2019; Wagenlehner and Dittmar, 2022; Spellberg et al., 2025; Wan et al., 2025). The impact of AMR is likely to be greater in low- and middle-income countries (LMICs), where the infection burden is higher and antimicrobial choices and usage are impacted by high levels of patient co-payments (Godman et al., 2021; Sulis et al., 2022; Khan et al., 2024; Lewnard et al., 2024; Sartorius et al., 2024; Saleem et al., 2025a).

In response to this critical public health issue, the World Health Organization (WHO, 2015) developed the Global Action Plan (GAP) on AMR, which led to the creation of National Action Plans (NAPs) aimed at encouraging countries to address the emerging public health threat of AMR (WHO, 2016). The purpose of the NAPs is to facilitate effective policy and stewardship measures to reduce the prevalence of AMR within each country (Godman et al., 2022; Willemsen et al., 2022; Charani et al., 2023). However, there have been concerns regarding the implementation of NAPs, especially in LMICs, due to resource constraints and shortage of trained personnel (Chua et al., 2021; Godman et al., 2022; Ohemu, 2022; Saleem et al., 2022). Notably, the fourth objective of NAPs emphasizes the development and implementation of clinical practice guidelines to promote the appropriate use of antimicrobials. These serve as tools for promoting rational prescribing practices and reducing the inappropriate use of antibiotics within countries. Clinical guidelines can be defined as documents that provide recommendations for clinical practice, aiming to minimize variability in care, particularly when scientific evidence is limited or when multiple therapeutic options are available (Steinberg et al., 2011; Johnson et al., 2021). The development and implementation of evidence-based guidelines support clinical decision-making by enhancing the quality of care, improving patient outcomes, and promoting the efficient use of resources (Sackett et al., 1996). The publication and implementation of antibiotic guidelines are regarded as important educational measures, especially for physicians in LMICs. This is because robust clinical guidelines are believed to significantly improve the quality of antibiotic prescribing across all sectors of care (Cooper et al., 2020; D’Arcy et al., 2021; Foxlee et al., 2021; Akhloufi et al., 2022; Boltena et al., 2023). As a result, adherence to published guidelines is increasingly incorporated into antimicrobial stewardship programs (ASPs) both in hospitals and primary care settings, often as part of agreed quality indicator targets (Versporten et al., 2018; Foxlee et al., 2021; Chigome et al., 2023; Funiciello et al., 2024; Lubanga et al., 2024). ASPs increasingly play a pivotal role in monitoring the implementation of national or regional guidelines to improve clinical outcomes across all sectors while minimizing the consequences of inappropriate antibiotic use (Nathwani et al., 2019; Brinkmann and Kibuule, 2020; Akhloufi et al., 2022; Siachalinga et al., 2022; Haseeb et al., 2023). ASPs in high-income countries (HICs) are often supported by electronic health records (EHRs)—digital systems that store patient medical information and can be integrated with clinical decision support tools. As a result, EHRs facilitate the monitoring of prescribing practices, facilitate audits, and help ensure adherence to national or institutional guidelines as part of ASPs. However, there have been concerns about implementing ASPs in LMICs due to resource limitations and personnel issues shortages; encouragingly, this is now beginning to change despite previous challenges (Cox et al., 2017; Brinkmann and Kibuule, 2020; Godman et al., 2021; Borde et al., 2022; Siachalinga et al., 2022).

Meanwhile, there is often a lack of national AMR surveillance, especially within LMICs, which limits the development of national evidence-based guidelines and the subsequent implementation of targeted policies to improve future antibiotic use (Woolf et al., 1999; Ohemu, 2022; Do et al., 2023; Okolie et al., 2023; Kiggundu et al., 2023; Mustafa et al., 2024). HICs often have access to resources for comprehensive guideline development, including local antimicrobial resistance data, systematic reviews, and advanced methodologies, including GRADE (Grading of Recommendations, Assessment, Development, and Evaluation) (Barker et al., 2021). Conversely, LMICs frequently rely on expert opinions and internationally derived guidelines to help improve antibiotic prescribing, which may not be well-adapted to local AMR patterns and healthcare contexts. Moreover, due to resource constraints, implementing such guidelines or establishing such programs is more challenging in LMICs. In addition to this, adherence to national or international guidelines remains highly variable, especially across LMICs, leading to inappropriate and excessive use of antibiotics, which may be a reflection of concerns about the local relevance of adapted guidelines (Sulis et al., 2020; Thi et al., 2024; Wang et al., 2022; Mahmudul Islam et al., 2024).

Consequently, there is a need to evaluate the availability and scope of current national and international antibiotic guidelines. It is important to identify disparities in their development, adaptation, and implementation. These insights can inform strategies to improve guideline adherence, particularly by assessing the extent to which local AMR patterns are incorporated into guideline updates. We acknowledge that the WHO AWaRe book has recently been published, covering 35 common infections across healthcare sectors; this builds on the WHO AWaRe classification, which is part of the Essential Medicines List (Moja et al., 2024; WHO, 2022; Sharland et al., 2019). However, this guidance may require local adaption based on local resistance patterns. In addition, many existing local or national guidance may be outdated, highlighting the urgent need for updates—especially in light of the recent United Nations General Assembly (UN-GA) target, which calls for 70% of antibiotics used across sectors to come from the Access group (United Nations, 2024). We are also aware that the WHO AWaRe classification system is increasingly being used across studies, including those conducted in LMICs, to assess current antibiotic use patterns and set targets for ASPs—a trend that is expected to continue (Saleem et al., 2025a; Saleem et al., 2025b).

In view of the rising global threat of AMR, coupled with the variability in national and local antibiotic prescribing practices, the first objective of this review was to assess the availability, scope, and quality of current antibiotic prescribing guidelines across countries of varying income levels, specifically to identify gaps in guideline development and adaptation. This review also aimed to evaluate the degree to which current guidelines incorporate the WHO AWaRe book recommendations and address key components such as patient education, AMR surveillance, and stewardship strategies (Funiciello et al., 2024; Moja et al., 2024). By identifying these gaps and emphasizing the importance of locally adapted, evidence-based guidelines, this review seeks to contribute to ongoing global efforts to optimize antibiotic use, reduce the reliance on broad-spectrum antibiotics, and assist in achieving the NAP goals, especially in LMICs. This is viewed as essential for meeting the UN-GA target of reducing AMR and increasing the use of Access antibiotics (United Nations, 2024).

## 2 Materials and methods

### 2.1 Study design

This study was conducted as a narrative review to comprehensively analyze antibiotic prescribing guidelines from diverse regions and income-level countries. The main objective was to identify disparities in guideline development, adaptation, and implementation, providing a robust understanding of current practices in antibiotic prescribing at local, national, and international levels. We have used this approach in multiple previous publications (Godman et al., 2021; Godman et al., 2022; Chigome et al., 2023; Haseeb et al., 2023) and believed this design is better suited to the heterogeneity of the included studies and the descriptive goals of the review, especially given the wide variation in regions and income levels included.

### 2.2 Search strategy

A comprehensive search was conducted between June and September 2024 to identify eligible antibiotic prescribing guidelines. The primary sources for data collection and extraction were Google Scholar and PubMed, alongside other search engines, with most of the data obtained directly from Google and official country websites. We first conducted a PubMed search using MeSH terms and Boolean operators, including “antibiotic prescribing guideline*,” “clinical practice guideline*,” or “antibiotic guideline*” in the title. Subsequently, we used Google as a search engine to locate guidelines not included in the medical literature but available online. This approach was conducted based on the assumption that a significant number of guidelines might be published by scientific societies or governmental agencies and made available on the internet without being captured by formal literature repositories.

We subsequently accessed possible antibiotic prescribing guidelines by analyzing official country websites. The search for guidelines on official country websites was conducted manually using a structured and systematic approach. For every country, the official website of the Ministry of Health, or equivalent national health authority, was identified via a Google search. Furthermore, country-specific AMR National Action Plan pages were examined as they often linked to or referenced available antibiotic prescribing guidelines. This method ensured that both recent and archived guidelines could be identified, even in countries with limited digital infrastructure or non-indexed resources.

#### 2.2.1 Eligibility criteria

A country was included if it had at least one publicly accessible antibiotic prescribing guideline available in English that met the review’s inclusion criteria. Countries were not excluded based on income level or geographic region.

#### 2.2.2 Guideline inclusion criteria

This review focused on bacterial infections and clinical syndromes that are commonly managed with antibiotic therapy. Inclusion criteria encompassed any English-language antibiotic prescribing guidelines from across all income countries that provided specific treatment recommendations, including antibiotic names, dosages, and durations. To ensure a comprehensive overview, local, national, and international guidelines were considered.

#### 2.2.3 Exclusion criteria

Guidelines were excluded if they focused solely on infection prevention or non-antibiotic therapies. Additionally, guidelines that lacked detailed prescribing information, such as those offering only general guidance without specifying antibiotic names, dosages, or durations, were excluded. Finally, documents not published in English were excluded due to translation constraints. In addition, English is recognized as the international scientific language.

### 2.3 Information sought for each guideline

**FIGURE 1:**
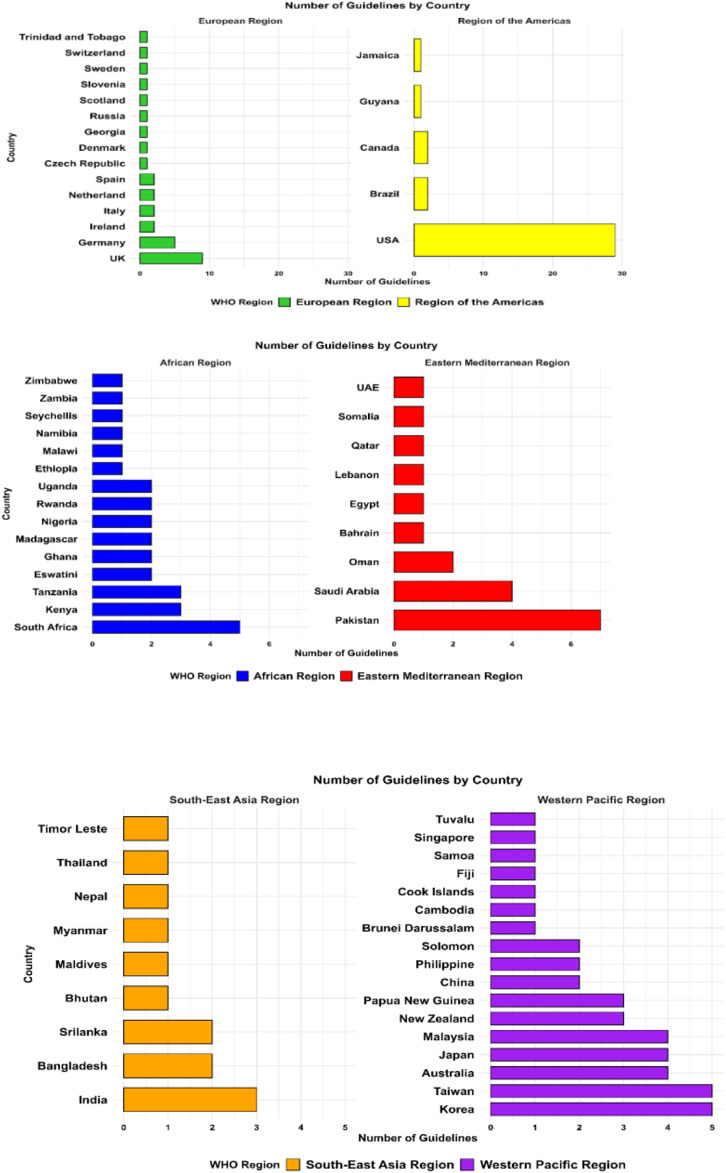
Geographical distribution of included antibiotic prescribing guidelines by WHO regions.

For each guideline included, we collected general information on the country of origin and its geographic location according to WHO regions and its income classification—i.e., low-income, lower-middle, upper-middle, and high-income—based on the World Bank classification (World Bank, 2025), consistent with our previous reviews (Saleem et al., 2025a; Saleem et al., 2025c). Additional data were extracted on the guideline title, year of publication, development methodology, issuing organization, target population, scope, and extent of local adaptation. We also noted whether the guidelines addressed current AMR patterns and included implementation strategies, compliance and monitoring, outcome measures, and patient education or counseling components. These key variables and their distribution across guidelines are visually summarized in Figure 1. Patient-related data were collected due to their recognized role in influencing antibiotic prescribing and dispensing, especially in LMICs (Nair et al., 2019; Antwi et al., 2020; Khan et al., 2020; Ramdas et al., 2025). Effective patient counseling is increasingly regarded as essential to enhance adherence to prescribed antibiotics, educate patients on the importance of completing treatment courses, and raise awareness of the risks associated with misuse (Saleem et al., 2025a; Saleem et al., 2025c; Balea et al., 2025; Sakeena et al., 2018; Hunter and Owen, 2024).

As part of our data extraction process, we also assessed whether each guideline utilized a structured evidence-based grading methodology, specifically the GRADE framework. For each guideline, we recorded whether GRADE was explicitly mentioned as part of the guideline development methodology. This included checking the methodology sections of the guidelines for references to GRADE terminology, use of evidence quality ratings (e.g., “low,” “moderate,” and “high” certainty), and the presence of structured recommendation grading. This enabled us to evaluate the extent to which evidence-based approaches were incorporated into the development of antibiotic prescribing guidelines by various country income groups. Each guideline was thoroughly reviewed to determine whether it included information on AMR patterns and patient counseling. As a result, we aimed to provide a thorough evaluation of current antibiotic guidelines and their applicability across various healthcare settings.

For the purpose of this review, a guideline was considered “outdated” if it was published more than 10 years before the date of data collection, i.e., prior to 2014, and showed no evidence of revision, update, or endorsement in more recent policy documents or on websites. This cutoff was chosen based on international best practices, which recommend regular updates to clinical guidelines every 3–5 years to ensure alignment with evolving evidence, AMR trends, and AMS practices.

### 2.4 Ethical considerations

Ethical approval was not needed for this study as we included only readily available published material and no patients were involved in the study.

## 3 Results

**FIGURE 2:**
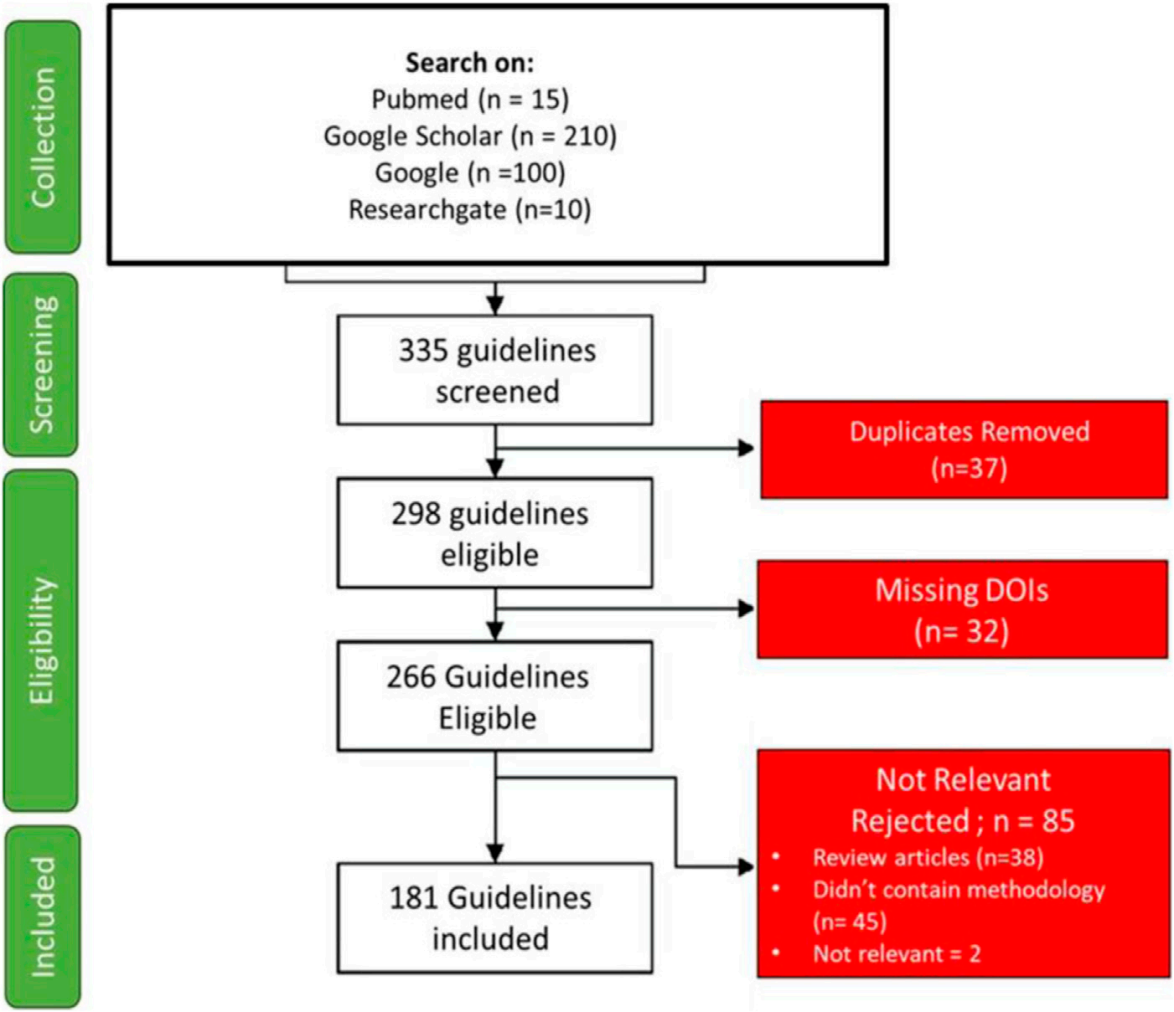
Flow diagram illustrating the process of identification, screening, eligibility assessment, and inclusion of antibiotic prescribing guidelines reviewed in this study.

We retrieved 335 antibiotic prescribing guidelines, with the majority of data obtained directly from Google and official country websites. Of these, 181 guidelines met our inclusion criteria, provided sufficient information for our review, and were subsequently described in detail. Figure 2 illustrates the guideline selection process.

The general characteristics of the included antibiotic prescribing guidelines are summarized in Table 1 and Supplementary Table S1.

**TABLE 1:** Characteristics of included guidelines in the review.

Characteristic	Number of guidelines (% total)
Income group	
HICs	109 (60.22)
UMICs	14 (7.73)
LMICs	40 (22.10)
LICs	18 (9.94)
Global	
European guidelines	13 (7.18)
WHO regions	
African Region	29 (16)
Eastern Mediterranean Region	19 (10.5)
European Region	31 (17.13)
Region of the Americas	35 (19.3)
Southeast Asia Region	13 (7)
Western Pacific Region	41 (23)
Scope	
International	45 (24.86)
National	116 (64.09)
Local	20 (11.05)
Grade methodology used	
Yes	37 (20.44)
No	144 (79.56)

NB: HIC, high-income country; UMIC, upper-middle income country; LMIC, lower-middle income country; and LIC, low-income country.

### 3.1 Distribution by income group and WHO region

**FIGURE 3:**
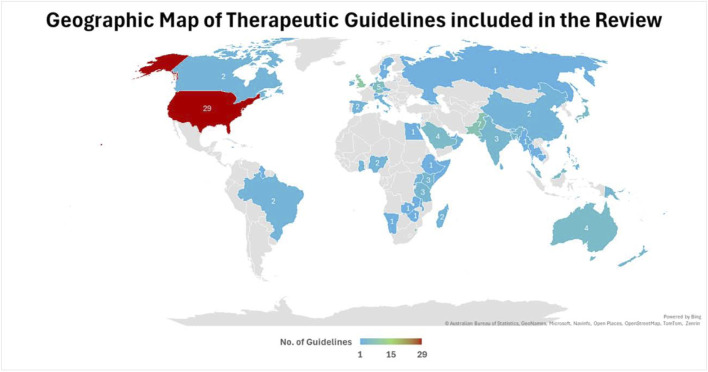
Geographical distribution of included antibiotic prescribing guidelines among the countries.

Most guidelines (n = 109; 60.2%) originated from HICs, followed by lower-middle-income countries with 40 guidelines (22.1%), low-income countries (LICs) with 18 guidelines (9.9%), and upper-middle-income countries with 14 guidelines (7.7%). The geographical distribution also varied significantly, with the European Region and the Region of the Americas contributing the majority of guidelines, while the African and Southeast Asian regions were underrepresented. Figure 3 illustrates the geographical distribution of antibiotic prescribing guidelines among the countries worldwide.

### 3.2 Methodological approaches and use of GRADE

Out of the 181 guidelines, only 37 (20.4%) explicitly referenced the use of the GRADE framework. The majority of these were from HICs, reflecting higher methodological standards and resources for evidence synthesis (Jackson et al., 2020; NICE CAP Guideline, 2019a; Autore et al., 2023; Sanford Guideline, 2024). For example, the UK’s NICE and the USA’s IDSA guidelines extensively apply the GRADE methodology to support transparency and methodological rigor (NICE Guidelines, 2023; Tamma et al., 2024).

In contrast, most guidelines from LMICs relied on expert consensus or non-transparent development processes, often lacking structured grading of recommendations (Bartlett et al., 1998; BSMMU Guideline Bangladesh, 2023; Antibiotic Guidelines Myanmar, 2019). For example, guidelines from Pakistan and Bhutan primarily relied on literature reviews without systematic evidence-based grading, resulting in broad recommendations (MMIDSP Guideline, 2022; Ministry of Health Bhutan, 2018). Details are mentioned in Table 2 and Supplementary Table S2.

**TABLE 2:** Global variation in antibiotic prescribing guidelines.

Global guidelines	Guideline abbreviation	Publication date	Methodology	Prepared by	Scope	Reference
Europe	EAU GUIDELINES UI	2024	Evidence-based review and expert consensus	EAU	International	Bonkat et al. (2017)
	ESCMID ED ST Guidelines	2024	Evidence-based review and expert consensus	ESCMID and EAHP	International	Schoffelen et al. (2024)
	ERS/ESICM/ESCMID/ALAT CAP-Guidelines	2023	GRADE	ERS/ESICM/ESCMID/ALAT	National	Martin-Loeches et al. (2023)
	ESCMID–EUCIC Guidelines	2019	Evidence-based review and expert consensus	ESCMID and EUCIC	International	Tacconelli et al. (2019)
	ERS guidelines for AB	2017	GRADE	ERS	International	Polverino et al. (2017)
	ERS-HAP/VAP Guidelines	2017	GRADE	ERS/ESICM/ESCMID/ALAT	International	Torres et al. (2017)
	ESCMID Meng. Guidelines	2016	GRADE	Microbiology ESCMID	International	Van de Beek et al. (2016)
	ESMO-febrile neutropenia	2016	GRADE	ESMO	International	Klastersky et al. (2016)
	Blue Book	2016	Evidence-based review and expert consensus	OUP and ESPID	International	Butler (2016)
	ESC-Endocarditis-G	2015	Evidence-based expert consensus	ESC-EACTS and EANM	International	Habib et al. (2016)
	EAU/ESPU-UTI	2015	Literature review (evidence-based)	EAU/ESPU	International	Stein et al. (2015)
	EAU UTI Guidelines	2014	GRADE	EAU	International	Debast et al. (2014)
	ESCMID-ST Guidelines	2012	GRADE	ESCMID/ESCMID STG	International	Pelucchi et al. (2012)

Income level	Country	Guideline abbreviation	Publication date	Methodology	Prepared by		Scope	Reference
African Region								
HIC	Seychelles	Seychelles STGs	2003	Combined local medical practice experience with relevant international standards	MOH		National	Ministry of Health Seychelles (2003)
UMIC	Namibia	Namibia STG	2011	Evidence based guidelines	MOH and SC		National	Ministry of Health Namibia (2011)
LMIC	Eswatini	Eswatini STG-EML 2020	2020	Evidence-based recommendations aligned with WHO EML	MOH		National	Ministry of Health Eswatini (2020)
		Eswatini STG-EML 2012	2012	Evidence-based guidelines	MOH		National	Ministry of Health Eswatini (2012)
	Ghana	Ghana COVID-19 STG	2020	Evidence-based review, expert consensus, and local AMR	MOH		National	Ministry of Health Ghana (2020)
		GNDP STG	2017	Based on evidence quality RCTs, clinical studies, and expert opinions	MOH		National	Ministry of Health Ghana (2017a)
	Kenya	Kenya AMS Guidelines	2020	Based on AMS spectrum, development, and implementation of systems and interventions	MOH		National	Ministry of Health Kenya (2020)
		Kenya AMS Protocol	2020	Multiregional stepwise stewardship intervention: 18-month ASP adaptation	KNRF		Local	Gitaka et al. (2020)
		Kenya AMS Guidelines	2012	GRADE	MMS		National	Agweyu et al. (2012)
	Nigeria	Nigeria ICU Guidelines	2023	Based on evidence-based published data and local prospective antibiograms	Expert Committee		National	Oladele et al. (2023)
		Nigeria STG	2016	Evidence-based guidelines	MOH		National	Ministry of Health Nigeria (2016)
	Tanzania	STG/NEMLIT	2021	NEMLIT new edition aligns with WHO recommendations	MOH/CD		National	Ministry of Health Tanzania (2021)
		AMS Policy Guidelines	2020	Evidence-based recommendations aligned with WHO AMR Action Plan and local data	MOH		National	Ministry of Health Tanzania (2020)
		STG-NEMLIT	2017	Evidence-based recommendations, expert consensus, and local AMR data	MOH		National	Ministry of Health Tanzania (2017)
	Zambia	ENC Guidelines	2014	Evidence-based guidelines	MCD/MCH		National	Ministry of Health Zambia (2014)
LIC	Ethiopia	Ethiopia PH STGs	2014	Evidence-based recommendations aligned with WHO EML and local disease burden	MOH		National	Ministry of Health Ethiopia (2014)
	Malawi	MSTG 5th Edition	2015	Evidence-based guidelines	MOH		National	Ministry of Health Malawi (2015)
	Madagascar	(Madagascar IG) Guide	2020	Evidence-based, expert consensus, and local disease burden	Ministry of Public Health		National	Ministry of Health Madagascar (2020)
		Antibiotika Tsara	2018	Digital tool combining local AMR data and stewardship algorithms	SPIM		Local	SPIM. Antibiotika Tsara (2018)
	Rwanda	Rwanda Guidelines	2022	Based on available evidence and literature review	MOH		National	Ministry of Health Rwanda (2022)
		Rwanda COVID-19 Guidelines	2020	Evidence-based expert consensus in alignment with WHO recommendations	RBC		National	Ministry of Health Rwanda (2020)
	South Africa	SA Hospital AMR Guidelines	2023	Policy framework aligned with WHO and national AMR action plans	NDH		National	Department of Health South Africa (2023)
		Africa CDC Guidelines	2021	Evidence-based expert consensus in alignment with local AMR data	Africa CDC and OHT		International	African Union (2021)
		AMPATH Guidelines	2017	Laboratory-driven, local antibiograms and diagnostic protocols	AMPATH		National	AMPATH Antibiotic Guide (2017)
		SA CAP Guidelines	2017	Evidence-based expert consensus	SATC		National	Boyles et al. (2017)
		SAASP Pocket Guide	2014	Evidence-based expert consensus and local resistance data	SAASP		National	SAASP (2014)
	Uganda	Uganda Clinical Guidelines	2023	Evidence-based expert consensus	MOH		National	Ministry of Health Uganda (2023)
		Uganda Guidelines	2020	Ugandan approach combines WHO guidance, national policies, and local innovation	MOH		National	Ministry of Health Uganda (2020)
	Zimbabwe	EDLIZ Guidelines	2015	Evidence-based guidelines	NMTPAC/MOH		National	Ministry of Health Zimbabwe (2015)
Eastern Mediterranean Region								
HIC	Bahrain	TG and pathways	2022	Evidence-based guidelines	NHRA		National	NHRA Bahrain (2022)
	Oman	Oman AMS Guidelines	2016	Evidence-based recommendations and expert consensus aligned with international standards	MOH		National	Ministry of Health Oman (2016)
		Oman SAP Guidelines	2015	National Antimicrobial Subcommittee (surgical team practice-based)	MOH		National	Ministry of Health Oman (2015)
	Qatar	Qatar CAP Guidelines	2016	Evidence-based recommendations aligned with WHO standards	MOH		National	Ibrahim (2016)
	Saudi Arabia	UTI infection protocol	2023	Evidence-based recommendations aligned with IDSA, WHO, and local AMR	MOH		National	Ministry of Health Saudi Arabia (2023)
		Lower RTI protocol	2020	Evidence-based expert consensus	MOH		National	Ministry of Health Saudi Arabia (2020)
		NAT-G for CAP–HAP Adults	2018	Evidence-based selection and local epidemiology of antimicrobial resistance	MOH		National	Ministry of Health Saudi Arabia (2018)
		Saudi CAP In adults	2017	Evidence-based recommendations, expert consensus, and literature review	SPIDS		National	Alzomor et al. (2017)
	UAE	UAE IAI Guidelines	2022	Evidence-based recommendations aligned with local AMR data	N-ASC		National	Ministry of Health UAE (2022)
LMIC	Egypt	Egypt Preauthorization Guidelines	2022	Based on Egyptian Hospital Antimicrobial Data	EDA/NARC		National	Egypt Drug Authority Guideline (2022)
	Lebanon	LSIDCM CAP Guidelines	2014	Evidence-based recommendations adapted to Lebanese local AMR	LSIDCM		National	Moghnieh et al. (2014)
	Pakistan
		Bahria Hospital Guidelines	2023	Hospital formulary and stewardship-driven recommendations	BIH		Local	Bahria International Hospital Guideline (2023)
		Typhoid Management Guidelines	2022	Evidence-based local resistance surveillance and expert consensus	MMIDSP		National	MMIDSP Guideline (2022)
		DRAP Guidelines	2022	Regulatory framework aligned with WHO Global Action Plan on AMR	DRAP		National	DRAP Guidelines (2022)
		PCS COPD Guideline	2020	Evidence-based	PCS		Local	Pakistan Chest Society Guideline (2020)
		MMIDSP IDSP and PARN Guidelines	2019	Evidence-based recommendations and expert consensus	MMIDSP IDSP/PARN		Local	IDSP and PARN Guidelines (2019)
		PCS Guidelines for CAP in adults	2017	Evidence-based expert consensus and local resistance data	PCS		National	Bokhari et al. (2017)
		SGP Pakistan	2015	Evidence-based locally adapted sepsis management	AKU		Local	Hashmi et al. (2015)
LIC	Somalia	Somali STGs	2015	Evidence-based data aligned with WHO EML	MOH		National	Ministry of Health Somalia (2015)
European Region								
HIC	Czech Republic	Czech CDI Guidelines	2022	Evidence-based guideline updated version	DID		Local	Beneš et al. (2022)
	Denmark	Danish Antibiotic Guidelines	2013	Evidence-based practices, laboratory testing, and rational use of antibiotics	DHMA		National	Danish Health and Medicine Authority Guidelines (2013)
	Germany	German LRTI Guidelines	2024	Clinical experts collaborated on recommendations	GSPID/AWMF		National	Mauritz et al. (2024)
		Pediatric CAP Guidelines	2020	Evidence-based review and expert consensus	PCAP-DGPI/GPP		National	Rose et al. (2020)
		German ABS Guidelines	2016	Evidence-based grading according to the AWMF Guidance Manual	GSID		International	De With et al. (2016)
		GT Guidelines	2015	Evidence-based review, expert consensus	GSO and HNS		National	Windfuhr et al. (2016)
		German CAPNETZ Guidelines	2009	Key points based on the Oxford Center for evidence-based structures	Paul-Ehrlich-SC, GRS, GSI, and CAPNETZ		National	Höffken et al. (2009)
	Ireland	CHI-Antimicrobial Guidelines	2020	Evidence-based recommendations, local resistance data, and expert consensus	CHI		National	CHI Guideline Ireland (2020)
		PiPc children Guidelines	2016	Two-round modified Delphi consensus method	DGP RCS/CPRG		Local	Barry et al. (2016)
	Italy	UTI-Ped-ER Guidelines	2023	GRADE	UTI-Ped-ER		Local	Autore et al. (2023)
		SITA-SIP COVID-19 Guidelines	2021	GRADE	SITA/SIP		National	Bassetti et al. (2021)
	Netherlands	SWAB CAP Guidelines	2024	GRADE	SWAB/NVALT		National	Guideline Netherlands (2024)
		CDSS-Antibiotics-2022	2022	Developed CDSS for empirical antibiotic therapy, involving stakeholders	Erasmus MC and UMC		Local	Akhloufi et al. (2022)
	Russia	COPD Guidelines	2018	Evidence-based review and expert consensus	RRS		National	Aisanov et al. (2018)
	Scotland	CDI Guidelines	2014	Evidence-based recommendations and expert consensus	NHS, HPN, and HPS		National	Health Protection Scotland (2014)
	Slovenia	Slovenia Neurosurgical Guidelines	2014	Evidence-based recommendations for neurosurgical prophylaxis	SMA		National	Slovenian Guidelines (2014)
	Spain	MSF Clinical Guidelines	2024	Evidence-based, context-adapted protocols for resource-limited settings	MSF		International	MSF Clinical guidelines (2024)
		V Spanish Consensus Guidelines	2022	Systematic review of scientific evidence, Delphi process, and GRADE	VSCC		National	Gisbert et al. (2022)
	Sweden	Swedish CAP Guidelines	2012	Evidence-based review and expert consensus	SSID		National	Spindler et al. (2012)
	Switzerland	Swiss PAP Guidelines	2022	Evidence-based, database-informed surgical prophylaxis	PIGS		National	Paioni et al. (2022)
	Trinidad and Tobago	T&T ARI Guidelines	2020	GRADE	MOH		National	Nagassar (2020)
	UK	GMMMG Guidelines	2024	Evidence-based	GM HCCM		Local	Greater Manchester (2024)
		BSW ICB Guidelines	2024	Evidence-based	BSW-ICB		Local	BSW ICB Guidelines UK (2024)
		Pneumonia NICE Guidelines	2023	GRADE	NICE		International	NICE Guidelines (2023)
		BTS Guidelines	2020	GRADE	BTS		National	Smith et al. (2020)
		UK BASH HIV NG	2020	Evidence-based review and expert consensus	BASHH		National	Chirwa et al. (2021)
		AMP Dentistry-GPG	2020	GRADE	FDS-RCS		National	GP Guidelines UK (2020)
		HAP NICE Guidelines	2019	Evidence-based review and expert consensus	NICE		International	NICE HAP Guideline (2019)
		CAP-APG	2019	GRADE	NICE		International	NICE CAP Guideline (2019)
		BSAC Guidelines ED	2012	Evidence-based review, expert consensus	BSAC		National	Gould et al. (2012b)
UMIC	Georgia	GPAS CAP Guidelines	2023	Evidence-based review and expert consensus	Children’s healthcare		National	Georgia Pediatric Antibiotic Stewardship (2023)
Region of the Americas								
HIC	Canada	CCPG-rhinosinusitis	2011	Evidence-based review and expert consensus		CSOHNS and CRWG	National	Desrosiers et al. (2011)
		CA-MRSA-2006	2006	Evidence-based review and expert consensus		CMA and CIDS	Local	Barton et al. (2006)
	United States	IDSA Guidelines	2024	GRADE		IDSA	International	Tamma et al. (2024)
		Sanford-VAP	2024	Evidence-based recommendations		Jay P. Sanford	International	Sanford Guideline (2024)
		CDC-Antibiotic-	2024	Evidence-based practices and expert consensus		CDC	National	CDC Guidelines (2024)
		SCCM-corticosteroids	2024	GRADE		SCCM	international	Chaudhuri et al. (2024)
		IWGDF/IDSA-DFI	2023	GRADE		IWGDF and IDSA	International	Senneville et al. (2024)
		Michigan UTI Guidelines	2021	Evidence-based review and expert consensus		MHHA	International	MHHA Guideline (2021)
		AAP-Red Book-	2021	GRADE		AAP	International	Red Book (2021)
		IWGDF/IDSA-DFI	2020	GRADE		(IWGDF/IDSA	International	Lipsky et al. (2020)
		ATS CAP Guideline	2019	GRADE		ATS &IDSA	International	Jackson et al. (2020)
		IDSA OPAT Guidelines	2018	GRADE		IDSA	International	Norris et al. (2019)
		IDSA Diarrhea	2018	GRADE		IDSA	International	Randel (2018)
		IDSA/SHEA CDI Guidelines	2017	GRADE		IDSA/SHEA	International	McDonald et al. (2018)
		Adult and pediatric APG	2017	Evidence-based and systematic review consensus		DOH	National	CDC (2017)
		AAO-HNS-OME	2016	Evidence-based review and expert consensus		AAO-HNS	International	Rosenfeld et al. (2016)
		ACG-diarrhea-	2016	Evidence-based review and expert consensus		ACG	International	Riddle et al. (2016)
		ACP/CDC-ARTI	2016	Evidence-based practices		ACP/CDC	National	Bredemeyer (2016)
		AAO-HNS Guidelines	2015	GRADE		AAO-HNS	International	Rosenfeld et al. (2015)
		IDSA-SSTI-Guidelines	2014	Evidence-based review and expert consensus		IDSA	International	Stevens et al. (2014)
		ASHP-surgical	2013	GRADE		ASHP, IDSA, SIS, and SHEA	International	Bratzler et al. (2013)
		IDSA ABRS Guidelines	2012	GRADE		IDSA	International	Chow et al. (2012)
		AAP-UTI	2011	GRADE		AAP	International	Roberts et al. (2011)
		CAP Guidelines PIDS/IDSA	2011	GRADE		PIDS and IDSA	International	Bradley et al. (2011)
		ACC/AHA-IE-	2008	Evidence-based and expert consensus		ACC/AHA	International	Nishimura et al. (2008)
		AAO-HNS-sinusitis-	2007	Evidence-based recommendations		AAO-HNS	International	Rosenfeld et al. (2007)
		ACCP CG	2006	GRADE		ACCP	International	Braman (2006)
		IDSA GAS-pharyngitis	2002	Evidence-based systematic review consensus		IDSA	International	Bisno et al. (2002)
		IDSA-ID-	2001	Evidence-based systematic review consensus		IDSA	International	Guerrant et al. (2001)
		AAP-CPG-sinusitis	2001	Evidence-based systematic review consensus		AAP	National	Pediatrics (2001)
		IDSA CAP Guidelines	1998	Expert consensus and literature review		CID and IDSA	International	Bartlett et al. (1998)
UMIC	Brazil	Brazilian CAP Guidelines	2009	Evidence-based recommendations, expert consensus, and literature review		BTS	National	Corrêa et al. (2009)
		Brazilian CAP Guidelines	2004	Evidence-based recommendations and expert consensus		BSP	National	Nascimento-Carvalho and Souza-Marques (2004)
	Guyana	Guyana STG	2015	Evidence-based recommendations, expert consensus, and local AMR		MOH	National	Ministry of Health Guyana (2015)
	Jamaica	Jamaican UTI Guidelines	2018	Evidence-based review and expert consensus		JKKF	National	Young Peart et al. (2018)
Southeast Asia Region								
UMIC	Maldives	Maldives NAMSP	2020	National policy framework for antimicrobial stewardship and resistance control	MOH		National	Ministry of Health Maldives (2020)
	Thailand	Asthma Guidelines	2022	Evidence-based	TAC		National	Kawamatawong et al. (2022)
	Bangladesh	BSMMU Guidelines	2023	Evidence-based recommendations aligned with local antibiograms	BSMMU/WHO		National	BSMMU Guideline Bangladesh (2023)
		Bangladesh STG	2021	Evidence-based protocols aligned with WHO standards	MOH		National	Ministry of Health Bangladesh (2021a)
		AP for BIRDEM Hospital	2021	Hospital-specific recommendations based on local AMR data and expert consensus	BGH		Local	BIRDEM Hospital Bangladesh (2021)
LMIC	Bhutan	Bhutan-ABG	2018	Evidence-based review and expert consensus	MOH		National	Ministry of Health Bhutan (2018)
	Timor-Leste	Antibiotic Guidelines HNG	2016	Evidence based guidelines	HNGV		National	Hospital Guidelines Timor Leste (2016)
	India	Indian ICU-IC Guidelines	2019	Evidence-based recommendations, systematic reviews, and expert consensus	ICMR/CCS		Local	Kulkarni et al. (2019)
		ICU Antibiotic Guidelines	2019	Evidence-based expert consensus aligned with local AMR data	ISCCM		National	Khilnani et al. (2019)
		India AMR Guidelines	2016	Evidence-based protocols aligned with WHO GAP	NCDC/MOH		National	Ministry of Health Guideline India (2016)
	Myanmar	Myanmar NOGTH Guidelines	2019	Evidence-based recommendations aligned with WHO standards to combat AMR	GTH and WHO		Local	Antibiotic Guidelines Myanmar (2019)
	Sri Lanka	Sri Lanka AMR Guidelines	2024	Evidence-based guidelines	SCM/MOH/NIM		National	Ministry of Health Srilanka (2016)
		SLMA Guidelines	2014	Evidence-based guidelines	SLMA		National	SLMA Guidelines (2014)
Western Pacific Region								
HIC	Australia	TG Antibiotic Guidelines	2024	Evidence-based recommendations	TGL		National	Therapeutic Guidelines Australia (2024)
		CHQ-P Antibiocard	2024	Initial treatment recommendations, AS, daily review, and TDM.	CHQ-P		Local	CHQHospital. Children (2022)
		SAP Guidelines	2021	Evidence-based review and expert consensus	Govt. SAAGAR		Local	SAP Guideline Australia (2021)
		KHA-CARI UTI Guidelines	2015	GRADE	KHA-CARI		National	McTaggart et al. (2015)
	Brunei Darussalam	Brunei Darussalam GAPP	2019	Stewardship-focused recommendations and expert consensus aligned with WHO standards	MOH		National	Ministry of health Brunei Darussalam (2019)
	Cook Islands	Cook Islands-ABG	2023	Evidence-based local AMR data	MOH		National	Ministry of Health Cook Islands (2023)
	Japan	JGA CD Guidelines	2023	Evidence-based recommendations, expert consensus, and local data	JGA		National	Ihara et al. (2024)
		JSSI Guidelines	2021	GRADE Delphi method	JSSI		International	Ohge et al. (2021)
		Japan AMS Manual	2017	Evidence-based recommendations, expert consensus, and RCTS.	MOH/LWHSBT/IDCD		National	Ministry of Health Japan (2017)
		JRS CAP Guidelines	2006	Evidence-based recommendations, expert consensus, and local data	JRS		National	Miyashita et al. (2006)
	Korea	Korean AGE Guidelines	2019	GRADE	KSID/KSAD		National	Kim et al. (2019)
		Korean UTI Guidelines	2018	Evidence-based local resistance and expert consensus	KSID		National	Kang et al. (2018)
		KGU-CAP-	2018	Evidence-based review and expert consensus	KGU		National	Lee et al. (2018)
		KGU-ARTI	2017	Evidence-based review, expert consensus	KGU		National	Yoon et al. (2017)
		Korea BJI Guidelines	2014	GRADE	KSC		National	Korean Society for Chemotherapy et al. (2014)
	New Zealand	BPAC^nz^primary care AG	2024	Evidence-based review, expert consensus, and local AMR	BPAC^nz^		Local	bpacnz Guide New Zealand (2024)
		BPAC^nz^-ABGuide	2017	Evidence-based review and expert consensus	BPAC		National	bpacnz Guide New Zealand (2017)
		ANZPID-ASAP Guidelines	2016	Evidence-based recommendations, expert consensus, and local AMR	ANZPID-ASAP		National	ANZPID-ASAP Guideline (2016)
	Singapore	Singapore SAP Guidelines	2022	ADAPTE method and evidence-based grading	NCID		National	Chung et al. (2022)
	Taiwan	Taiwan MDRO Guidelines	2022	Evidence-based recommendations, expert consensus, and local Ardita	TSM and iDST		National	Sy et al. (2022)
		Taiwan UP Guidelines	2011	Evidence-based consensus guidelines aligned with international standards and local AMR data	TUA		National	Chou et al. (2011)
		Taiwan CAP Guidelines	2008	Based on epidemiologic data, clinical studies, laboratory investigations, and imaging studies	TPA		National	Lee et al. (2007)
		Taiwan Surgical Prophylaxis Guidelines	2004	Evidence-based recommendations, expert consensus, and local resistance data	IDSC/TSA		National	IDSC Taiwan Surgical Association (2004)
		Taiwan UTI Guidelines	2000	Based on expert consensus and review of the existing literature	IDSC/TSA		National	IDSC Taiwan Guidelines (2000)
UMIC	China	Chinese HAP/VAP Guidelines	2018	GRADE	CTS and CMA		National	Shi et al. (2019)
		CTS CAP Guidelines	2016	Evidence-based recommendations, expert consensus, and local data	CTS and CMA		National	Cao et al. (2018)
	Fiji	Fiji Antibiotic Guidelines	2019	Evidence-based expert opinion	MOH and MSGF		National	Ministry of Health Fiji (2019a)
	Malaysia	Malaysia NAG	2024	Evidence-based guidelines evolve over time	MOH		National	Ministry of Health Malaysia (2024)
		Malaysia NAG	2014	Aligns with the AMS Program and incorporates updated evidence on AMR	MOH		National	Ministry of Health Malaysia (2014)
		PPUKM Guidelines	2012	Interdisciplinary panel updates evidence-based guidelines	MOH		National	PPUKM Malaysia Guideline (2012)
		Malaysia NAG	2008	Based on current evidence, drug formulary, and AMR patterns	MOH		National	Ministry of Health Malaysia (2008)
LMIC	Cambodia	CPG SAT guidelines	2016	Revision of the original practice guidelines	SHCH/HMC		International	SHCH/HMC (2016)
	Papua New Guinea	PNG HIV Guidelines	2019	Evidence-based recommendations, expert consensus, and data	NDOH		National	National department of Health Papua New Guinea (2019)
		PNG Pediatric STGs	2016	Evidence-based expert review	PSPG		National	STGs Papua New Guinea (2016)
		PNG Adult STGs	2012	Evidence based scoping reviews, field research, and epidemiological principles	NDOH/WHO		National	NDOH/WHO Papua New Guinea (2012)
	Philippines	PIDS Antibiotic Guidelines	2017	Review of evidence-based local and international guidelines and literature	NAGCOM		National	NAGCOM Philippine Guideline (2017)
		PSMID UTI Guidelines	2015	Evidence-based recommendations, expert consensus, and local AMR	PSMID		National	PSMID UTI Guidelines Philippine (2015)
	Samoa	PIOA Guidelines	2017	Evidence-based guidelines	PIOA		Local	PIOA Guidelines Samoa (2017)
LIC	Tuvalu	TST Guidelines	2010	Evidence-based practice	MOH		National	Ministry of Health Tuvalu (2010)
	Solomon	Pacific Islands Pediatric STGs	2017	Evidence-based pediatric care protocols	UNICEF and WHO		National	STGs Solomon (2017)
		Solomon Islands NCD Guidelines	2011	Evidence-based, WHO recommendations and local and international input	MOH/MS and WHO		National	Ministry of Health Solomon (2011)

NB: HIC, high-income country; UMIC, upper-middle income country; LMIC, lower-middle income country; and LIC, low-income country.

### 3.3 Scope and coverage of guidelines

Antibiotic prescribing guidelines differed substantially in scope, focus, and strategy as a result of variations in epidemiology, cultural context, and healthcare infrastructure within the regions. Guidelines from HICs were generally more detailed, including diagnostic criteria, age-specific dosing, and alternative regimens. For example, USA IWGDF/IDSA guidelines for diabetic foot infection incorporate pathogen-specific approaches (Senneville et al., 2024), while guidelines from LMICs, such as those from Ethiopia and Malawi, typically use syndromic management protocols without referencing pathogen-specific resistance data (Ministry of Health Ethiopia, 2014; Ministry of Health Malawi, 2015) (Supplementary Table S2).

### 3.4 Implementation and monitoring strategies

Only a limited number of guidelines, primarily from HICs, included implementation strategies such as audit and feedback mechanisms, performance indicators, or integration with EHR systems (Jackson et al., 2020; NICE CAP Guideline, 2019; Bisno et al., 2002; NICE HAP Guideline, 2019). For instance, the Netherlands and Sweden have institutionalized prescribing audits within their national EHR infrastructure to support stewardship programs (Akhloufi et al., 2022; Spindler et al., 2012). In contrast, guidelines from many LMICs, such as Nigeria and Bangladesh, lacked such structured implementation frameworks, largely due to limited digital health infrastructure and financial constraints (Ministry of Health Nigeria, 2016; Ministry of Health Bangladesh, 2021a).

### 3.5 Patient education and communication features

The majority of guidelines emphasized the importance of patient counseling. However, those from LMICs often lacked modern communication strategies to support effective implementation. Patient education was notably underrepresented, particularly in LMIC guidelines. HIC guidelines, such as those from the UK and Canada, commonly include patient-facing leaflets, risk communication tools, and checklists to facilitate counseling (Greater Manchester, 2024; Desrosiers et al., 2011). In contrast, guidelines from LICs such as Malawi and Ethiopia rarely provide structured education materials, contributing to gaps in patient engagement and adherence (Ministry of Health Ethiopia, 2014; Ministry of Health Malawi, 2015). Moreover, modern communication tools such as mobile applications, SMS-based adherence reminders, and visual aids, including infographics, were rarely utilized, limiting the potential for patient engagement and behavior change in these settings (see Supplementary Table S2).

### 3.6 AMR surveillance and local adaptation

Guidelines from HICs typically incorporated recent AMR data into their recommendations. For example, national guidelines from Australia and the Netherlands rely on routine national antibiograms (Guideline Netherlands, 2024; Therapeutic Guidelines Australia, 2024). Conversely, many LMIC guidelines, such as those from Eswatini and Ethiopia, are considered outdated as they were published over 10 years ago and made no reference to local surveillance data or antibiograms (Ministry of Health Ethiopia, 2014; Ministry of Health Eswatini, 2012). This lack of local data hampers effective empiric prescribing and contributes to the overuse of broad-spectrum antibiotics in these countries. Supplementary Table S2 provides examples of guideline recency and AMR data inclusion by income group.

## 4 Discussion

Since the development of antibiotic prescribing guidelines is usually an expensive and time-consuming process, requiring an expert’ team developed on the basis of the best available evidence, systematic reviews, and sound clinical understanding, there are likely to be disparities among countries with different income levels. HICs generally had more extensive and consistently updated clinical practice guidelines (CPGs) compared to LMICs, largely due to greater demand for care and better access to the resources, infrastructure, and expertise required for the development of such guidelines (Owolabi et al., 2018). For example, guidelines from the UK’s NICE, the US IDSA, and the Netherlands’ SWAB are based on rigorous methodologies, frequent updates, and comprehensive AMR integration (Greater Manchester, 2024; Tamma et al., 2024; Guideline Netherlands, 2024). Meanwhile, guidelines from LMICs, such as Nigeria’s National STGs and Ethiopia’s Standard Treatment Guidelines, particularly those from LICs, fell short in terms of coverage, quality, and content (Ministry of Health Ethiopia, 2014; Ministry of Health Nigeria, 2016).

As evidence-based decision-making has become a global standard for health interventions, the GRADE framework is increasingly used for the development of clinical guidelines (Baral et al., 2012; Park et al., 2015; Boon et al., 2021). HICs, supported by robust infrastructure and expert panels, have extensively implemented GRADE to provide transparency and methodological strength in recommendations (Bayona et al., 2017). For example, guidelines developed by the Infectious Diseases Society of America (IDSA), the American Thoracic Society (ATS), and UK bodies such as NICE and the British Thoracic Society (BTS) used GRADE consistently to integrate high-quality evidence and improve the implementation of antibiotic prescribing protocols (Jackson et al., 2020; NICE Guidelines, 2023; Smith et al., 2020; Gould et al., 2012a). In contrast, LMICs often rely on expert consensus due to limited resources, training, and access to evidence (Mzumara et al., 2023). Although several LMICs, including Pakistan, Ghana, Timor-Leste, and Rwanda, have developed evidence-based national treatment guidelines, their development is typically challenged by limited surveillance data, restricted funding, and suboptimal laboratory infrastructure (MMIDSP Guideline, 2022; Ministry of Health Ghana, 2017a; Hospital Guidelines Timor Leste, 2016; Ministry of Health Rwanda, 2022). These limitations also impede the routine use of systematic reviews and localized AMR data to inform prescribing.

Strengthening national efforts to combat AMR requires the development of guidelines that are both globally informed and locally applicable, as emphasized by the WHO. However, many LMICs lack the necessary infrastructure to support such efforts, particularly in terms of robust AMR surveillance systems. In these countries, either there is no routine national antibiogram reporting or the systems are fragmented and limited to isolated institutions (Gandra et al., 2020; Peters et al., 2024; Iskandar et al., 2021). A strong surveillance system typically includes nationwide coverage alongside standardized data collection, timely reporting, and integration with treatment guideline development (Nsubuga et al., 2011; Yang et al., 2020). The absence of these features in several LMICs undermines the ability to track resistance patterns and update empiric treatment guidelines accordingly (Gandra et al., 2020). Consequently, prescribers might find it challenging to match their practices with the WHO AWaRe book guidance; however, the AWaRe system provides a good starting point, particularly given its increasing use to monitor antibiotic use, including among LMICs, and the ongoing efforts to strengthen surveillance systems in these settings (Do et al., 2023; Iskandar et al., 2021; Tawfick et al., 2023). As a result, many LMICs are now working to implement more effective resistance surveillance in line with WHO goals. Local adaptation of the AWaRe guidance is key to support its use and enhance the effective management of infectious diseases. Local resistance trends contained within national and regional AMR surveillance data must inform first-line treatment choices, ensuring that guideline recommendations reflect local resistance profiles rather than relying merely on global trends (Mastrangelo et al., 2021). For instance, Rwanda’s susceptibility-guided empiric therapy and Kenya’s formulary mapping provide an example of how national guidance can be customized based on local data (Ministry of Health Rwanda, 2022; Gitaka et al., 2020). However, many LMICs lack comprehensive antibiograms, resulting in empiric overuse of broad-spectrum antibiotics, which should now be limited under the WHO AWaRe framework and guidance. This was evident in several guidelines included in our review. This included those from Nigeria, Ethiopia, and Malawi, which provided syndromic treatment recommendations without referencing local resistance data (Ministry of Health Ethiopia, 2014; Ministry of Health Malawi, 2015; Ministry of Health Nigeria, 2016). Addressing this gap involves investment in laboratory infrastructure and the training of healthcare professionals to correctly interpret and apply surveillance (Nishimura et al., 2008). This is important if LMICs are to achieve their AMR goals within their NAPs.

In HICs, AMS initiatives usually involve EHR audits, rapid diagnostics, and multidisciplinary stewardship teams to enhance appropriate antibiotic use. These technologies and institutional structures are usually lacking in LMICs. Scaling up such efforts across sectors will be critical to limiting unnecessary empiric antibiotic use and enhancing treatment outcomes (Bankar et al., 2022).

Financial barriers may also restrict the implementation of guidelines in LMICs as appreciable resources are typically needed in guideline development and adaptation (Baral et al., 2012). Technical requirements, ethical considerations, infrastructural barriers, and overburdened health systems, coupled with the lack of sufficient funding, typically prevent effective and meaningful implementation of clinical trials and other studies in LMICs to improve future antibiotic use. Consequently, it can be difficult to make conclusions regarding the applicability of guidelines that are applicable in HICs but could be a problem among LMICs (Stokes et al., 2016). To achieve success, strategies need to be adapted to overcome local resistance profiles, invest in AMS activities and diagnostics, and develop global partnerships to turn theory into practical and equitable solutions to address AMR in LMICs.

Educating key stakeholders, including prescribers and patients, about WHO AWaRe principles, including the importance of prioritizing Access over Watch antibiotics and avoiding unnecessary broad-spectrum antibiotic use, can strengthen AMS efforts at the population level (Saleem et al., 2025c). This is because patient education and counseling play crucial roles in improving antibiotic use, especially in LMICs, and enhancing healthcare outcomes (Saleem et al., 2025c). Counseling interventions often incorporate practical tools and follow-up care to support adherence. The British Thoracic Society’s 2020 Long-Term Macrolide Guidelines suggest written materials on therapy risks/benefits, whereas the ACCP 2006 Chronic Cough Guidelines advise patients on symptom relief and the self-limiting nature of viral bronchitis (Smith et al., 2020; Braman, 2006). Post-discharge instructions, including those in the SCCM 2020 Sepsis Guidelines, incorporate monitoring for secondary infections and corticosteroid-related side effects (Weiss et al., 2020). Effective counseling not only reduces unnecessary antibiotic use but also educates patients to engage in stewardship efforts, which include reducing requests and expectations for antibiotics for self-limiting viral infections for themselves or their children (Saleem et al., 2025c). In combination, these strategies connect clinical practice and community education, creating a unified front to reduce unnecessary antibiotic use and associated AMR, which is particularly important in LMICs (Mastrangelo et al., 2021).

We acknowledge that our study has limitations. We considered only English-language guidelines that were openly accessible, potentially leading to an overrepresentation of guidelines from HICs, particularly those in the European Region. There were also several countries for which we could not find guidance documents, and we encourage these countries to make their guidelines open-access and readily available to key stakeholder groups, including the general public. Similarly, our research might have missed documents depending on the search queries and engines used. However, we sought to include as many guiding documents as possible. Consequently, we believe our findings are robust and provide valuable direction for future work.

## 5 Conclusion

We found significant disparities in the availability, structure, and methodological rigor of guidelines across countries with different income levels. HICs commonly apply evidence-based frameworks such as GRADE, incorporate local AMR data, and have embedded stewardship strategies in the development of their antibiotic guidelines. This contrasts with many guidelines from LMICs, which remain generalized, outdated, and often rely on expert opinion due to limited resources, diagnostic capacity, and surveillance infrastructure. The incorporation of WHO AWaRe Book recommendations, as well as the extent to which current guidelines addressed patient education, AMR surveillance, and stewardship strategies, was also more evident in guidelines from HICs. In contrast, these components were often lacking in guidelines from LMICs, with patient education and surveillance data particularly underrepresented. The WHO AWaRe framework and Book provide a vital stepping stone, especially for LMICs; however, its successful implementation in LMICs relies on equity-led reforms. By prioritizing context-specific guidelines, increasing funding for diagnostics and stewardship, and fostering global partnerships, LMICs can transform the AWaRe framework and the associated guidance into practical and effective solutions to combat rising AMR rates. This approach will not only addresses current disparities but also help preserve the efficacy of antibiotics for future generations.

## References

[B228] African Union (2021). “African antibiotic treatment guidelines for common bacterial infections and syndromes—recommended antibiotic treatments,” in Neonatal and pediatric patients. Available online at: https://africaguidelines.onehealthtrust.org/wp-content/uploads/2021/11/Guidelines_Adults_Peds_English.pdf.

[B1] Agweyu A. Opiyo N. English M. J. B. p. (2012). Experience developing national evidence-based clinical guidelines for childhood pneumonia in a low-income setting-making the GRADE? BMC Pediatr. 12, 1–12. 10.1186/1471-2431-12-1 22208358 PMC3268095

[B2] Aisanov Z. Avdeev S. Arkhipov V. Belevskiy A. Chuchalin A. Leshchenko I. (2018). Russian guidelines for the management of COPD: algorithm of pharmacologic treatment. PMC, 183–187. 10.2147/COPD.S153770 PMC5764288 29386887

[B3] Akhloufi H. van der Sijs H. Melles D. C. van der Hoeven C. P. Vogel M. Mouton J. W. (2022). The development and implementation of a guideline-based clinical decision support system to improve empirical antibiotic prescribing. BMC Med. Inf. Decis. Mak. 22 (1), 127. 10.1186/s12911-022-01860-3 PMC9087957 35538525

[B4] Aldarhami A. (2023). Identification of novel bacteriocin against staphylococcus and bacillus species. Int. J. Health Sci. 17 (5), 15–22. PMC10484066 37692990

[B5] Alzomor O. Alhajjar S. Aljobair F. Alenizi A. Alodyani A. Alzahrani M. (2017). Management of community-acquired pneumonia in infants and children: clinical practice guidelines endorsed by the saudi pediatric infectious diseases society. Int. J. Pediatr. Adolesc. Med. 4 (4), 153–158. 10.1016/j.ijpam.2017.12.002 30805522 PMC6372484

[B84] AMPATH Antibiotic Guide (2017). Chapter 7: Central nervous system infections. South Africa: In Antibiotic guide. Available online at: https://www.ampath.co.za/storage/12/Chapter-7-Central-Nervous-System-Infections.pdf.

[B6] Antwi A. Stewart A. Crosbie M. (2020). Fighting antibiotic resistance: a narrative review of public knowledge, attitudes, and perceptions of antibiotics use. Perspect. public health 140 (6), 338–350. 10.1177/1757913920921209 32515278

[B7] ANZPID-ASAP Guideline (2016). ANZPID-ASAP guidelines for antibiotic duration and IV-Oral switch in children, newzealand. Available online at: https://starship.org.nz/search/?k=antibiotic%20guidelines&m_us=Health%20Professional.

[B10] Autore G. Bernardi L. Ghidini F. La Scola C. Berardi A. Biasucci G. (2023). Antibiotic prophylaxis for the prevention of urinary tract infections in children: guideline and recommendations from the Emilia-Romagna pediatric urinary tract infections (UTI-Ped-ER) study group,Antibiot. (Basel). 12, 1040. 10.3390/antibiotics12061040 PMC10295299 37370359

[B23] Bahria International Hospital Guideline (2023). Antibiotic guideline 2023. Lahore, Pakistan: Bahria International Hospital. Available online at: https://bahriainternationalhospital.com/5952-2/.

[B11] Balea L. B. Gulestø R. J. A. Xu H. Glasdam S. (2025). Physicians’, pharmacists’, and nurses’ education of patients about antibiotic use and antimicrobial resistance in primary care settings: a qualitative systematic literature review. Front. Antibiotics 3, 1507868. 10.3389/frabi.2024.1507868 PMC11754411 39850331

[B13] Bankar N. J. Ugemuge S. Ambad R. S. Hawale D. V. Timilsina D. R. (2022). Implementation of antimicrobial stewardship in the healthcare setting. Cureus 14 (7), e26664. 10.7759/cureus.26664 35949742 PMC9357433

[B14] Baral S. D. Wirtz A. Sifakis F. Johns B. Walker D. Beyrer C. (2012). The highest attainable standard of evidence (HASTE) for HIV/AIDS interventions: toward a public health approach to defining evidence. Public health Rep. 127 (6), 572–584. 10.1177/003335491212700607 23115382 PMC3461350

[B15] Baran A. Kwiatkowska A. Potocki L. (2023). Antibiotics and bacterial Resistance—A short story of an endless arms race. Int. J. Mol. Sci. 24 (6), 5777. 10.3390/ijms24065777 36982857 PMC10056106

[B16] Barker T. H. Dias M. Stern C. Porritt K. Wiechula R. Aromataris E. (2021). Guidelines rarely used GRADE and applied methods inconsistently: a methodological study of Australian guidelines. J. Clin. Epidemiol. 130, 125–134. 10.1016/j.jclinepi.2020.10.017 33130237

[B17] Barry E. O'Brien K. Moriarty F. Cooper J. Redmond P. Hughes C. M. (2016). PIPc study: development of indicators of potentially inappropriate prescribing in children (PIPc) in primary care using a modified Delphi technique. BMJ 6 (9), e012079. 10.1136/bmjopen-2016-012079 PMC5020844 27601499

[B18] Bartlett J. G. Breiman R. F. Mandell L. A. File T. M. (1998). Community-acquired pneumonia in adults: guidelines for management. The infectious diseases society of America. Clin. Infect. Dis. 26 (4), 811–838. 10.1086/513953 9564457

[B19] Barton M. Hawkes M. Moore D. Conly J. Nicolle L. Allen U. (2006). Guidelines for the prevention and management of community‐associated methicillin‐resistant staphylococcus aureus: a perspective for Canadian health care practitioners. Can. J. Infect. Dis. Med. Microbiol. 17, 4C–24C. 10.1155/2006/971352 PMC3555463 23365589

[B20] Bassetti M. Giacobbe D. R. Bruzzi P. Barisione E. Centanni S. Castaldo N. (2021). Clinical management of adult patients with COVID-19 outside intensive care units: guidelines from the Italian society of anti-infective therapy (SITA) and the Italian society of pulmonology (SIP). Infect. Dis. Ther. 10 (4), 1837–1885. 10.1007/s40121-021-00487-7 34328629 PMC8323092

[B21] Bayona H. Owolabi M. Feng W. Olowoyo P. Yaria J. Akinyemi R. (2017). A systematic comparison of key features of ischemic stroke prevention guidelines in low-and middle-income vs. high-income countries. J. neurological Sci. 375, 360–366. 10.1016/j.jns.2017.02.040 PMC5813247 28320168

[B22] Beneš J. Stebel R. Musil V. Krůtová M. Vejmelka J. Kohout P. (2022). Updated Czech guidelines for the treatment of patients with colitis due to Clostridioides difficile. Klin. Mikrobiol. Infekc. Lek. 28 (3), 77–94. 36791303

[B24] Bisno A. L. Gerber M. A. Gwaltney J. M. Jr Kaplan E. L. Schwartz R. H. Infectious Diseases Society of America (2002). Practice guidelines for the diagnosis and management of group A streptococcal pharyngitis. Infectious diseases society of America. Clin. Infect. Dis. 35 (2), 113–125. 10.1086/340949 12087516

[B25] Bokhari N. Akhtar S. Izhar M. Ali W. Bashir N. (2017). Pakistan chest society. Guidelines for the management of community acquired pneumonia in adults. Available online at: https://www.pakistanchestsociety.pk/wp-content/uploads/79_archives.pdf.

[B26] Boltena M. T. Woldie M. Siraneh Y. Steck V. El-Khatib Z. Morankar S. (2023). Adherence to evidence-based implementation of antimicrobial treatment guidelines among prescribers in Sub-Saharan Africa: a systematic review and meta-analysis. J. Pharm. Policy Pract. 16 (1), 137. 10.1186/s40545-023-00634-0 37936215 PMC10629154

[B27] Bonkat G. Bartoletti R. Bruyere F. Cai T. Geerlings S. Köves B. (2017). EAU guidelines on urological infections. Eur. Assoc. urology 18, 22–26.

[B29] Boon M. H. Thomson H. Shaw B. Akl E. A. Lhachimi S. K. López-Alcalde J. (2021). Challenges in applying the GRADE approach in public health guidelines and systematic reviews: a concept article from the GRADE public health group. J. Clin. Epidemiol. 135, 42–53. 10.1016/j.jclinepi.2021.01.001 33476768 PMC8352629

[B30] Borde K. Medisetty M. K. Muppala B. S. Reddy A. B. Nosina S. Dass M. S. (2022). Impact of an antimicrobial stewardship intervention on usage of antibiotics in coronavirus disease-2019 at a tertiary care teaching hospital in India. IJID Reg. 3, 15–20. 10.1016/j.ijregi.2022.02.003 35720136 PMC8820141

[B31] Boyles T. H. Brink A. Calligaro G. L. Cohen C. Dheda K. Maartens G. (2017). South African guideline for the management of community-acquired pneumonia in adults. J. Thorac. Dis. 9 (6), 1469–1502. 10.21037/jtd.2017.05.31 28740661 PMC5506119

[B32] bpacnz Guide New Zealand. (2024). Available online at: https://bpac.org.nz/antibiotics/guide.aspx.

[B262] bpacnz New Zealand (2017). The bpacnz antibiotic guide. Available online at: https://bpac.org.nz/2017/docs/abguide.pd (Accessed September 02, 2024).

[B33] Bradley J. S. Byington C. L. Shah S. S. Alverson B. Carter E. R. Harrison C. (2011). The management of community-acquired pneumonia in infants and children older than 3 months of age: clinical practice guidelines by the pediatric infectious diseases society and the infectious diseases society of America. Clin. Infect. Dis. 53 (7), e25–e76. 10.1093/cid/cir531 21880587 PMC7107838

[B34] Braman S. S. J. C. (2006). Chronic cough due to acute bronchitis: ACCP evidence-based clinical practice guidelines. Chest 129 (1), 95S-103S–103S. 10.1378/chest.129.1_suppl.95S PMC7094612 16428698

[B35] Bratzler D. W. Dellinger E. P. Olsen K. M. Perl T. M. Auwaerter P. G. Bolon M. K. (2013). Clinical practice guidelines for antimicrobial prophylaxis in surgery. Surg. Infect. 14 (1), 73–156. 10.1089/sur.2013.9999 23461695

[B36] Bredemeyer M. (2016). ACP/CDC provide guidelines on the use of antibiotics for acute respiratory tract infection. American Family Physician, 1016. Available online at: https://www.aafp.org/pubs/afp/issues/2016/1215/p1016.html.

[B37] Brinkmann I. Kibuule D. (2020). Effectiveness of antibiotic stewardship programmes in primary health care settings in developing countries. Res. Soc. Adm. Pharm. 16 (9), 1309–1313. 10.1016/j.sapharm.2019.03.008 30904409

[B38] BSMMU Guideline Bangladesh (2023). BSMMU guidelines. Available online at: https://forms.bsmmu.ac.bd/antibiotic_guideline/list.html (Accessed June 11, 2024).

[B119] BSW ICB Guidelines UK (2024). Management of infection guidance for primary Care,UK. Available online at: https://bswtogether.org.uk/medicines/wp-content/uploads/sites/3/2025/01/FULL-antibiotics-guidance-update-Dec-2024-recurrent-UTI-update.pdf (Accessed September 24, 2024).

[B39] Butler K. (2016). Manual of childhood infections. Oxford University Press.

[B40] Cao B. Huang Y. She D. Y. Cheng Q. J. Fan H. Tian X. L. (2018). Diagnosis and treatment of community‐acquired pneumonia in adults: 2016 clinical practice guidelines by the Chinese thoracic society, Chinese medical association. Clin. Respir. J. 12 (4), 1320–1360. 10.1111/crj.12674 28756639 PMC7162259

[B41] CDC Guidelines (2024). Adult antibiotic prescribing guidelines. Available online at: https://cha.com/wp-content/uploads/2018/04/Telligen-Version-CDC-Outpatient-Antibiotic-Treatment-Guidelines.pdf (Accessed June 14, 2024).

[B42] CDC (2017). Adult antibiotic prescribing guidelines. Available online at: https://www.health.ny.gov/publications/1174_8.5x11.pdf (Accessed June 13, 2024).

[B43] Charani E. Mendelson M. Pallett S. J. C. Ahmad R. Mpundu M. Mbamalu O. (2023). An analysis of existing national action plans for antimicrobial Resistance—Gaps and opportunities in strategies optimising antibiotic use in human populations. Lancet Glob. Health 11 (3), e466–e474. 10.1016/S2214-109X(23)00019-0 36739875

[B44] Chaudhuri D. Nei A. M. Rochwerg B. Balk R. A. Asehnoune K. Cadena R. (2024). 2024 focused update: guidelines on use of corticosteroids in sepsis, acute respiratory distress syndrome, and community-acquired pneumonia. Natl. Libr. Med. 52 (5), e219–e233. 10.1097/CCM.0000000000006172 38240492

[B46] CHI Guideline Ireland (2020). Antimicrobial guidelines, Ireland. Available online at: https://www.iaem.ie/wp-content/uploads/2021/02/Antimicrobial-Guidelines-2020.pdf (Accessed August 02, 2024).

[B47] Chigome A. Ramdas N. Skosana P. Cook A. Schellack N. Campbell S. (2023). A narrative review of antibiotic prescribing practices in primary care settings in South Africa and potential ways forward to reduce antimicrobial resistance. Antibiotics 12 (10), 1540. 10.3390/antibiotics12101540 37887241 PMC10604704

[B50] Chirwa M. Davies O. Castelino S. Mpenge M. Nyatsanza F. Sethi G. (2021). United Kingdom British association for sexual health and HIV national guideline for the management of epididymo-orchitis, 2020. Natl. Libr. Med. 32 (10), 884–895. 10.1177/09564624211003761 34009058

[B51] Chou Y.-H. Yang S. S. Hsieh C. H. Chang C. P. (2011). Taiwanese recommendations for antimicrobial prophylaxis in urological surgery. Urol. Sci. 22 (2), 63–69. 10.1016/s1879-5226(11)60014-6

[B52] Chow A. W. Benninger M. S. Brook I. Brozek J. L. Goldstein E. J. C. Hicks L. A. (2012). IDSA clinical practice guideline for acute bacterial rhinosinusitis in children and adults. Clin. Infect. Dis. 54 (8), e72–e112. 10.1093/cid/cir1043 22438350

[B53] CHQHospital. Children’s health Queensland paediatric antibiocard: empirical antibiotic guidelines. (2022). Available online at: https://www.childrens.health.qld.gov.au/__data/assets/pdf_file/0037/176878/Antibiocard.pdf.

[B54] Chua A. Q. Verma M. Hsu L. Y. Legido-Quigley H. (2021). An analysis of national action plans on antimicrobial resistance in southeast Asia using a governance framework approach. Lancet Regional Health–Western Pac. 7, 100084. 10.1016/j.lanwpc.2020.100084 PMC8315476 34327414

[B55] Chung W. T. G. Shafi H. Seah J. Purnima P. Patun T. Kam K. Q. (2022). National surgical antibiotic prophylaxis guideline in Singapore. Ann. Acad. Med. Singap. 51 (11), 695–711. 10.47102/annals-acadmedsg.2022273 36453217

[B56] Control N. C. F. D. National treatment guidelines for antimicrobial use in infectious diseases. (2016). Available online at: https://ncdc.mohfw.gov.in/wp-content/uploads/2024/04/File622.pdf.

[B57] Cooper L. Sneddon J. Afriyie D. K. Sefah I. A. Kurdi A. Godman B. (2020). Supporting global antimicrobial stewardship: antibiotic prophylaxis for the prevention of surgical site infection in low-and middle-income countries (LMICs): a scoping review and meta-analysis. JAC-Antimicrobial Resist. 2 (3), dlaa070. 10.1093/jacamr/dlaa070 PMC8210156 34223026

[B58] Corrêa R. d.A. Lundgren F. L. C. Pereira-Silva J. L. Frare e Silva R. L. Cardoso A. P. Lemos A. C. M. (2009). Brazilian guidelines for community-acquired pneumonia in immunocompetent adults-2009. J. Bras. Pneumol. 35, 574–601. 10.1590/s1806-37132009000600011 19618038

[B59] Cox J. A. Vlieghe E. Mendelson M. Wertheim H. Ndegwa L. Villegas M. V. (2017). Antibiotic stewardship in low-and middle-income countries: the same but different? Clin. Microbiol. Infect. 23 (11), 812–818. 10.1016/j.cmi.2017.07.010 28712667

[B60] Dadgostar P. (2019). Antimicrobial resistance: implications and costs. Infect. drug Resist. 12, 3903–3910. 10.2147/IDR.S234610 31908502 PMC6929930

[B9] Danish Health and medicine Authority Guidelines (2013). Guidelines on prescribing antibiotics, Denmark. Available online at: https://www.sundhedsstyrelsen.dk/-/media/Udgivelser/2013/Publ2013/Guidelines-on-prescribing-antibiotics.ashx.

[B61] D’Arcy N. Ashiru-Oredope D. Olaoye O. Afriyie D. Akello Z. Ankrah D. (2021). Antibiotic prescribing patterns in Ghana, Uganda, Zambia and Tanzania hospitals: results from the global point prevalence survey (G-PPS) on antimicrobial use and stewardship interventions implemented. Antibiotics 10 (9), 1122. 10.3390/antibiotics10091122 34572704 PMC8469030

[B62] Debast S. B. Bauer M. P. Kuijper E. J. European Society of Clinical Microbiology and Infectious Diseases (2014). European society of clinical microbiology and infectious diseases: update of the treatment guidance document for *Clostridium difficile* infection. Clin. Microbiol. Infect. 20, 1–26. 10.1111/1469-0691.12418 24118601

[B63] Department of Health South Africa (2023). Guidelines for the prevention and containment of antimicrobial resistance in South African hospitals. Available online at: https://knowledgehub.health.gov.za/system/files/elibdownloads/2023-04/Guidelines%2520for%2520the%2520prevention%2520and%2520containment%2520of%2520AMR%2520in%2520SA%2520hospitals.pdf (Accessed: June 16, 2024).

[B64] Desrosiers M. Evans G. A. Keith P. K. Wright E. D. Kaplan A. Bouchard J. (2011). Canadian clinical practice guidelines for acute and chronic rhinosinusitis. Allergy, Asthma and Clin. Immunol. 7, 2–38. 10.1186/1710-1492-7-2 21310056 PMC3055847

[B65] De With K. Allerberger F. Amann S. Apfalter P. Brodt H. R. Eckmanns T. (2016). Strategies to enhance rational use of antibiotics in hospital: a guideline by the German society for infectious diseases. Infection. 44, 395–439. 10.1007/s15010-016-0885-z 27066980 PMC4889644

[B66] Do P. C. Assefa Y. A. Batikawai S. M. Reid S. A. (2023). Strengthening antimicrobial resistance surveillance systems: a scoping review. BMC Infect. Dis. 23 (1), 593. 10.1186/s12879-023-08585-2 37697310 PMC10496311

[B67] DRAP Guidelines (2022). Guidelines on responsible use of antimicrobials in human health. Available online at: https://www.dra.gov.pk/wp-content/uploads/2022/02/Guidelines-Responsible-Use-of-Antimicrobials-1.pdf (Accessed: June 17, 2024).

[B68] Egypt Drug Authority Guideline (2022). National guidelines for preauthorization of restricted antimicrobials in hospitals, Egypt. Available online at: https://www.edaegypt.gov.eg/media/of1bgvnf/national-guidelines-for-preauthorization-of-restricted-ab-in-hospitals-issue-4.pdf (Accessed: December 4, 2022).

[B70] Foxlee N. D. Townell N. Heney C. McIver L. Lau C. L. (2021). Strategies used for implementing and promoting adherence to antibiotic guidelines in low-and lower-middle-income countries: a systematic review. Trop. Med. Infect. Dis. 6 (3), 166. 10.3390/tropicalmed6030166 34564550 PMC8482147

[B71] Funiciello E. Lorenzetti G. Cook A. Goelen J. Moore C. E. Campbell S. M. (2024). Identifying AWaRe indicators for appropriate antibiotic use: a narrative review. J. Antimicrob. Chemother. 79 (12), 3063–3077. 10.1093/jac/dkae370 39422368 PMC11638856

[B72] Gandra S. Alvarez-Uria G. Turner P. Joshi J. Limmathurotsakul D. van Doorn H. R. (2020). Antimicrobial resistance surveillance in Low- and middle-income countries: progress and challenges in eight South Asian and Southeast Asian countries. Clin. Microbiol. Rev. 33 (3), e00048-19. 10.1128/CMR.00048-19 32522747 PMC7289787

[B235] Georgia Pediatric Antibiotic Stewardship (2023). Georgia pediatric antibiotic stewardship guidelines guidelines. Available online at: https://www.gpas-online.org/guidelines/.

[B73] Gisbert J. P. Alcedo J. Amador J. Bujanda L. Calvet X. Castro-Fernández M. (2022). V Spanish consensus conference on *Helicobacter pylori* infection treatment. Gastroenterol. Hepatol. 45 (5), 392–417. 10.1016/j.gastrohep.2021.07.011 34629204

[B74] Gitaka J. Kamita M. Mureithi D. Ndegwa D. Masika M. Omuse G. (2020). Combating antibiotic resistance using guidelines and enhanced stewardship in Kenya: a protocol for an implementation science approach. BMJ Open 10 (3), e030823. 10.1136/bmjopen-2019-030823 PMC7170570 32234736

[B75] Godman B. Egwuenu A. Haque M. Malande O. O. Schellack N. Kumar S. (2021). Strategies to improve antimicrobial utilization with a special focus on developing countries. Life 11 (6), 528. 10.3390/life11060528 34200116 PMC8229985

[B76] Godman B. Egwuenu A. Wesangula E. Schellack N. Kalungia A. C. Tiroyakgosi C. (2022). Tackling antimicrobial resistance across Sub-Saharan Africa: current challenges and implications for the future. Expert Opin. Drug Saf. 21 (8), 1089–1111. 10.1080/14740338.2022.2106368 35876080

[B77] Gould F. K. Denning D. W. Elliott T. S. J. Foweraker J. Perry J. D. Prendergast B. D. (2012a). Guidelines for the diagnosis and antibiotic treatment of endocarditis in adults: a report of the working party of the British society for antimicrobial chemotherapy. J. Antimicrob. Chemother. 67 (2), 269–289. 10.1093/jac/dkr450 22086858

[B78] Gould F. K. Denning D. W. Elliott T. S. Foweraker J. Perry J. D. Prendergast B. D. (2012b). Guidelines for the diagnosis and antibiotic treatment of endocarditis in adults: a report of the working party of the British society for antimicrobial chemotherapy. National Library of Medicine, 269–289. Available online at: https://pubmed.ncbi.nlm.nih.gov/22086858/. 10.1093/jac/dkr450 22086858

[B69] GP Guidelines Uk (2020). Antimicrobial prescribing in dentistry – good practice Guidelines, UK. Available online at: https://www.rcseng.ac.uk/dental-faculties/fds/faculty/news/archive/antimicrobial-prescribing-guidelines/.

[B88] Greater Manchester (2024). Greater Manchester antimicrobial guidelines. Available online at: https://gmmmg.nhs.uk/wp-content/uploads/2024/02/GM-Antimicrobial-guidelines-January-2024-v15.pdf (Accessed June 20, 2024).

[B80] Guerrant R. L. Van Gilder T. Steiner T. S. Thielman N. M. Slutsker L. Tauxe R. V. (2001). Practice guidelines for the management of infectious diarrhea. Clin. Infect. Dis. 32 (3), 331–351. 10.1086/318514 11170940

[B83] Guideline Netherlands (2024). Management of community-acquired pneumonia in adults: the 2024 practice guideline, Netherland. Available online at: https://swab.nl/en/exec/file/download/300 (Accessed June 20, 2024).

[B85] Habib G. Lancellotti P. Antunes M. J. Bongiorni M. G. Casalta J. P. Zotti F. D. (2016). 2015 ESC guidelines for the management of infective endocarditis *.* 69 (1), 69. 10.1016/j.rec.2015.12.002

[B86] Haseeb A. Saleem Z. Maqadmi A. F. Allehyani R. A. Mahrous A. J. Elrggal M. E. (2023). Ongoing strategies to improve antimicrobial utilization in hospitals across the Middle East and North Africa (MENA): Findings and implications. Antibiotics 12 (5), 827. 10.3390/antibiotics12050827 37237730 PMC10215537

[B87] Hashmi M. Khan F. H. Zubairi S. Sultan S. T. Haider S. Aftab S. (2015). Developing local guidelines for management of sepsis in adults: sepsis guidelines for Pakistan (SGP).

[B89] Health G. M.O. (2020d). Provisional standard treatment guidelines for novel coronavirus infection COVID - 19 guidelines for Ghana. Available online at: https://www.moh.gov.gh/wp-content/uploads/2016/02/COVID-19-STG-JUNE-2020-1.pdf (Accessed June 22, 2024).

[B81] Health Protection Scotland (2014). Guidance on prevention and control of clostridium difficile infection (CDI) in care settings in Scotland. Available online at: https://citeseerx.ist.psu.edu/document?repid=rep1&type=pdf&doi=64eb111d42cbf505519fb9542e77b60ef9ace075 (Accessed June 16, 2024).

[B91] Health M. O. (2010). Tuvalu standard treatment guidelines. Available online at: https://www.scribd.com/document/480482038/Tuvalu-standard-treatment-guidelines.

[B93] Health M. O. (2011b). Solomon Islands guidelines for the management of major non-communicable diseases (NCDs) in primary health care. Available online at: https://aaopenplatform.accessaccelerated.org/resource-library/content/solomon-islands-guidelines-management-major-non-communicable-diseases-ncds-primary-health.

[B101] Health, M. O. (2017a). Ghana national drugs programme (GNDP) standard treatment guidelines. Available online at: https://www.moh.gov.gh/wp-content/uploads/2020/07/GHANA-STG-2017-1.pdf (Accessed June 25, 2024).

[B114] Ho C. S. Wong C. T. H. Aung T. T. Lakshminarayanan R. Mehta J. S. Rauz S. (2024). Antimicrobial resistance: a concise update. Lancet Microbe 6, 100947. 10.1016/j.lanmic.2024.07.010 39305919

[B115] Höffken G. Lorenz J. Kern W. Welte T. Bauer T. Dalhoff K. (2009). Epidemiology, diagnosis, antimicrobial therapy and management of community-acquired pneumonia and lower respiratory tract infections in adults. Guidelines of the paul-ehrlich-society for chemotherapy, the German respiratory society, the German society for infectiology and the competence network CAPNETZ Germany. Natl. Libr. Med. 63 (10), e1–e68. 10.1055/s-0029-1215037 19821215

[B116] Hospital B. (2021). Antibiotic protocol for BIRDEM general hospital. Available online at: https://file-dhaka.portal.gov.bd/uploads/c1124d76-9c11-4f4a-98ba-020b19f169d5/650/f1e/ae2/650f1eae254a3562310116.pdf (Accessed July 01, 2024).

[B117] Hunter C. R. Owen K. (2024). Can patient education initiatives in primary care increase patient knowledge of appropriate antibiotic use and decrease expectations for unnecessary antibiotic prescriptions? Fam. Pract. 42, cmae047. 10.1093/fampra/cmae047 PMC11878379 39295113

[B48] IDSC Taiwan Guidelines. (2000). Guidelines for antimicrobial therapy of urinary tract infections in Taiwan. J. Microbiol. Immunol. infection= Wei mian yu gan ran za zhi 33 (4), 271–272. 11269375

[B49] IDSC Taiwan Surgical Association (2004). Guidelines for the use of prophylactic antibiotics in surgery in Taiwan. J. Microbiol. Immunol. infection= Wei mian yu gan ran za zhi 37 (1), 71–74. 15060692

[B194] IDSP and PARN Guidelines (2019). Typhoid Management Guidelines , Pakistan. Available online at: https://www.mmidsp.com/wp-content/uploads/2019/09/Guidelines-for-Antimicrobial-Use-2.pdf.

[B118] Ibrahim W. (2016). Pneumnia guidelines development group. The diagnosis and management of community acquired pneumonia. National Clinical Guidelines. Available online at: https://www.researchgate.net/publication/312040120_Pneumnia_Guidelines_Development_Group_The_diagnosis_and_management_of_community_acquired_pneumonia_National_Clinical_Guidelines_-_Qatar_httpswwwmophgovqaclinical-guidelines (Accessed July 02, 2024).

[B120] Ihara E. Manabe N. Ohkubo H. Ogasawara N. Ogino H. Kakimoto K. (2024). Evidence-based clinical guidelines for chronic diarrhea 2023. Digestion 105 (6), 480–497. 10.1159/000541121 39197422 PMC11633876

[B121] Iskandar K. Molinier L. Hallit S. Sartelli M. Hardcastle T. C. Haque M. (2021). Surveillance of antimicrobial resistance in low- and middle-income countries: a scattered picture. Antimicrob. Resist Infect. Control 10 (1), 63. 10.1186/s13756-021-00931-w 33789754 PMC8011122

[B122] Jackson C. D. Burroughs‐Ray D. C. Summers N. A. (2020). Clinical guideline highlights for the hospitalist: 2019 American thoracic society/infectious diseases society of America update on community‐acquired pneumonia. J. Hosp. Med. 15 (12), 743–745. 10.12788/jhm.3444 32853142

[B123] Johnson S. Lavergne V. Skinner A. M. Gonzales-Luna A. J. Garey K. W. Kelly C. P. (2021). Clinical practice guideline by the infectious diseases society of America (IDSA) and society for healthcare epidemiology of America (SHEA): 2021 focused update guidelines on management of Clostridioides difficile infection in adults. Clin. Infect. Dis. 73 (5), e1029–e1044. 10.1093/cid/ciab549 34164674

[B124] Kang C.-I. Kim J. Park D. W. Kim B. N. Ha U. S. Lee S. J. (2018). Clinical practice guidelines for the antibiotic treatment of community-acquired urinary tract infections. Infect. and Chemother. 50 (1), 67–100. 10.3947/ic.2018.50.1.67 29637759 PMC5895837

[B125] Kawamatawong T. Sangasapaviriya A. Saiphoklang N. Oer-Areemitr N. Sriprasart T. Kamalaporn H. (2022). Guidelines for the management of asthma in adults: evidence and recommendations. Asian Pac. J. Allergy Immunol. 40 (1), 1–21. 10.12932/AP-210421-1118 34953479

[B126] Khan F. U. Khan F. U. Hayat K. Chang J. Saeed A. Khan Z. (2020). Knowledge, attitude and practices among consumers toward antibiotics use and antibiotic resistance in swat, khyber-pakhtunkhwa, Pakistan. Expert Rev. anti-infective Ther. 18 (9), 937–946. 10.1080/14787210.2020.1769477 32516001

[B127] Khan M. Khattak M. T. Gul A. Riaz M. Zahra F. T. (2024). A comparable risk of extensively drug-resistant typhoid fever in the pediatric cohort during the COVID-19 pandemic. Int. J. Health Sci. (Qassim) 18 (1), 24–28. 38188899 PMC10768466

[B128] Khilnani G. Zirpe K. Hadda V. Mehta Y. Madan K. Kulkarni A. (2019). Guidelines for antibiotic prescription in intensive care unit. Indian J. Crit. care Med. peer-reviewed, official Publ. Indian Soc. Crit. Care Med. 23 (Suppl. 1), S1–S63. 10.5005/jp-journals-10071-23101 PMC6734471 31516211

[B129] Kiggundu R. Lusaya E. Seni J. Waswa J. P. Kakooza F. Tjipura D. (2023). Identifying and addressing challenges to antimicrobial use surveillance in the human health sector in low-and middle-income countries: experiences and lessons learned from Tanzania and Uganda. Antimicrob. Resist. and Infect. Control 12 (1), 9. 10.1186/s13756-023-01213-3 36759872 PMC9909883

[B130] Kim Y. J. Park K. H. Park D. A. Park J. Bang B. W. Lee S. S. (2019). Guideline for the antibiotic use in acute gastroenteritis. Infect. Chemother. 51 (2), 217–243. 10.3947/ic.2019.51.2.217 31271003 PMC6609748

[B131] Klastersky J. de Naurois J. Rolston K. Rapoport B. Maschmeyer G. Aapro M. (2016). Management of febrile neutropaenia: ESMO clinical practice guidelines. Ann. Oncol. 27. v111-v118. 10.1093/annonc/mdw325 27664247

[B45] Korean Society for Chemotherapy, Korean Society of Infectious Diseases, and Korean Orthopaedic Association. (2014). Clinical guidelines for the antimicrobial treatment of bone and joint infections in Korea. Infect. and Chemother. 46 (2), 125–138. 10.3947/ic.2014.46.2.125 25024877 PMC4091374

[B132] Kulkarni A. P. Sengar M. Chinnaswamy G. Hegde A. Rodrigues C. Soman R. (2019). Indian antimicrobial prescription guidelines in critically ill immunocompromised patients. Indian J. Crit. Care Med. Peer-reviewed, Official Publ. Indian Soc. Crit. Care Med. 23 (Suppl. 1), S64–S96. 10.5005/jp-journals-10071-23102 PMC6734470 31516212

[B133] Lee M. S. Oh J. Y. Kang C. I. Kim E. S. Park S. Rhee C. K. (2018). Guideline for antibiotic use in adults with community-acquired pneumonia. Infect. and Chemother. 50 (2), 160–198. 10.3947/ic.2018.50.2.160 29968985 PMC6031596

[B134] Lee P. I. Chiu C. H. Chen P. Y. Lee C. Y. Lin T. Y. Taiwan Pediatric Working Group for Guideline on the Management of CAP in Children (2007). Guidelines for the management of community-acquired pneumonia in children. Zhonghua Minguo xiao er ke yi xue hui za Zhi. Zhonghua Minguo xiao er ke yi xue hui 48 (4), 167–180. 18265536

[B135] Lewnard J. A. Charani E. Gleason A. Hsu L. Y. Khan W. A. Karkey A. (2024). Burden of bacterial antimicrobial resistance in low-income and middle-income countries avertible by existing interventions: an evidence review and modelling analysis. Lancet 403 (10442), 2439–2454. 10.1016/S0140-6736(24)00862-6 38797180

[B136] Lipsky B. A. Senneville É. Abbas Z. G. Aragón-Sánchez J. Diggle M. Embil J. M. (2020). Guidelines on the diagnosis and treatment of foot infection in persons with diabetes (IWGDF 2019 update). Diabetes/metabolism Res. Rev. 36, e3280. 10.1002/dmrr.3280 32176444

[B137] Lubanga A. F. Bwanali A. N. Kambiri F. Harawa G. Mudenda S. Mpinganjira S. L. (2024). Tackling antimicrobial resistance in Sub-Saharan Africa: challenges and opportunities for implementing the new people-centered WHO guidelines. Expert Rev. Anti-infective Ther. 22 (6), 379–386. 10.1080/14787210.2024.2362270 38809689

[B138] Mahmudul Islam A. Raihan M. A. Ahmed K. T. Islam M. S. Nusrat N. A. Hasan M. A. (2024). Prevalence of inappropriate antibiotic doses among pediatric patients of inpatient, outpatient, and emergency care units in Bangladesh: a cross-sectional study. PLOS Glob. Public Health 4 (9), e0003657. 10.1371/journal.pgph.0003657 39255277 PMC11386430

[B140] Martin-Loeches I. Torres A. Nagavci B. Aliberti S. Antonelli M. Bassetti M. (2023). ERS/ESICM/ESCMID/ALAT guidelines for the management of severe community-acquired pneumonia. Intensive care Med. 49 (6), 615–632. 10.1007/s00134-023-07033-8 37012484 PMC10069946

[B141] Mastrangelo R. S. Santesso N. Bognanni A. Darzi A. Karam S. Piggott T. (2021). Consideration of antimicrobial resistance and contextual factors in infectious disease guidelines: a systematic survey. BMJ open 11 (7), e046097. 10.1136/bmjopen-2020-046097 PMC8327810 34330853

[B142] Mauritz M. D. von Both U. Dohna-Schwake C. Gille C. Hasan C. Huebner J. (2024). Clinical recommendations for the inpatient management of lower respiratory tract infections in children and adolescents with severe neurological impairment in Germany, 1–13. 10.1007/s00431-023-05401-6 PMC10951000 38172444

[B143] McDonald L. C. Gerding D. N. Johnson S. Bakken J. S. Carroll K. C. Coffin S. E. (2018). Clinical practice guidelines for *Clostridium difficile* infection in adults and children: 2017 update by the infectious diseases society of America (IDSA) and society for healthcare epidemiology of America (SHEA). Clin. Infect. Dis. 66 (7), 987–994. 10.1093/cid/ciy149 29562266

[B144] McTaggart S. Danchin M. Ditchfield M. Hewitt I. Kausman J. Kennedy S. (2015). KHA-CARI guideline: diagnosis and treatment of urinary tract infection in children. Nephrology 20 (2), 55–60. 10.1111/nep.12349 25307259

[B145] MHHA Guideline (2021). Guidelines for treatmentof urinary tract infections. Available online at: https://www.mi-hms.org/sites/default/files/UTI%20Guideline-6.9.21.pdf (Accessed June 01, 2024).

[B109] Ministry of Health Bangladesh (2021). Standard treatment guidelines (STG) on antibiotic use in common infectious diseases of Bangladesh. Available online at: https://amr.cdc.gov.bd/wp-content/uploads/2018/10/STG-guideline-for-antimicrobial-use-version-1.0-date-1-december21.pdf (Accessed August 03, 2024).

[B103] Ministry of Health Bhutan (2018). National-antibiotic-guideline-bhutan. Available online at: https://www.moh.gov.bt/wp-content/uploads/afd-files/2019/02/National-Antibiotic-Guideline-2018.pdf.

[B150] Ministry of Health Brunei Darussalam B. D. (2019). National best practiceguidance for good antibioticprescribing practice (GAPP). Available online at: https://moh.gov.bn/wp-content/uploads/2024/10/MOH-GAPP-Booklet.pdf.

[B151] Ministry of Health Cook Islands (2023). Guidelines for empiric and targeted antibiotic treatment, prophylaxis, dosing and allergies. Available online at: https://www.health.gov.ck/wp-content/uploads/2024/01/Cook-Islands-Handbook-Sep23_FINAL-digital.pdf.

[B155] Ministry of Health Ethiopia (2014). Standard treatment guidelines for primary hospital. Available online at: https://siapsprogram.org/wp-content/uploads/2014/12/Primary-Hospital-Final.pdf (Accessed July 05, 2024).

[B105] Ministry of Health Eswatini (2020). Eswatini standard paediatric treatment guidelines and essential medicines list of common medical conditions in the Kingdom of Eswatini.

[B94] Ministry of Health Eswatini (2012). Standard treatment gideline and EML, Eswatini. Available online at: https://www.medbox.org/document/standard-treatment-guidelines-and-essential-medicines-list-of-common-medical-conditions-in-the-kingdom-of-swaziland.

[B104] Ministry of Health Fiji (2019). Fiji antibiotic guidelines. Available online at: https://ohpl.com.fj/publication/fiji-antibiotic-guidelines-4th-edition/.

[B107] Ministry of Health Ghana (2020). Provisional Standard Treatment Guidelines for Novel Coronavirus Infection COVID - 19 Guidelines for Ghana. Ghana. Available online at: https://www.moh.gov.gh/wp-.

[B98] Ministry of Health Guyana (2015). Standard treatment guidelines for primary health Care,Guyana. Available online at: https://extranet.who.int/ncdccs/Data/GUY_D1_Guyana%20STG%202015_online.pdf.

[B147] Ministry of Health Japan (2017). Manual of antimicrobial stewardship, Japan. Available online at: https://www.mhlw.go.jp/file/06-Seisakujouhou-10900000-Kenkoukyoku/0000193504.pdf.

[B106] Ministry of Health Kenya (2020). National antimicrobial antimicrobial stewardship guidelines for health care settings in Kenya. Available online at: http://guidelines.health.go.ke:8000/media/National_Antimicrobial_Stewardship_Guidelines_for_Health_Care_Settings_in_Kenya_-_March_2020.pdf (Accessed June 29, 2024).

[B163] Ministry of Health Madagascar (2020). Guide to common infections in Madagascar. Available online at: https://www.fondation-merieux.org/en/news/the-first-guide-to-common-infections-in-madagascar-to-improve-antibiotic-use/.

[B161] Ministry of Health Malaysia (2008). National antibiotic guideline. Available online at: https://www.moh.gov.my/moh/resources/auto%20download%20images/589d720fe09a1.pdf.

[B149] Ministry of Health Malaysia (2024). National antimicrobial guideline (NAG). Available online at: https://sites.google.com/moh.gov.my/nag (Accessed July 06, 2024).

[B95] Ministry of Health Malaysia (2014). National antibiotic guideline. Available online at: https://pharmacy.moh.gov.my/sites/default/files/document-upload/national-antibiotic-guideline-2014-full-versionjun2015_1.pdf.

[B139] Ministry of Health Malawi (2015). Standard treatment guidelines (MSTG). Available online at: https://extranet.who.int/ncdccs/Data/MWI_D1_Malawi-Standard-Treatment-Guidelines-Essential-Medicines-List-2015.pdf.

[B92] Ministry of Health Namibia (2011). Namibia standard treatment guidelines. Available online at: http://www.man.com.na/files/news/1501069447namibia-standard-treatment-guidelines.pdf.

[B146] Ministry of Health Nigeria (2016). Nigeria standard treatment guidelines. Available online at: https://www.medbox.org/document/nigeria-standard-treatment-guidelines#GO (Accessed July 04, 2024).

[B97] Ministry of Health Oman (2015). National surgical antibiotic prophylaxis guideline. Available online at: https://moh.gov.om/documents/236878/0/national+surgical+antimicrobial+prophylaxis/dd57462f-2f8b-47c6-b78b-f2821ad17fc9.

[B99] Ministry of Health Oman (2016). Empirical and prophylactic use of antimicrobials national guidelines. Srilanka: MoH. Available online at: https://slmicrobiology.lk/empirical-and-prophylactic-use-of-antimicrobials-national-guidelines-sri-lanka-2024/.

[B148] Ministry of Health Rwanda (2020). COVID-19 clinical management guidelines. Available online at: https://www.rbc.gov.rw/fileadmin/user_upload/guide/Guidelines/COVID-19%20Clinical%20Managment%20guidelines.pdf.

[B111] Ministry of Health Rwanda (2022). Rwanda STG, the guideline for the use of antibiotics in Rwanda. Available online at: https://www.moh.gov.rw/index.php?eID=dumpFile&t=f&f=92525&token=65bb1585f37424835c9eeb4c9ad4747b38a80514 (Accessed May 11, 2024).

[B152] Ministry of Health Saudi Arabia (2018). National antimicrobial therapy guidelines for community and hospital acquired infections in adults. Available online at: https://saudithoracicsociety.org/wp-content/uploads/2019/11/National-Antimicrobial-Guidelines-for-Community-and-Hospital-Acquired-Infections-in-Adults.pdf.

[B156] Ministry of Health Saudi Arabia (2020). Lower respiratory tract infection protocol. Available online at: https://www.moh.gov.sa/Ministry/MediaCenter/Publications/Documents/Lower-Respiratory-Tract-Infections-Management-Protocol.pdf (Accessed July 04, 2024).

[B157] Ministry of Health Saudi Arabia (2024). UTI infection managment protocol. Available online at: https://www.moh.gov.sa/Ministry/MediaCenter/Publications/Documents/Urinary-Tract-Infection-management-protocol.pdf (Accessed July 02, 2024).

[B90] Ministry of Health Seychelles (2003). Standard treatment guidelines for Seychelles. Available online at: https://extranet.who.int/ncdccs/Data/SYC_D1_Standard%20Treatment%20Guidlines-Seychelles.pdf.

[B96] Ministry of Health Somalia (2015). Somalia national treatment guidelines in line with the essential package of health services. Available online at: https://moh.gov.so/en/wp-content/uploads/2020/07/Primary-Health-Unit-I-Somali-Treatment-Guidelines-2015.pdf.

[B100] Ministry of Health Srilanka (2016). Empirical and prophylactic use of antimicrobials national guidelines,Srilanak. Available online at: https://slmicrobiology.lk/empirical-and-prophylactic-use-of-antimicrobials-national-guidelines-sri-lanka-2024/.

[B153] Ministry of Health Tanzania (2020). Policy guideline for implementing antimicrobial stewardship. Available online at: https://www.moh.go.tz/storage/app/uploads/public/657/6b5/58e/6576b558e32b1485010391.pdf (Accessed June 28, 2024).

[B102] Ministry of Health Tanzania (2017). Standard treatment guideline and NEML, Tanzania mainland. Available online at: https://hssrc.tamisemi.go.tz/storage/app/uploads/public/5ab/e9b/b21/5abe9bb216267130384889.pdf (Accessed June 26, 2024).

[B110] Ministry of Health Tanzania (2021). Standard treatment guideline and EML for Tanzania mainland. Available online at: https://medicine.st-andrews.ac.uk/igh/wp-content/uploads/sites/44/2022/01/STG-NEMLIT-2021.pdf.

[B154] Ministry of Health UAE (2022). National guidelines on the empiric antibiotic treatment of intra-abdominal infections, UAE guideline. Available online at: https://mohap.gov.ae/documents/20117/626089/61c6912c-df03-4bc6-93c0-e2837bd093c2/1208b629-2260-45c4-7bc0-5d9fb6f576e4.

[B108] Ministry of Health Uganda (2020). National guidelines for antimicrobial consumption and use surveillance in human health published by the ministry of health, republic of Uganda. Available online at: https://cphl.go.ug/web/sites/default/files/2024-10/National%20Guidelines%20for%20AMCU%20in%20HH%2017.06.2020.pdf.

[B112] Ministry of Health Uganda (2023). Uganda clinical guidelines. Available online at: https://library.health.go.ug/sites/default/files/resources/Uganda%20Clinical%20Guidelines%202023.pdf (Accessed June 29, 2024).

[B162] Ministry of Health Zambia (2014). Essential newborn care Guidelines,Zambia. Available online at: https://www.afro.who.int/sites/default/files/2019-06/ESSENTIAL%20NEWBORN%20CARE%20GUIDELINES%202014.pdf.

[B181] Ministry of Health Zimbabwe (2015). 7th essential medicines list and standard treatment guidelines for Zimbabwe. Available online at: https://platform.who.int/docs/default-source/mca-documents/policy-documents/essential-medicines-and-equipment/zwe-ch-43-01-emd-2015-eng-edliz-2015.pdf.

[B158] Miyashita N. Matsushima T. Oka M. Japanese Respiratory Society (2006). The JRS guidelines for the management of community-acquired pneumonia in adults: an update and new recommendations. Intern. Med. 45 (7), 419–428. 10.2169/internalmedicine.45.1691 16679695

[B159] MMIDSP Guideline (2022). Typhoid management guidelines, Pakistan. Available online at: https://www.mmidsp.com/wp-content/uploads/2023/03/Typhoid-Management-Guideline-2022-Jun-22.pdf.

[B160] Moghnieh R. Yared Sakr N. Kanj S. S. Musharrafieh U. Husni R. Jradeh M. (2014). The Lebanese society for infectious diseases and clinical microbiology (LSIDCM) guidelines for adult community-acquired pneumonia (CAP) in Lebanon. J. Med. Liban. 103 (1006), 40–47. 10.12816/0002626 24684125

[B164] Moja L. Zanichelli V. Mertz D. Gandra S. Cappello B. Cooke G. S. (2024). WHO's essential medicines and AWaRe: recommendations on first-and second-choice antibiotics for empiric treatment of clinical infections. Clin. Microbiol. Infect. 30, S1–S51. 10.1016/j.cmi.2024.02.003 38342438

[B165] MSF Clinical guidelines. (2024) Clinical guidelines - diagnosis and treatment manual, Spain. Available online at: https://medicalguidelines.msf.org/sites/default/files/pdf/guideline-170-en.pdf.

[B166] Murray C. J. Ikuta K. S. Sharara F. Swetschinski L. Robles Aguilar G. Gray A. (2022). Global burden of bacterial antimicrobial resistance in 2019: a systematic analysis. Lancet 399 (10325), 629–655. 10.1016/s0140-6736(21)02724-0 35065702 PMC8841637

[B167] Mustafa Z. U. Salman M. Khan A. H. Harun S. N. Meyer J. C. Godman B. (2024). Antimicrobial use among hospitalized neonates and children; findings and implications from a comprehensive point prevalence survey among general tertiary hospitals in Pakistan. Infect. Drug Resist. 17, 5411–5428. 10.2147/IDR.S491454 39664724 PMC11631696

[B168] Mzumara G. W. Mambiya M. Iroh Tam P.-Y. (2023). Protocols, policies and practices for antimicrobial stewardship in hospitalized patients in least-developed and low-income countries: a systematic review. Antimicrob. Resist. and Infect. Control 12 (1), 131. 10.1186/s13756-023-01335-8 37993964 PMC10666353

[B169] Nagassar R. P. (2020). Recommendations for antibiotic prescriptions for upper respiratory symptoms in children in Trinidad and Tobago: grade-adolopment approach. Available online at: https://www.caribbeanmedicaljournal.org/2020/05/09/recommendations-for-antibiotic-prescriptions-for-upper-respiratory-symptoms-in-children-in-trinidad-and-tobago-grade-adolopmen.

[B170] NAGCOM Philippine Guideline (2017). National Antibiotic Guideline 2017, Philippine. Available online at: http://www.pidsphil.org/home/wp-content/uploads/2018/09/National-Antibiotic-Guidlines-Dr.-Carmina-Delos-Reyes.pdf.

[B171] Nair M. Tripathi S. Mazumdar S. Mahajan R. Harshana A. Pereira A. (2019). Without antibiotics, I cannot treat: a qualitative study of antibiotic use in paschim bardhaman district of West Bengal, India. PloS one 14 (6), e0219002. 10.1371/journal.pone.0219002 31247028 PMC6597109

[B172] Nascimento-Carvalho C. M. S.-M. Souza-Marques H. H. (2004). Recommendation of the Brazilian society of pediatrics for antibiotic therapy in children and adolescents with community-acquired pneumonia. 15, 380–387. 10.1590/s1020-49892004000600003 15272984

[B173] Nathwani D. Varghese D. Stephens J. Ansari W. Martin S. Charbonneau C. (2019). Value of hospital antimicrobial stewardship programs [ASPs]: a systematic review. Antimicrob. Resist. and Infect. Control 8, 35–13. 10.1186/s13756-019-0471-0 30805182 PMC6373132

[B113] National department of Health Papua New Guinea (2019). Papua New Guinea national guidelines for HIV care and treatment. Available online at: https://www.aidsdatahub.org/sites/default/files/resource/papua-new-guinea-national-guidelines-hiv-care-and-treatment-2019.pdf.

[B175] NDOH/WHO Papua New Guinea (2012). Standard treatment guidelines for adults in Papua New Guinea. Available online at: https://extranet.who.int/ncdccs/Data/PNG_D1_Standard-Treatment-Guidelines-for-Common-Illness-of-Adults-in-PNG.pdf.

[B176] NHRA Bahrain (2022). Treatment guidelines and pathways. Available online at: https://www.nhra.bh/MediaHandler/GenericHandler/documents/Announcements/COVID-19/General/Bahrain%20Treatment%20Protocol%20(V12.0)%20Jan%202022%20(English).pdf.

[B177] NICE CAP Guideline (2019). Nice guideline pneumonia (community acquired): antimicrobial prescribing,UK. Available online at: https://www.nice.org.uk/guidance/ng138.

[B178] NICE HAP Guideline (2019). Pneumonia (hospital-acquired): antimicrobial prescribing, UK. Available online at: https://www.nice.org.uk/guidance/ng139/resources/pneumonia-hospitalacquired-antimicrobial-prescribing-pdf-66141727749061?utm (Accessed: July 08, 2024).

[B179] NICE Guidelines (2023). Pneumonia in adults: diagnosis and management NICE guideline. Available online at: https://www.nice.org.uk/guidance/cg191.

[B180] Nishimura R. A. Carabello B. A. Faxon D. P. Freed M. D. Lytle B. W. O'Gara P. T. (2008). ACC/AHA 2008 guideline update on valvular heart disease: focused update on infective endocarditis: a report of the American college of cardiology/american heart association task force on practice guidelines: endorsed by the society of cardiovascular anesthesiologists, society for cardiovascular angiography and interventions, and society of thoracic surgeons. ACC/AHA 2008 Guidel. update valvular heart Dis. Focus. update Infect. endocarditis a Rep. Am. Coll. Cardiology/American Heart Assoc. Task Force Pract. Guidel. endorsed by Soc. Cardiovasc. Anesthesiol. Soc. Cardiovasc. Angiogr. Interventions, Soc. Thorac. Surg. 118 (8), 887–896. 10.1161/CIRCULATIONAHA.108.190377

[B182] Norris A. H. Shrestha N. K. Allison G. M. Keller S. C. Bhavan K. P. Zurlo J. J. (2019). 2018 infectious diseases society of America clinical practice guideline for the management of outpatient parenteral antimicrobial therapy. Clin. Infect. Dis. 68 (1), e1–e35. 10.1093/cid/ciy745 30423035

[B183] Nsubuga P. White M. E. Thacker S. B. Anderson M. A. Blount S. B. Broome C. V. (2011). Public health surveillance: a tool for targeting and monitoring interventions. 21250345

[B184] Ohemu G. P. (2022). Starved of ACTION: a critical look at the antimicrobial resistance action plans of African countries. ACS Infect. Dis. 8 (8), 1377–1380. 10.1021/acsinfecdis.2c00303 35916786

[B185] Ohge H. Mayumi T. Haji S. Kitagawa Y. Kobayashi M. Kobayashi M. (2021). The Japan society for surgical infection: guidelines for the prevention, detection, and management of gastroenterological surgical site infection. Surg. Today 51, 1–31. 10.1007/s00595-020-02181-6 33320283 PMC7788056

[B186] Okolie O. J. Igwe U. Ismail S. U. Ighodalo U. L. Adukwu E. C. (2023). Systematic review of surveillance systems for AMR in Africa. J. Antimicrob. Chemother. 78 (1), 31–51. 10.1093/jac/dkac342 PMC9780554 36227707

[B187] Oladele R. Ettu A. O. Medugu N. Habib A. Egbagbe E. Osinaike T. (2023). Antibiotic guidelines for critically ill patients in Nigeria. West Afr. J. Med. 40 (9), 962–972. 37768104

[B188] Olayemi E. Asare E. V. Benneh‐Akwasi Kuma A. A. (2017). Guidelines in lower‐middle income countries. Br. J. Haematol. 177 (6), 846–854. 10.1111/bjh.14583 28295193

[B190] Owolabi M. Olowoyo P. Miranda J. J. Akinyemi R. Feng W. Yaria J. (2016). Gaps in hypertension guidelines in low-and middle-income *versus* high-income countries: a systematic review. Hypertension 68 (6), 1328–1337. 10.1161/HYPERTENSIONAHA.116.08290 27698059 PMC5159303

[B191] Owolabi M. O. Yaria J. O. Daivadanam M. Makanjuola A. I. Parker G. Oldenburg B. (2018). Gaps in guidelines for the management of diabetes in low-and middle-income *versus* high-income Countries—A systematic review. Diabetes Care 41 (5), 1097–1105. 10.2337/dc17-1795 29678866 PMC5911785

[B192] Paioni P. Aebi C. Bielicki J. Buettcher M. Crisinel P. A. Kahlert C. R. (2022). Swiss recommendations on perioperative antimicrobial prophylaxis in children. Natl. Libr. Med. 152 (3738), w30230. 10.4414/smw.2022.w30230 36201236

[B227] Pakistan Chest Society Guideline. (2020). Guidelines for managment of COPD, Pakistan. Available online at: https://www.pakistanchestsociety.pk/wp-content/uploads/2020/09/COPD-Update.pdf.

[B193] Park J. B. Kario K. Wang J. G. (2015). Systolic hypertension: an increasing clinical challenge in Asia. Hypertens. Res. 38 (4), 227–236. 10.1038/hr.2014.169 25503845 PMC4396396

[B195] Pediatrics A. A. o. (2001). Clinical practice guideline: management of sinusitis. Pediatrics 108 (3), 798–808. 10.1542/peds.108.3.798 11533355

[B196] Pelucchi C. Grigoryan L. Galeone C. Esposito S. Huovinen P. Little P. (2012). Guideline for the management of acute sore throat. Clin. Microbiol. Infect. 18. 1–28. 10.1111/j.1469-0691.2012.03766.x 22432746

[B197] Peters A.-C. Larsson D. G. J. Laxminarayan R. Munthe C. (2024). Barriers and pathways to environmental surveillance of antibiotic resistance in middle-and low-income settings: a qualitative exploratory key expert study. Glob. Health Action 17 (1), 2343318. 10.1080/16549716.2024.2343318 38813982 PMC11141306

[B198] PIOA Guidelines Samoa (2017). Antibiotic use guidelines. Available online at: https://www.pioa.net/wp-content/uploads/2019/08/PIOA-Antibiotic-guidelines.pdf.

[B199] Polverino E. Goeminne P. C. McDonnell M. J. Aliberti S. Marshall S. E. Loebinger M. R. (2017). European respiratory society guidelines for the management of adult bronchiectasis. Eur. Respir. J. 50 (3), 1700629. 10.1183/13993003.00629-2017 28889110

[B200] PPUKM Malaysia Guidelien (2012). PPUKM anti-infective guideline, Malaysia. Available online at: https://hctm.ukm.my/farmasi/wp-content/uploads/2020/08/Anti-Infective-Guideline-2012-2MB.pdf.

[B201] PSMID UTI Guidelines Philippine (2015). “Philippine clinical practice guidelines on the diagnosis and management of urinary tract infections in adults 2015 update: part 2 asymptomatic bacteriuria in adults, recurrent urinary tract infection,” in Complicated urinary tract infection. Available online at: https://www.psmid.org/diagnosis-and-management-of-urinary-tract-infections-in-adults-2015-update-part-2/(Accessed July 16, 2024).

[B203] Ramdas N. Meyer J. C. Schellack N. Godman B. Turawa E. Campbell S. M. (2025). Knowledge, attitudes, motivations, expectations, and systemic factors regarding antimicrobial use amongst community members seeking care at the primary healthcare level: a scoping review. Antibiotics 14 (1), 78. 10.3390/antibiotics14010078 39858364 PMC11761248

[B204] Randel A. (2018). Infectious diarrhea: IDSA updates guidelines for diagnosis and management. Am. Fam. Physician 97 (10), 676–677. 29763277

[B28] Red Book (2021). Report of the committee on infectious diseases. Available online at: https://publications.aap.org/aapbooks/book/663/Red-Book-2021-Report-of-the-Committee-on?autologincheck=redirected (Accessed June 05, 2024).

[B205] Riddle M. S. DuPont H. L. Connor B. A. (2016). ACG clinical guideline: diagnosis, treatment, and prevention of acute diarrheal infections in adults. Official J. Am. Coll. Gastroenterology| ACG 111 (5), 602–622. 10.1038/ajg.2016.126 27068718

[B206] Roberts K. B. Pediatrics M. J. S.C.o.Q.I. Subcommittee on Urinary Tract Infection (2011). Urinary tract infection: clinical practice guideline for the diagnosis and management of the initial UTI in febrile infants and children 2 to 24 months. Elk Grove Village, IL, USA: American Academy of Pediatrics, 595–610. 10.1542/peds.2011-1330 21873693

[B207] Rose M. Barker M. Liese J. Adams O. Ankermann T. Baumann U. (2020). “Guidelines for the management of community acquired pneumonia in children and adolescents (pediatric community acquired pneumonia, pCAP)-issued under the responsibility of the German Society for Pediatric Infectious Diseases (DGPI) and the German Society for Pediatric Pulmonology (GPP). Germany: National Library of Medicine. 74(8) 515–544. 10.1055/a-1139-5132 32823360

[B208] Rosenfeld R. M. Andes D. Bhattacharyya N. Cheung D. Eisenberg S. Ganiats T. G. (2007). Clinical practice guideline: adult sinusitis. Otolaryngol. Head. Neck Surg. 137 (3), S1–S31. 10.1016/j.otohns.2007.06.726 17761281

[B209] Rosenfeld R. M. Piccirillo J. F. Chandrasekhar S. S. Brook I. Ashok Kumar K. Kramper M. (2015). Clinical practice guideline (update): adult sinusitis. Otolaryngology–Head Neck Surg. 152 (2_Suppl. l), S1-S39–S39. 10.1177/0194599815572097 25832968

[B210] Rosenfeld R. M. Shin J. J. Schwartz S. R. Coggins R. Gagnon L. Hackell J. M. (2016). Clinical practice guideline: otitis media with effusion (update). Otolaryngology–Head Neck Surg. 154 (1_Suppl. l), S1–S41. 10.1177/0194599815623467 26832942

[B82] Sanford Guideline (2024). Pneumonia ventilator associated 2024. Available online at: https://web.sanfordguide.com/en/sanford-guide-online/disease-clinical-condition/pneumonia-ventilator-associated (Accessed June 09, 2024).

[B79] SAP Guideline Australia (2021). Surgical antimicrobial prophylaxis guidelines. Available online at: https://www.sahealth.sa.gov.au/wps/wcm/connect/public+content/sa+health+internet/clinical+resources/clinical+programs+and+practice+guidelines/medicines+and+drugs/antimicrobial+guidelines/antimicrobial+guidelines (Accessed June 18, 2024).

[B211] SAASP (2014). Pocket guide to antibiotic prescribing for adults in South Africa. Available online at: https://sahivsoc.org/Files/Guide%20to%20Antibiotice%20prescribing%20for%20adults%20in%20SA_2014%20(Oct%202014).pdf.

[B212] Sackett D. L. Rosenberg W. M. Gray J. M. Haynes R. B. Richardson W. S. (1996). Evidence based medicine: what it is and what it isn't. British Medical Journal Publishing Group, 71–72. 10.1136/bmj.312.7023.71 PMC2349778 8555924

[B213] Sakeena M. Bennett A. A. McLachlan A. J. (2018). Enhancing pharmacists’ role in developing countries to overcome the challenge of antimicrobial resistance: a narrative review. Antimicrob. Resist. and Infect. Control 7, 63–11. 10.1186/s13756-018-0351-z 29744044 PMC5930749

[B214] Saleem Z. Godman B. Azhar F. Kalungia A. C. Fadare J. Opanga S. (2022). Progress on the national action plan of Pakistan on antimicrobial resistance (AMR): a narrative review and the implications. Expert Rev. anti-infective Ther. 20 (1), 71–93. 10.1080/14787210.2021.1935238 34038294

[B215] Saleem Z. Mekonnen B. A. Orubu E. S. Islam M. A. Nguyen T. T. P. Ubaka C. M. (2025c). Current access, availability and use of antibiotics in primary care among key low-and middle-income countries and the policy implications. Expert Rev. Anti-infective Ther., 1–42. 10.1080/14787210.2025.2477198 40110804

[B216] Saleem Z. Moore C. E. Kalungia A. C. Schellack N. Ogunleye O. Chigome A. (2025a). Status and implications of the knowledge, attitudes and practices towards AWaRe antibiotic use, resistance, and stewardship among low-and middle-income countries. JAC-Antimicrobial Resist. 7, dlaf033. 10.1093/jacamr/dlaf033 PMC11934068 40134815

[B217] Saleem Z. Sheikh S. Godman B. Haseeb A. Afzal S. Qamar M. U. (2025b). Increasing the use of the WHO AWaRe system in antibiotic surveillance and stewardship programs in Low- and middle-income countries. JAC Antimicrob. Resist. 7 (2), dlaf031. 10.1093/jacamr/dlaf031 40110554 PMC11919820

[B218] Sartorius B. Gray A. P. Weaver N. D. Aguilar G. R. Swetschinski L. R. Ikuta K. S. (2024). The burden of bacterial antimicrobial resistance in the WHO African region in 2019: a cross-country systematic analysis. Lancet Glob. Health 12 (2), e201–e216. 10.1016/S2214-109X(23)00539-9 38134946 PMC10805005

[B219] Schoffelen T. Papan C. Carrara E. Eljaaly K. Paul M. Keuleyan E. (2024). European society of clinical microbiology and infectious diseases guidelines for antimicrobial stewardship in emergency departments (endorsed by European association of hospital pharmacists). Clin. Microbiol. Infect. 30 (11), 1384–1407. 10.1016/j.cmi.2024.05.014 39029872

[B220] Senneville É. Albalawi Z. van Asten S. A. Abbas Z. G. Allison G. Aragón-Sánchez J. (2024). IWGDF/IDSA guidelines on the diagnosis and treatment of diabetes‐related foot infections (IWGDF/IDSA 2023). Diabetes/metabolism Res. Rev. 40 (3), e3687. 10.1002/dmrr.3687 37779323

[B221] Sharland M. Gandra S. Huttner B. Moja L. Pulcini C. Zeng M. (2019). Encouraging AWaRe-ness and discouraging inappropriate antibiotic Use—The new 2019 essential medicines list becomes a global antibiotic stewardship tool. Lancet Infect. Dis. 19 (12), 1278–1280. 10.1016/S1473-3099(19)30532-8 31782385

[B222] SHCH/HMC (2016). Clinical practice Guidelines,Standard antibiotic treatment guidelines. Available online at: https://niph.org.kh/niph/uploads/library/pdf/GL061_CPG_AB_SHCH_V2_0.pdf (Accessed July 22, 2024).

[B223] Shi Y. Huang Y. Zhang T. T. Cao B. Wang H. Zhuo C. (2019). Chinese guidelines for the diagnosis and treatment of hospital-acquired pneumonia and ventilator-associated pneumonia in adults (2018 edition). J. Thorac. Dis. 11, 2581–2616. 10.21037/jtd.2019.06.09 31372297 PMC6626807

[B224] Siachalinga L. Mufwambi W. Lee I.-H. (2022). Impact of antimicrobial stewardship interventions to improve antibiotic prescribing for hospital inpatients in Africa: a systematic review and meta-analysis. J. Hosp. Infect. 129, 124–143. 10.1016/j.jhin.2022.07.031 35970382

[B8] SLMA Guidelines (2014). SLMA guidelines on the use of antimicrobial Agents,Srilanka. Available online at: https://slma.lk/wp-content/uploads/2014/12/Guidelines-on-the-use-of-antimicrobial-agents.pdf.

[B226] Smith D. Du Rand I. A. Addy C. Collyns T. Hart S. Mitchelmore P. (2020). British thoracic society guideline for the use of long-term macrolides in adults with respiratory disease. BMJ open Respir. Res. 7 (1), e000489. 10.1136/bmjresp-2019-000489 PMC7204798 32332022

[B225] Slovenian Guidelines (2014). Slovenian guidelines on antibiotic prophylaxis application in neurosurgical operations. Available online at: https://vestnik.szd.si/index.php/ZdravVest/article/view/1017/915 (Accessed September 10, 2024).

[B229] Spellberg B. Gilbert D. N. Baym M. Bearman G. Boyles T. Casadevall A. (2025). Sustainable solutions to the continuous threat of antimicrobial resistance. Health Aff. Sch. 3 (2), qxaf012. 10.1093/haschl/qxaf012 39916975 PMC11798182

[B230] SPIM. *Antibiotika Tsara* (2018). Antibiotic guide Madagascar. Available online at: https://smartbiotic.ai/product/antibiotika-tsara-madagaskar/.

[B231] Spindler C. Strålin K. Eriksson L. Hjerdt-Goscinski G. Holmberg H. Lidman C. (2012). Swedish guidelines on the management of community-acquired pneumonia in immunocompetent adults—Swedish society of infectious diseases 2012. Natl. Libr. Med. 44 (12), 885–902. 10.3109/00365548.2012.700120 22830356

[B232] Stein R. Dogan H. S. Hoebeke P. Kočvara R. Nijman R. J. M. Radmayr C. (2015). Urinary tract infections in children: EAU/ESPU guidelines. Eur. Urol. 67 (3), 546–558. 10.1016/j.eururo.2014.11.007 25477258

[B233] Steinberg E. Greenfield S. Wolman D. M. Mancher M. Graham R. (2011). Clinical practice guidelines we can trust. national academies press. 24983061

[B234] Stevens D. L. Bisno A. L. Chambers H. F. Dellinger E. P. Goldstein E. J. C. Gorbach S. L. (2014). Practice guidelines for the diagnosis and management of skin and soft tissue infections: 2014 update by the infectious diseases society of America. Clin. Infect. Dis. 59 (2), e10–e52. 10.1093/cid/ciu444 24973422

[B202] STGs Papua New Guinea (2016). Standard treatment for common illnesses of children in Papua New Guinea. Available online at: https://pngpaediatricsociety.org/wp-content/uploads/2016/11/PNG-Standard-Treatment-Book-10th-edition-2016.pdf.

[B246] STGs Solomon (2017). Standard treatment manual for children. Available online at: https://www.unicef.org/pacificislands/media/851/file/Standard-Treatment-Manual.pdf.

[B236] Stokes T. Shaw E. J. Camosso-Stefinovic J. Imamura M. Kanguru L. Hussein J. (2016). Barriers and enablers to guideline implementation strategies to improve obstetric care practice in low-and middle-income countries: a systematic review of qualitative evidence. Implement. Sci. 11, 144–10. 10.1186/s13012-016-0508-1 27770807 PMC5075167

[B237] Sulis G. Daniels B. Kwan A. Gandra S. Daftary A. Das J. (2020). Antibiotic overuse in the primary health care setting: a secondary data analysis of standardised patient studies from India, China and Kenya. BMJ Glob. Health 5 (9), e003393. 10.1136/bmjgh-2020-003393 PMC7493125 32938614

[B238] Sulis G. Sayood S. Gandra S. (2022). Antimicrobial resistance in low-and middle-income countries: current status and future directions. Expert Rev. anti-infective Ther. 20 (2), 147–160. 10.1080/14787210.2021.1951705 34225545

[B239] Sy C. L. Chen P. Y. Cheng C. W. Huang L. J. Wang C. H. Chang T. H. (2022). Recommendations and guidelines for the treatment of infections due to multidrug resistant organisms. Recomm. Guidel. Treat. Infect. due multidrug Resist. organisms,Taiwan 55 (3), 359–386. 10.1016/j.jmii.2022.02.001 35370082

[B240] Tacconelli E. Mazzaferri F. de Smet A. M. Bragantini D. Eggimann P. Huttner B. D. (2019). ESCMID-EUCIC clinical guidelines on decolonization of multidrug-resistant Gram-negative bacteria carriers. Clin. Microbiol. Infect. 25 (7), 807–817. 10.1016/j.cmi.2019.01.005 30708122

[B241] Tamma P. D. Heil E. L. Justo J. A. Mathers A. J. Satlin M. J. Bonomo R. A. (2024). Infectious diseases society of America 2024 guidance on the treatment of antimicrobial-resistant gram-negative infections. Clin. Infect. Dis., ciae403. 10.1093/cid/ciae403 39108079

[B242] Tawfick M. M. Adulall A. K. El-Kholy A. A. Manakhly A. R. E. (2023). Mutation-based fluoroquinolone resistance in carbapenem-resistant Acinetobacter baumannii and *Escherichia coli* isolates causing catheter-related bloodstream infections. Int. J. Health Sci. (Qassim) 17 (3), 18–25. PMC10155246 37151743

[B243] Therapeutic Guidelines Australia (2024). Therapeutic guidelines antibiotic prescribing in primary care. Available online at: https://ccmsfiles.tg.org.au/s3/PDFs/GPSummary_v15.pdf ([Accessed July 24, 2024).

[B244] Thi T. V. L. Pham E. C. Dang-Nguyen D.-T. (2024). Evaluation of children's antibiotics use for outpatient pneumonia treatment in Vietnam. Braz. J. Infect. Dis. 28 (4), 103839. 38996808 10.1016/j.bjid.2024.103839 PMC11321292

[B245] Torres A. Niederman M. S. Chastre J. Ewig S. Fernandez-Vandellos P. Hanberger H. (2017). International ERS/ESICM/ESCMID/ALAT guidelines for the management of hospital-acquired pneumonia and ventilator-associated pneumonia: guidelines for the management of hospital-acquired pneumonia (HAP)/ventilator-associated pneumonia (VAP) of the European Respiratory Society (ERS), European Society of Intensive Care Medicine (ESICM), European Society of Clinical Microbiology and Infectious Diseases (ESCMID) and Asociación Latinoamericana del Tórax (ALAT). Available online at: https://pubmed.ncbi.nlm.nih.gov/28890434/(Accessed July 24, 2024). 10.1183/13993003.00582-2017 28890434

[B174] United Nations (2024). Political declaration of the high-level meeting on antimicrobial resistance. Available online at: https://www.un.org/pga/wp-content/uploads/sites/108/2024/09/FINAL-Text-AMR-to-PGA.pdf (Accessed July 05, 2024).

[B247] Valadares H. N. G. (2016). Antibiotic guidelines. Available online at: https://extranet.who.int/uhcpartnership/sites/default/files/reports/Timor-Leste-Antibiotic-Guidelines.pdf (Accessed August 11, 2024).

[B248] Van de Beek D. Cabellos C. Dzupova O. Esposito S. Klein M. Kloek A. T. (2016). ESCMID guideline: diagnosis and treatment of acute bacterial meningitis. Clin. Microbiol. Infect. 22, S37–S62. 10.1016/j.cmi.2016.01.007 27062097

[B249] Versporten A. Zarb P. Caniaux I. Gros M. F. Drapier N. Miller M. (2018). Antimicrobial consumption and resistance in adult hospital inpatients in 53 countries: results of an internet-based global point prevalence survey. Lancet Glob. Health 6 (6), e619–e629. 10.1016/S2214-109X(18)30186-4 29681513

[B250] Wagenlehner F. M. Dittmar F. (2022). Re: global burden of bacterial antimicrobial resistance in 2019: a systematic analysis. Eur. Urol. 82 (6), 658. 10.1016/j.eururo.2022.08.023 36068104

[B251] Wan X. Miao R. Zhang N. Huang W. Wu Z. Wang H. (2025). Global burden of antimicrobial resistance in lower respiratory infections in 2021: a systematic analysis. Int. J. Antimicrob. Agents 65 (2), 107431. 10.1016/j.ijantimicag.2024.107431 39734053

[B252] Wang W. Yu S. Zhou X. Wang L. He X. Zhou H. (2022). Antibiotic prescribing patterns at children’s outpatient departments of primary care institutions in southwest China. BMC Prim. Care 23 (1), 269. 10.1186/s12875-022-01875-9 36289470 PMC9607730

[B253] Weiss S. L. Peters M. J. Alhazzani W. Agus M. S. D. Flori H. R. Inwald D. P. (2020). Surviving sepsis campaign international guidelines for the management of septic shock and sepsis-associated organ dysfunction in children. Intensive Care Med. 46, 10–67. 10.1007/s00134-019-05878-6 32030529 PMC7095013

[B254] WHO (2016). Global action plan on antimicrobial resistance. Available online at: https://www.who.int/publications/i/item/9789241509763 (Accessed August 12, 2024).

[B255] WHO (2019). North okkalapa general and teaching hospital antibiotic guidelines. Myanmar. Available online at: https://medbox.org/pdf/62fb4f0042d0cad2d90d2495.

[B189] WHO (2022). The WHO AWaRe (access, watch, reserve) antibiotic book. Available online at: https://www.who.int/publications/i/item/9789240062382.

[B256] Willemsen A. Reid S. Assefa Y. (2022). A review of national action plans on antimicrobial resistance: strengths and weaknesses. Antimicrob. Resist. and Infect. Control 11 (1), 90. 10.1186/s13756-022-01130-x 35739564 PMC9229779

[B257] Windfuhr J. P. Toepfner N. Steffen G. Waldfahrer F. Berner R. (2016). Clinical practice guideline: Tonsillitis I. Diagnostics and nonsurgical management. Eur. Archives Oto-Rhino-Laryngology 273, 973–987. 10.1007/s00405-015-3872-6 PMC7087627 26755048

[B258] Woolf S. H. Grol R. Hutchinson A. Eccles M. Grimshaw J. (1999). Clinical guidelines: potential benefits, limitations, and harms of clinical guidelines. Bmj 318 (7182), 527–530. 10.1136/bmj.318.7182.527 10024268 PMC1114973

[B12] World Bank (2025). Washington, DC, United States: World Bank Country and Lending Groups. Available online at: https://datahelpdesk.worldbank.org/knowledgebase/articles/906519-world-bank-country-and-lending-groups (Accessed June 03, 2024).

[B259] Yang L. Weston C. Cude C. Kincl L. (2020). Evaluating Oregon's occupational public health surveillance system based on the CDC updated guidelines. Am. J. Industrial Med. 63 (8), 713–725. 10.1002/ajim.23139 PMC7383881 32483871

[B260] Yoon Y. K. Park C. S. Kim J. W. Hwang K. Lee S. Y. Kim T. H. (2017). Guidelines for the antibiotic use in adults with acute upper respiratory tract infections. Infect. and Chemother. 49 (4), 326–352. 10.3947/ic.2017.49.4.326 29299900 PMC5754344

[B261] Young Peart S. T C. R. Abel C. (2018). Management guidelines for urinary tract infections in Jamaican children 2018. Available online at: https://kidneykidsja.com/wp-content/uploads/Jamaican-2018-guidelines-for-UTI-investigation-in-kids.pdf.

