# Supplementary material for: Global variation in antibiotic prescribing guidelines and the implications for decreasing AMR in the future
Source: Front Pharmacol. 2025 Aug 7;16:1600787. doi: 10.3389/fphar.2025.1600787 (PMC12368364; doi:10.3389/fphar.2025.1600787)
Supplement: Supplementary file 1 [file Supplementaryfile1.docx]

**Supplementary Table 1 (S1): Number of guidelines included in study across difference Regions and countries in them**

| **WHO Regions** | **Countries** | **Guidelines** |
| --- | --- | --- |
| **African Region** | South Africa, Seychelles, Namibia, Eswatini, Ghana, Kenya, Nigeria, Tanzania, Zambia Madagascar, Ethiopia, Malawi, Rwanda, Uganda, Zimbabwe | 29 |
| **Eastern Mediterranean Region** | Bahrain, Oman, Qatar, Saudi Arabia, UAE, Egypt, Lebanon, Pakistan, Somalia | 19 |
| **European Region** | Czech Republic, Denmark, Georgia, Germany, Ireland, Italy, Netherland, Russia, Spain, Slovenia, Sweden, Switzerland, Trinidad and Tobago, UK (including Scotland) | 31 |
| **Region of the Americas** | Brazil, Canada, Guyana, Jamaica, USA | 35 |
| **South-East Asia Region** | Bangladesh, Bhutan, India, Maldives, Myanmar, Sri Lanka, Thailand, Timor Leste | 13 |
| **Western Pacific Region** | Australia, Brunei Darussalam, Cambodia, China, Cook Islands, Fiji, Japan, Korea, Malaysia, New Zealand, Papua New Guinea, Philippine, Samoa, Singapore, Solomon, Taiwan, Tuvalu | 41 |

**Supplementary Table (S2): Global Variation**

**a) European Antimicrobial Prescribing Guidelines**

| Global Guidelines | Guideline Abbreviation | Date of Publication | Methodology | Prepared By | Scope | Resistance Patterns Addressed | Key Recommendations Linked to Resistance | Implementation Strategies / Compliance and Monitoring / Outcome Measures | Patient Education and Counseling | Reference |
| --- | --- | --- | --- | --- | --- | --- | --- | --- | --- | --- |
| Europe | EAUGUIDELINES ON UI | 2024 | Evidence-based review, expert consensus | EAU | International | Addresses resistance in E. coli (ESBL-producing strains), Klebsiella pneumoniae, and Pseudomonas aeruginosa | Recommends narrow-spectrum antibiotics (e.g., nitrofurantoin) for uncomplicated UTIs; reserves fluoroquinolones for severe cases | Includes diagnostic algorithms, stewardship tools, and compliance audits | Emphasizes patient education on adherence and prevention | [1] |
|  | ESCMID ED Stewardship Guidelines | 2024 | Evidence-based review, expert consensus | ESCMID, EAHP | International | Addresses resistance in Staphylococcus aureus (MRSA), ESBL-producing Enterobacteriaceae, and Pseudomonas aeruginosa | Recommendations s: Empiric therapy: Targeted antibiotics based on local resistance data. De-escalation: Adjust therapy post-culture. Stewardship: ED-specific protocols to reduce misuse | Include compliance and antibiotic stewardship strategies | Emphasizes patient counseling on adherence and antibiotic risks | [2] |
|  | ERS/ESICM/ESCMID/ALAT CAP-G | 2023 | GRADE | ERS/ ESICM/ESCMID/ ALAT | National | Addresses resistance in Streptococcus pneumoniae (penicillin/cephalosporin resistance), Legionella pneumophila, and Gram-negative pathogens (e.g., Pseudomonas aeruginosa) | Recommends Empiric therapy: β-lactam + macrolide or fluoroquinolone.  MRSA/Pseudomonas risk: Add vancomycin/linezolid or anti-pseudomonal agents. Stewardship: De-escalate based on culture results | Includes severity scoring (e.g., PSI, CURB-65), rapid diagnostics (e.g., PCR), and stewardship protocols | Emphasizes vaccination (pneumococcal, influenza) and smoking cessation | [3] |
|  | ESCMID-EUCIC Decolonization Guidelines | 2019 | Evidence-based review, expert consensus | ESCMID, EUCIC | International | Targets carbapenem-resistant Enterobacteriaceae (CRE), Acinetobacter baumannii (CRAB), and Pseudomonas aeruginosa (CRPA | Recommends:  - Selective digestive decontamination (e.g., colistin/gentamicin) for CRE.  - Chlorhexidine baths for CRAB/CRPA.  - Avoid routine decolonization in low-risk settings | Includes screening protocols, compliance monitoring, and outcome tracking (e.g., reduction in hospital outbreaks) | Emphasizes hygiene practices to prevent transmission | [4] |
|  | ERS guidelines for AB | 2017 | GRADE. | ERS | International | Antimicrobial resistance in P. aeruginosa, H. influenzae, S. pneumoniae, S. aureus (including MRSA) | Use antibiotics for exacerbations based on sputum culture results. Avoid prolonged or unnecessary antibiotics to reduce resistance risk. Tailor therapy to local resistance patterns (e.g., P. aeruginosa resistance). Consider inhaled antibiotics for chronic P. aeruginosa infection | Emphasizes on regular sputum surveillance to tailor antibiotic therapy and manage chronic infections. recommend regular follow-ups to ensure treatment adherence. Outcome measures include exacerbation frequency, quality of life, lung function, and pathogen clearance. | educating patients about their condition, treatment options, and the importance of adherence to therapy. guidance on airway clearance techniques, recognizing symptoms of exacerbations, and when to seek medical help. | [5] |
|  | ERS-HAP/VAP Guideline | 2017 | GRADE | ERS/ESICM/ESCMID/ALAT | International | MDR pathogens: MRSA, P. aeruginosa, Acinetobacter baumannii, ESBL-producing Enterobacterales, carbapenem-resistant organisms (CRO) | Empiric broad-spectrum therapy (e.g., anti-MRSA + antipseudomonal β-lactam) in high-risk/MDR settings. De-escalate based on culture/susceptibility results. Limit duration (7–8 days for most cases).Avoid unnecessary antibiotics in colonization vs. true infection. | Antibiotic stewardship programs in ICUs. Surveillance of local ICU resistance trends. Routine microbiologic testing (e.g., bronchoalveolar lavage cultures). Bundle compliance monitoring (e.g., timely antibiotics, ventilator care). | Counsel on ICU infection control (e.g., hand hygiene, ventilator care). Educate families on VAP prevention strategies. | [6] |
|  | ESCMID Meningitis Guidelines | 2016 | GRADE | Microbiology ESCMID | International | Addresses resistance in Streptococcus pneumoniae (penicillin/cephalosporin resistance) and Neisseria meningitidis | Recommends empiric ceftriaxone/cefotaxime + vancomycin for suspected penicillin-resistant S. pneumoniae; adjunct dexamethasone for adults | Includes rapid diagnostics (e.g., PCR), stewardship programs, and compliance audits | Emphasizes vaccination (pneumococcal, meningococcal) and early symptom recognition | [7] |
|  | ESMO-Febrile neutropenia | 2016 | GRADE | ESMO | International | Prioritizes empiric coverage for MDR pathogens (e.g., P. aeruginosa, ESBL-producing E. coli), with de-escalation to reduce resistance risk. | Empiric broad-spectrum therapy: Piperacillin-tazobactam or carbapenems for high-risk FN. De-escalate based on culture/susceptibility results. Avoid prolonged antibiotics unless persistent neutropenia or confirmed infection. Tailor therapy to local resistance patterns (e.g., ESBL prevalence). | emphasize the importance of prompt action to potential infections, patient education, and clear written instructions for outpatients. highlight the importance of early detection using tools like the MASCC score to predict complications and guide treatment decisions | Outpatients should monitor symptoms and have clear instructions on when to seek help. Effective written policies and clear emergency protocols are crucial for rapid response to FN | [8] |
|  | Blue Book | 2016 | Evidence-based review, expert consensus | OUP, ESPID | International | Antimicrobial resistance in common pediatric pathogens (e.g., S. pneumoniae, H. influenzae, S. aureus, E. coli) | Use narrow-spectrum agents. Avoid unnecessary antibiotics for viral infections. Tailor therapy to local resistance patterns (e.g., MRSA prevalence). Promote vaccination to reduce antibiotic use | Diagnostic tools (e.g., rapid strep tests, urine cultures). Antibiotic stewardship programs in pediatric clinics. Track resistance rates via surveillance systems. Provide education on guideline adherence. | Educate caregivers on antibiotic risks and self-care for viral infections. Counsel on adherence to prescribed regimens. Promote vaccination to prevent infections. | [9] |
|  | ESC-Endocarditis-G | 2015 | Evidence based expert consensus | ESC-EACTS & EANM | International | Antimicrobial resistance in S. aureus, Enterococcus spp., Streptococcus spp., HACEK organisms | Empiric therapy: Vancomycin + gentamicin + ceftriaxone for suspected IE, adjusted based on culture results. Targeted therapy: Use narrow-spectrum agents when susceptibility is confirmed. Avoid prolonged prophylaxis to reduce resistance risk. Tailor therapy to local resistance patterns (e.g., MRSA prevalence) | Multidisciplinary IE teams (e.g., cardiologists, infectious disease specialists). Antibiotic stewardship programs to optimize IE treatment and prophylaxis. Surveillance of local resistance patterns (e.g., MRSA prevalence). Monitoring adherence to IE prophylaxis guidelines | Educate patients on IE prevention (e.g., oral hygiene, skin care). Counsel on antibiotic adherence and risks of resistance. Provide post-discharge support for long-term management | [10] |
|  | EAU/ESPU-UTI- | 2015 | literature review, evidence based | By EAU/ESPU | International | Outdated Guideline: Published in 2015, this guideline does not reflect current resistance trends (e.g., rising ESBL-producing E. coli). Refer to updated AAP/IDSA guidelines or local antibiograms for modern recommendations | First-line therapy: Oral amoxicillin-clavulanate or cephalosporins (e.g., cefixime). Avoid TMP-SMX or ampicillin as empiric therapy in regions with high resistance. Tailor therapy based on local susceptibility patterns. Limit antibiotic use to confirmed UTIs (via urinalysis/culture) | Standardize diagnostic criteria (e.g., urinalysis, urine culture). Antibiotic stewardship programs to optimize empiric therapy. Track resistance rates via pediatric antibiograms. Educate providers on guideline adherence. | Counsel caregivers on completing antibiotic courses. Educate on signs of UTI recurrence. Communicate risks of antibiotic overuse (e.g., resistance, side effects) | [11] |
|  | EAU UTI Guidelines | 2014 | GRADE | EAU | International | Addresses resistance in E. coli (carbapenem-resistant), Pseudomonas aeruginosa, and Staphylococcus saprophyticus | . Empiric therapy: Fosfomycin for uncomplicated UTIs Complicated UTIs: Use beta-lactam/beta-lactamase inhibitors (e.g., ceftazidime-avibactam) for ESBL producers. Prophylaxis: Low-dose antibiotics or non-antibiotic alternatives (e.g., D-mannose) for recurrent UTIs | Integration into national stewardship frameworks, clinician education, and resistance surveillance | Counseling on hydration, hygiene, and antibiotic stewardship | [12] |
|  | ESCMID-ST Guideline | 2012 | GRADE | ESCMID/ ESCMID STG Group | International | Antimicrobial resistance in S. pyogenes (Group A Streptococcus, GAS) | Avoid antibiotics for viral pharyngitis. Use first-line agents (e.g., penicillin V) for confirmed GAS pharyngitis. Limit duration (10 days for penicillin; 5 days for macrolides). Avoid macrolides unless penicillin-allergic due to resistance risks | Diagnostic tools (e.g., rapid strep tests, clinical scoring systems). Antibiotic stewardship programs to reduce overprescribing. Track resistance rates via surveillance systems. | Educate patients on the self-limiting nature of viral sore throat. Counsel on symptomatic management (e.g., analgesics, hydration). Communicate risks of unnecessary antibiotics (e.g., resistance, side effects). | [13] |

b) WHO Region

| **Income Level** | Country Name | Guideline Abbreviation | Date of Publication | Methodology | Prepared By | Scope | Resistance Patterns Addressed | Key Recommendations Linked to Resistance | Implementation Strategies / Compliance and Monitoring / Outcome Measures | Patient Education and Counseling | Reference |
| --- | --- | --- | --- | --- | --- | --- | --- | --- | --- | --- | --- |
| **African Region** | | | | | | | | | | | |
| **High Income** | Seychelles | Seychelles STGs | 2003 | Combined local medical practice experience with relevant international standards and guidelines. | MOH | National | Addresses resistance in Streptococcus pneumoniae and E. coli | Recommends first-line antibiotics and discourages empiric broad-spectrum use | Integrated with stewardship programs and formulary management | Public education on rational antibiotic use | [14] |
| **Upper Middle income** | Namibia | Namibia STG | 2011 | Evidence based Guideline. | MOH & SC | National | Addresses resistance in Streptococcus pneumoniae, Klebsiella pneumoniae, and MRSA | Recommends first-line antibiotics (e.g., amoxicillin) for common infections; restricts broad-spectrum use to severe cases | Includes healthcare worker training, compliance audits, and stewardship programs | Promotes community education on antibiotic adherence | [15] |
| **Lower Middle income** | Eswatini | Eswatini STG-EML | 2021 | Evidence-based recommendations aligned with WHO Essential Medicines List | Ministry of Health, Eswatini | National | Addresses resistance in Mycobacterium tuberculosis and Gram-negative bacteria | Prioritizes essential antibiotics (e.g., amoxicillin); discourages empiric broad-spectrum use | Integrated with stewardship programs and formulary restrictions | Public education on adherence and prevention | [16] |
|  |  | Eswatini  STG-EML | 2012 | Evidence based Guidelines. | Ministry of Health | National | Targets resistance in Mycobacterium tuberculosis (MDR-TB), Staphylococcus aureus (MRSA), and E. coli | . Prioritize essential antibiotics (e.g., amoxicillin) for community-acquired infections .Reserve carbapenems and fluoroquinolones for confirmed resistance . Discourage antibiotic use for viral infections | Stewardship training, formulary restrictions, and compliance audits | Community campaigns on adherence and hygiene | [17] |
|  | Ghana | Ghana COVID-19 STG | 2020 | Evidence-based review, expert consensus, local AMR | MOH | National | Not explicitly addressed (focus on viral infection, not antimicrobial resistance) | Recommends antiviral therapies (e.g., remdesivir) and supportive care based on symptom severity | Integrated with Ghana’s COVID-19 Emergency Response Plan and surveillance systems | Includes public awareness campaigns and guidance on isolation protocols | [18] |
|  |  | GNDP STG | 2017 | based on evidence quality RCTs, clinical studies, expert opinions | MOH | National | Addresses resistance in common infections (e.g., urinary tract infections: ciprofloxacin resistance 62.3%, ESBL-producing E. coli 50%) | Recommends updates to reflect rising resistance (e.g., reduced use of ciprofloxacin for UTIs | Integrated with National Health Insurance Scheme; includes antimicrobial stewardship initiatives under the 2017–2021 National Action Plan | Limited explicit guidance; awareness campaigns via AMR platform | [19] |
|  | Kenya | Kenya AMS Guidelines | 2020 | Based on AMS spectrum, development and implementation of systems, interventions | MOH | National | targets resistance in pathogens like MRSA, ESBL-producing Enterobacteriaceae, and multidrug-resistant Gram-negative bacteria | Recommends antibiotic restriction policies (e.g., preauthorization for broad-spectrum agents), de-escalation, and stewardship programs | Includes compliance audits, healthcare worker training, and stepwise implementation strategies | Limited explicit guidance; focuses on clinician adherence | [20] |
|  |  | Kenya AMS Protocol | 2020 | Multiregional Stepwise Stewardship Intervention: 18-Month ASP Adaptation | KNRF | Local | Targets resistance in Salmonella spp., Shigella spp., and multidrug-resistant Gram-negative bacteria | Emphasizes guideline adoption, stewardship training, and continuous feedback loops to optimize antibiotic use | Stepwise implementation strategy with pre/post-intervention audits, outcome monitoring, and policy dissemination | Limited explicit guidance; focuses on clinician training | [21] |
|  |  | Kenya AMS Guidelines | 2012 | GRADE | MMS | National | Addresses penicillin-resistant Streptococcus pneumoniae and Haemophilus influenzae | Recommends oral amoxicillin as first-line therapy; limits injectable antibiotics to severe cases | Includes training programs for healthcare workers and simplified diagnostic algorithms | Emphasizes caregiver education on adherence and symptom recognition | [22] |
|  | Nigeria | Nigeria ICU Guidelines | 2023 | based on evidence based published data and local prospective antibiograms | Experts Committee | National | Addresses resistance in Acinetobacter baumannii, ESBL-producing Enterobacteriaceae, and MRSA | Recommends empiric broad-spectrum antibiotics (e.g., carbapenems) for sepsis, followed by de-escalation based on culture results | Includes stewardship programs, compliance audits, and outcome monitorin | Limited explicit guidance; focuses on clinician adherence | [23] |
|  |  | Nigeria STG 2016 | 2016 | Evidence Based Guideline. | MOH | National | Addresses resistance in common pathogens (e.g., Plasmodium falciparum, Salmonella typhi, Staphylococcus aureus) | Recommends first-line antibiotics (e.g., amoxicillin, cotrimoxazole) for common infections; restricts broad-spectrum agents (e.g., cephalosporins) to severe case | Integrated with national health programs, healthcare worker training, and stewardship initiatives | Includes community education on adherence and prevention | [24] |
|  | Tanzania | STG/NEMLIT | 2021 | NEMLIT new edition aligns with WHO recommendations | MOH /CD | National | Addresses antimicrobial resistance (AMR) through WHO AWaRe antibiotic classification | Prioritize Access group antibiotics (e.g., amoxicillin) for common infections. Reserve Watch and Reserve antibiotics (e.g., cephalosporins, carbapenems) for confirmed resistance or severe cases . | Stewardship programs and compliance audits.Monitoring via DHIS2 and hospital-selected antimicrobial lists 15  - Training for healthcare workers | Community awareness campaigns on rational antibiotic use and hygiene | [25] |
|  |  | AMS Policy Guideline | 2020 | Evidence-based recommendations aligned with WHO AMR Action Plan and local data | MOH | National | Targets resistance in E. coli, Klebsiella pneumoniae, and Staphylococcus aureus (MRSA) | Establish AMS committees in hospitals. Rational prescribing: Prioritize WHO Access antibiotics (e.g., amoxicillin) and restrict Watch/Reserve group antibiotics . Implement AMR monitoring systems in sentinel sites | Training for healthcare workers on AMS principles. Compliance audits and feedback mechanisms. Integration with DHIS2 for AMR data tracking | Community awareness campaigns on AMR and rational antibiotic use | [26] |
|  |  | STG-NEMLIT | 2017 | Evidence-based recommendations, expert consensus, and local AMR data | MOH | National | Targets resistance in Salmonella typhi, E. coli, and Plasmodium falciparum | Recommends first-line antibiotics (e.g., cotrimoxazole) and reserves cephalosporins for severe cases | Includes stewardship training and compliance monitoring | Community awareness campaigns on antibiotic misuse | [27] |
|  | Zambia | ENC Guidelines 2014 | 2014 | Evidence based Guidelines. | MINISTRY OF COMMUNITY DEVELOPMENT MOTHER AND CHILD HEALTH | National | Addresses resistance in neonatal sepsis pathogens (e.g., Streptococcus agalactiae) | Recommends narrow-spectrum antibiotics (e.g., penicillin/gentamicin) for sepsis; limits prophylactic use | Integrated with maternal health programs and neonatal training | Emphasizes breastfeeding and hygiene practices | [28] |
| **Low Income** | Ethiopia | Ethiopia PH STGs | 2014 | Evidence-based recommendations aligned with WHO EML and local disease burden | MOH | National | Addresses chloroquine-resistant Plasmodium falciparum, penicillin-resistant Streptococcus pneumoniae, and MDR-TB | 1. Artemisinin-based Combination Therapies (ACTs) for malaria; 2. Amoxicillin as first-line for bacterial pneumonia; 3. DOTS for TB to curb MDR-TB | Integrated with healthcare worker training, formulary restrictions, and stewardship programs | Community education on antibiotic adherence and hygiene practices | [29] |
|  | Malawi | MSTG 5th Edition | 2015  5th Edition | Evidence Based Guideline. | MOH | National | Addresses resistance in Streptococcus pneumoniae, E. coli, and Klebsiella pneumoniae | Recommends amoxicillin as first-line for bacterial infections; restricts broad-spectrum use to severe cases | Includes compliance audits (79.6% adherence overall) and healthcare worker training | Limited explicit guidance; focuses on clinician adherence | [30] |
|  | Madagascar | Malagasy Infection Guide | 2020 | Evidence-based recommendations, expert consensus, and alignment with local disease burden | Ministry of Public Health | National | Addresses resistance risks from empiric antibiotic use for viral fevers and bacterial infections | Recommends rational antibiotic use, proper dosing, and duration; prioritizes diagnostics for bacterial cases | Integrated with RESAMAD laboratory network for bacteriology support and training | Emphasizes clinician training and diagnostic stewardship | [31] |
|  |  | Antibiotika Tsara | 2018 | Digital tool combining local resistance data and stewardship algorithms | Society of Infectious Pathology Madagascar (SPIM) | Local | Targets resistance in Salmonella, E. coli, and Staphylococcus aureus | Provides real-time antibiotic recommendations based on local resistance patterns and patient-specific factors | Integrated with TSARA surveillance tool and RESAMAD laboratory network | Includes clinician alerts and adherence monitoring | [32] |
|  | Rwanda | Rwanda Antibiotic Guidelines | 2022 | based on available evidence and literature review. | MOH | National | Targets resistance in Staphylococcus aureus (MRSA), E. coli (ESBL-producing strains), and Klebsiella pneumoniae | Recommends narrow-spectrum antibiotics (e.g., amoxicillin) for common infections; restricts carbapenems and fluoroquinolones to severe cases | Includes stewardship training, point-prevalence surveys, and alignment with the National AMR Surveillance System | Limited explicit guidance; focuses on provider education | [33] |
|  |  | Rwanda COVID-19 Guidelines | 2020 | Evidence-based, expert consensus, alignment with WHO guidelines | RBC | National | Not explicitly addressed (focus on viral infection, not antimicrobial resistance) | Recommends supportive care, oxygen therapy, and antiviral therapies (e.g., remdesivir) based on severity | Integrated with Rwanda’s COVID-19 response plan, surveillance systems, and training program | Includes public awareness campaigns and guidance on isolation protocols | [34] |
|  | South Africa | SA Hospital AMR Guidelines | 2023 | Policy framework aligned with WHO and national AMR action plans | NDH | National | Addresses multidrug-resistant Acinetobacter baumannii, VRE, and CRE | Recommends stewardship teams, formulary restrictions, and surveillance of resistance patterns | Includes compliance audits, staff training, and outbreak response protocols | Emphasizes hand hygiene and isolation protocols | [35] |
|  |  | Africa CDC Guidelines | 2021 | Evidence-based recommendations, expert consensus, and alignment with local AMR data | Africa CDC, OHT | International | Addresses resistance in Streptococcus pneumoniae, E. coli, and Klebsiella pneumoniae | Addresses resistance in Streptococcus pneumoniae, E. coli, and Klebsiella pneumoniae | Recommends narrow-spectrum antibiotics (e.g., amoxicillin) for empiric therapy; limits broad-spectrum use | Emphasizes clinician training | [36] |
|  |  | AMPATH Guidelines | 2017 | Laboratory-driven recommendations, local antibiograms, diagnostic protocols | AMPATH | National | Focuses on ESBL-producing Enterobacteriaceae, MRSA, and Pseudomonas aeruginosa | Recommends tailored therapy based on culture results; promotes rapid diagnostics (e.g., PCR, MALDI-TOF) | Integrated with laboratory networks and stewardship programs | . Limited explicit guidance; focuses on clinician-laboratory collaboration | [37] |
|  |  | SA CAP Guidelines | 2017 | Evidence-based recommendations, expert consensus, and CRB-65 severity scoring | SATC | National | Addresses penicillin-resistant Streptococcus pneumoniae and macrolide-resistant Mycoplasma pneumoniae | Recommends amoxicillin for mild-moderate CAP; macrolides for atypical pathogens or penicillin allergy | Includes severity stratification and microbiological testing protocols | Promotes smoking cessation and vaccination | [38] |
|  |  | SAASP Pocket Guide | 2014 | Evidence-based recommendations, expert consensus, and local resistance data | (SAASP) | National | Targets resistance in Klebsiella pneumoniae (carbapenem-resistant), MRSA, and ESBL-producing Enterobac | Recommends empiric therapy (e.g., ceftriaxone for sepsis) with de-escalation based on culture results | Includes diagnostic algorithms and stewardship tools (e.g., CRB-65 score for pneumonia) | Limited explicit guidance | [39] |
|  | Uganda | Uganda Clinical Guidelines | 2023 | Evidence-based recommendations aligned with WHO standards, expert consensus, local disease burden | MOH | National | Addresses resistance in Mycobacterium tuberculosis, Plasmodium falciparum (malaria), and antimicrobial resistance (AMR) | . Rational antibiotic use: Prioritize narrow-spectrum antibiotics for common infections AMR containment: Align with Uganda’s National AMR Action Plan (2018–2023) for stewardship and surveillance 413. TB/HIV management: Use standardized regimens to prevent drug resistance | - Stewardship programs integrated with Uganda’s AMR National Action Plan Training for healthcare workers on guideline adherence. Monitoring via Essential Medicines List compliance | Community education on hygiene, adherence to treatment, and AMR awareness | [40] |
|  |  | Uganda Guideline | 2020 | Ugandan approach combines WHO guidance, national policies, and local innovation. | MOH | National | Not explicitly addressed (focus on viral infection) | Recommends antibiotics only for confirmed bacterial co-infections (e.g., azithromycin for atypical pneumonia) | Mentions roles and responsibilities of stakeholders, including the Ministry of Health and health facilities, for effective antimicrobial surveillance.  Also includes a monitoring and evaluation plan with key performance indicators to track progress and ensure adherence to guidelines. | emphasize the importance of promoting public awareness, training, and education to optimize the use of antimicrobials and improve infection prevention and control. | [41] |
|  | Zimbabwe | EDLIZ Guideline | 2015 | Evidence based Guidelines. | NMTPAC/MOH | National | Addresses resistance in Mycobacterium tuberculosis and MRSA | Recommends first-line antibiotics (e.g., amoxicillin) for community-acquired infections | Includes healthcare worker training and formulary management | Community education on rational antibiotic use | [42] |
| Eastern Mediterranean Region | | | | | | | | | | | |
| High Income | Bahrain | TG & Pathways | 2022 | Evidence based Guidelines. | NHRA | National | Not explicitly addressed (focus on viral infection) | Recommends antivirals (e.g., remdesivir), corticosteroids, and oxygen therapy; limits antibiotics to confirmed bacterial co-infections | Integrated with Bahrain’s COVID-19 response plan, surveillance systems, and healthcare worker training | Includes public awareness campaigns on treatment adherence and prevention | [43] |
|  | Oman | Oman AMS Guidelines | 2016 | Evidence-based recommendations, expert consensus, alignment with intl. standards | MOH | National | Addresses resistance in common pathogens (e.g., E. coli, Klebsiella pneumoniae, Staphylococcus aureus) | Recommends first-line antibiotics (e.g., amoxicillin, cotrimoxazole) for common infections; discourages overuse of broad-spectrum agents | Includes stewardship programs, compliance audits, and outcome monitoring | Emphasizes clinician training and patient education on adherence | [44] |
|  |  | Oman SAP Guidelines | 2015 | National Antimicrobial Subcommittee Surgical team practice based | MOH | National | Addresses resistance risks from prolonged antibiotic use and inappropriate selection (e.g., overuse of broad-spectrum agents) | Recommends cefuroxime as first-line prophylaxis; limits duration to ≤24 hours; discourages post-surgical antibiotic use | includes audits, compliance monitoring, and training for surgical teams | Limited explicit guidance; focuses on clinician adherence | [45] |
|  | Qatar | Qatar CAP Guidelines | 2016 | Evidence-based recommendations aligned with WHO standards | MOH | National | National coverage for CAP diagnosis and management; aligns with Gulf region practices and IDSA/ATS guidelines | Targets resistance in Streptococcus pneumoniae, Haemophilus influenzae, and atypical pathogens | Integrated with national healthcare training, compliance audits, and MOPH monitoring | Community awareness campaigns on early symptom recognition | [46] |
|  | Saudi Arabia | UTI infection protocol | 2023 | Evidence-based recommendations aligned with IDSA, WHO and local AMR | MOH | Adults & Children | Targets resistance in E. coli (ESBL-producing), Klebsiella pneumoniae, and Pseudomonas aeruginosa | . First-line therapy: Nitrofurantoin or fosfomycin for uncomplicated UTIs ..Reserve fluoroquinolones/cephalosporins for complicated cases or confirmed resistanceMandate urine culture for recurrent/severe infections | Integrated stewardship programs, clinician training, and prescription audits | Emphasizes hydration, hygiene, and adherence to prescribed regimens | [47] |
|  |  | Lower RTI protocol | 2020 | Evidence based ,expert consensus | MOH | National | Antimicrobial resistance in Streptococcus pneumoniae, Haemophilus influenzae, Klebsiella pneumoniae, Mycoplasma pneumoniae | First-line therapy: Amoxicillin-clavulanate or respiratory fluoroquinolones (e.g., levofloxacin) for bacterial LRTI.. Avoid unnecessary antibiotics for viral LRTI (e.g., viral bronchitis). Limit duration | Antibiotic stewardship programs in hospitals and clinics.Surveillance of resistance patterns via Saudi surveillance networks Diagnostic tools (e.g., sputum cultures, CRP testing). Provider training on guideline adherence. | Educate patients on completing antibiotic courses. Counsel on avoiding antibiotic misuse for viral infections. Communicate risks of resistance and side effects | [48] |
|  |  | NAT-G for CAP -HAPAdults | 2018 | based on evidence-based selection and local epidemiology of antimicrobial resistance. | MOH | National | Addresses Pseudomonas aeruginosa (e.g., carbapenem resistance), ESBL-producing Enterobacteriaceae, MRSA, and MDR pathogens | Recommends narrow-spectrum antibiotics (e.g., amoxicillin-clavulanate) for mild infections; restricts carbapenems/vancomycin to severe cases or confirmed resistance | Prospective audit, formulary restriction, education, guidelines, combination therapy, and IV to PO conversion.: Focus on dose optimization, drug monitoring, and resistance surveillance. Metrics and surveillance systems for empirical therapy and antibiotic consumption | Not mentioned. | [49] |
|  |  | Saudi CAP in Adults | 2017 | Evidence-based recommendations, expert consensus, literature review | SPIDS | National | Addresses resistance in Streptococcus pneumoniae (e.g., penicillin resistance) and Mycoplasma pneumoniae | Recommends amoxicillin as first-line therapy; limits macrolides to atypical pneumonia or penicillin allergy | Includes severity stratification, microbiological testing protocols, and stewardship alignment with national ABS programs | It emphasizes educating caregivers about the signs and symptoms of pneumonia, the importance of adherence to prescribed treatments, and when to seek further medical attention. This helps ensure proper management and reduces the risk of complications. | [50] |
|  | UAE | UAE IAI Guidelines | 2022 | Evidence-based recommendations aligned with local AMR data | N-ASC | National | Targets ESBL-producing Enterobacteriaceae, Pseudomonas aeruginosa, and anaerobe | Recommends piperacillin-tazobactam or carbapenems for high-risk cases; emphasizes early de-escalation | Integrated with stewardship programs and surgical site infection protocols | Education on post-surgical care and adherence | [51] |
| Lower Middle Income | Egypt | Egypt Preauthorization Guidelines | 2022 | Based onEgyptian Hospital Antimicrobial Data | EDA/NARC | National | Targets multidrug-resistant Gram-negative pathogens (e.g., ESBL-producing Enterobacteriaceae, carbapenem-resistant Acinetobacter baumannii) | Requires preauthorization for 14 restricted antimicrobials (e.g., carbapenems, polymyxin B) to curb misuse; promotes stewardship programs | Includes formulary restrictions, compliance audits, and 24-hour infectious disease expert consultation | Limited explicit guidance; focuses on clinician adherence | [52] |
|  | Lebanon | LSIDCM CAP Guidelines | 2014 | Evidence-based recommendations adapted to Lebanese local AMR | LSIDCM | National | Addresses resistance in Streptococcus pneumoniae and Haemophilus influenzae | Recommends amoxicillin/clavulanate as first-line; reserves fluoroquinolones for high-risk cases | Training for healthcare providers and public awareness campaign | Education on adherence and symptom monitoring | [53] |
|  | Pakistan | Bahria Hospital Guidelines | 2023 | Hospital formulary and stewardship-driven recommendations | BIH | Local | Focuses on resistance in Staphylococcus epidermidis and Enterococcus | Restricts fluoroquinolones and third-generation cephalosporins | Stewardship rounds and antibiotic timeout policies | Counseling on completing antibiotic courses | [54] |
|  |  | DRAP Guidelines | 2022 | Regulatory framework aligned with WHO Global Action Plan on AMR | DRAP | National | Targets resistance due to over-the-counter sales and misuse | Mandates prescription-only access to antibiotics; discourages empiric use | Regulatory enforcement, public campaigns, and pharmacy audits | Public awareness on AMR and antibiotic misuse | [55] |
|  |  | Typhoid Management Guidelines | 2022 | Evidence-based recommendations, local resistance surveillance, and expert consensus | MMIDSP | National | Focuses on extensively drug-resistant (XDR) S. Typhi (resistant to fluoroquinolones, third-gen cephalosporins, and azithromycin) | Diagnosis: Blood cultures required before antibiotic initiation .Empiric therapy: Azithromycin for uncomplicated cases; carbapenems (e.g., meropenem) for XDR/complicated cases Tailored therapy: Adjust based on susceptibility testing (e.g., carbapenems for XDR strains) | Integrated with antimicrobial stewardship programs, resistance surveillance, and clinician training | Emphasizes hygiene, safe food/water practices, and vaccination for travelers | [56] |
|  |  | PCS COPD Guideline | 2020 | Evidence based | PCS | Local | Not explicitly address local or regional antimicrobial resistance patterns | Not mentioned | mentions antibiotic use in COPD exacerbations but does not explicitly address antimicrobial stewardship or patient compliance. | Patient counseling on antibiotic adherence | [57] |
|  |  | MMIDSP,  IDSP & PARN Guideline | 2019 | Evidence-based recommendations and expert consensus | MMIDSP,  IDSP/ PARN | Local | Addresses resistance in E. coli, Klebsiella, and Staphylococcus aureus (MRSA | Recommends culture-guided therapy and restriction of broad-spectrum antibiotics | Includes stewardship training and audit systems | Encourages adherence to prescribed regimens | [58] |
|  |  | Pakistan Chest Society. Guidelines for CAP  in adults | 2017 | Evidence-based recommendations, expert consensus, and local resistance data | PCS | National | Addresses resistance in Streptococcus pneumoniae (penicillin resistance) and Haemophilus influenzae | Recommends amoxicillin as first-line therapy; macrolides for atypical pathogens or penicillin allergy | Includes severity stratification (CURB-65 score) and microbiological testing protocols | Emphasizes smoking cessation and vaccination | [59] |
|  |  | SGP  Pakistan | 2015 | Evidence-based, locally adapted sepsis management | AKU | Local | Targets resistance in Gram-negative bacteria (e.g., Acinetobacter, Klebsiella) | Recommends empiric piperacillin-tazobactam for sepsis; discourages carbapenem overuse | Training programs and sepsis protocol integration | Education on early recognition of sepsis | [60] |
| Low income | Somalia | Somali STGs | 2015 | Evidence-based recommendations aligned with WHO EML | MOH | National | Addresses resistance in Salmonella typhi and E. coli | Recommends first-line antibiotics and discourages empiric broad-spectrum use | Integrated with stewardship programs and formulary management | Community education on rational antibiotic use | [61] |
| **European Region** | | | | | | | | | | | |
| **High Income** | **Czech Republic** | Czech CDI Guidelines | 2022 | Evidence Based Guideline updated version | DID | Local | Addresses resistance in C. difficile (e.g., metronidazole resistance) and risks of recurrence | Recommends fidaxomicin or vancomycin as first-line therapy; limits metronidazole to mild cases in resource-limited settings | Includes diagnostic algorithms, stewardship programs, and monitoring protocols | Emphasizes patient education on adherence and infection prevention | [62] |
|  | **Denmark** | Danish Antibiotic Guidelines | 2013 | evidence-based practices, Labl testing, and rational use of antibiotics | Danish Health & Medicines Authority | National | Addresses antibiotic resistance trends (e.g., resistance in E. coli, Staphylococcus aureus) | Emphasizes rational antibiotic use, first-line and alternative options based on resistance patterns | Physicians must ensure antibiotics are used only, when necessary, based on microbiological testing. Hospitals and departments must have specific instructions for antibiotic use.The goal is to reduce antibiotic resistance by limiting unnecessary use | . Includes patient education materials | [63] |
|  | **Germany** | German LRTI Guidelines | 2024 | Clinical Experts collaborated on recommendations | GSPID /AWMF | National | Addresses resistance risks in Pseudomonas aeruginosa and other multidrug-resistant pathogens common in this population | Recommends tailored antibiotic regimens (e.g., piperacillin-tazobactam for Pseudomonas); emphasizes microbiological testing and de-escalation | Includes multidisciplinary care teams, stewardship programs, and outcome monitoring | Emphasizes caregiver education on infection prevention and adherence | [64] |
|  |  | Pediatric CAP Guidelines | 2020 | Evidence-based review, expert consensus | By PCAP DGPI/GPP | National | Addresses resistance in Streptococcus pneumoniae (e.g., penicillin resistance) and Mycoplasma pneumoniae | Recommends amoxicillin as first-line therapy; limits macrolides to atypical pneumonia or penicillin allergy | Includes severity stratification, microbiological testing protocols, and stewardship alignment with national ABS programs | Emphasizes vaccination (pneumococcal, influenza) and caregiver education | [65] |
|  |  | German ABS Guideline | 2016 | Evidence-based grading according to the AWMF Guidance Manual | GSID | International | Targets resistance in pathogens like MRSA, VRE, multidrug-resistant Gram-negative bacteria, and Clostridium difficile | Recommends tailored antibiotic selection, dosing, and duration; emphasizes de-escalation and stewardship to minimize resistance risks | . It includes recommendations for implementing antibiotic stewardship programs and highlights the importance of evidence-based practices. | Limited explicit guidance; focuses on clinician training and adherence | [66] |
|  |  | German Tonsillitis Guideline | 2015 | Evidence-based review, expert consensus | GSO, HNS | National | Addresses resistance in Streptococcus pyogenes (e.g., penicillin resistance) and other pathogens | Recommends penicillin V as first-line therapy; limits macrolides to penicillin allergy or resistance | Includes diagnostic algorithms (e.g., Centor score), microbiological testing, and stewardship alignment with national ABS programs | Emphasizes patient education on adherence and symptom management | [67] |
|  |  | German CAPNETZ Guidelines | 2009 | Key points based on Oxford Center for Evidence-Based structure. | Paul-Ehrlich-SC, GRS, GSI, CAPNETZ | National | Addresses resistance in Streptococcus pneumoniae (e.g., penicillin resistance) and atypical pathogens (e.g., Legionella) | Recommends amoxicillin as first-line therapy; macrolides for atypical pneumonia or penicillin allergy | Includes severity stratification (CRB-65 score), microbiological testing protocols, and stewardship alignment with national ABS programs | Emphasizes vaccination (pneumococcal, influenza) and smoking cessation | [68] |
|  | **Ireland** | CHI Antimicrobial Guidelines | 2020 | Evidence-based recommendations, local resistance data, expert consensus | CHI | National | Addresses local antibiotic resistance patterns (e.g., resistance in Streptococcus pneumoniae, E. coli | Recommends antibiotics based on local resistance patterns; promotes antimicrobial stewardship | Provides clear dosing, duration, and monitoring guidelines for compliance | Includes guidance for caregivers | [69] |
|  |  | CHI Antimicrobial Guideline | 2016 | Two-round modified Delphi consensus method. | DGP at the RCS Surgeons – Clinical-CPRG | Local | Addresses local antibiotic resistance patterns (e.g., resistance in Streptococcus pneumoniae, E. coli | Recommends antibiotics based on local resistance patterns; promotes antimicrobial stewardship | Provides clear dosing, duration, and monitoring guidelines for compliance | includes guidance for caregivers | [70] |
|  | **Italy** | UTI-Ped-ER Guidelines | 2023 | GRADE | UTI-Ped-ER | Local | Addresses resistance in E. coli (e.g., trimethoprim-sulfamethoxazole resistance) | Recommends antibiotic prophylaxis (e.g., nitrofurantoin, cefixime) only for high-risk cases; discourages routine prophylaxis to reduce resistance | Includes risk stratification, microbiological testing, and stewardship alignment | Emphasizes caregiver education on hygiene and adherence | [71] |
|  |  | SITA-SIP COVID-19 Guidelines | 2021 | GRADE | SITA/SIP | National | Addresses antibiotic resistance risks from unnecessary use in viral infections | Recommends antibiotics only for confirmed bacterial co-infections (e.g., azithromycin for atypical pneumonia); discourages empiric broad-spectrum use | Integrated with national COVID-19 protocols, stewardship programs, and outcome audits | Includes public awareness campaigns on antibiotic misuse | [72] |
|  | **Netherland** | SWAB CAP Guideline | 2024 | GRADE | SWAB/NVALT | National | Addresses antibiotic resistance in CAP pathogens (e.g., macrolide-resistant Streptococcus pneumoniae) | Recommends empiric antibiotics tailored to local resistance; emphasizes stewardship and de-escalation | Includes diagnostic algorithms, monitoring protocols, and stewardship tools | Provides guidance on adherence and symptom monitoring | [73] |
|  |  | CDSS-Antibiotics-2022 | 2022 | developed (CDSS for empirical antibiotic therapy, involving stakeholders | Erasmus MC,UMC | Local | Antimicrobial resistance in common pathogens (e.g., E. coli, Klebsiella spp., S. aureus, P. aeruginosa) | Use guideline-based CDSS to guide empiric antibiotic selection. Tailor therapy to local resistance patterns (e.g., ESBL prevalence). Avoid unnecessary antibiotics for non-bacterial infections De-escalate therapy based on culture results. | Implementation of CDSS in electronic health records (EHRs)Antibiotic stewardship programs to monitor compliance. Track resistance rates via hospital surveillance systems. Provider training on CDSS use. | Provides guidance on adherence and symptom monitoring | [74] |
|  | **Russia** | COPD Guidelines | 2018 | Evidence-based review, expert consensus | RRS | National | Addresses antibiotic resistance in COPD exacerbations (e.g., resistance in Haemophilus influenzae) | Recommends antibiotics for exacerbations based on resistance patterns and clinical severity | Provides treatment algorithms, monitoring, and follow-up strategies | Includes patient education on COPD management | [75] |
|  | **Scotland** | CDI Guidelines | 2014 | Evidence-based recommendations and expert consensus | NHS, HPN ,HPS | National | Addresses resistance in Clostridium difficile | Recommends antibiotic stewardship, infection control measures, and targeted therapy for CDI | Includes surveillance, audits, and feedback mechanisms | Patient education on hygiene and antibiotic use | [76] |
|  | **Slovenia** | Slovenia Neurosurgical Guidelines | 2014 | Evidence-based recommendations for neurosurgical prophylaxis | SMA | National | Targets resistance in Staphylococcus aureus and Gram-negative bacteria | Recommends single-dose prophylaxis and avoidance of prolonged antibiotic use | Includes compliance audits and feedback mechanisms | Patient education on surgical site infection prevention | [77] |
|  | **Spain** | MSF Clinical Guidelines | 2024 | Evidence-based, context-adapted protocols for resource-limited settings | MSF | International | Addresses resistance in common infections (e.g., antibiotic-resistant Salmonella, TB, malaria) | Recommends context-specific antibiotic use, prioritizes first-line agents, and discourages empiric broad-spectrum antibiotics | Integrated with MSF field training, adherence monitoring, and outcome audits | Advises on routine preventive measures and regular follow-up to avoid complications. | [78] |
|  |  | V Spanish Consensus Guidelines | 2022 | systematic review of scientific evidence,Delphi process & GRADE | VSCC | National | Targets resistance in H. pylori to clarithromycin (15–20%) and metronidazole (30–40%) | First-line therapy: 14-day non-bismuth quadruple (PPI + clarithromycin + amoxicillin + metronidazole) or bismuth quadruple (PPI + bismuth + tetracycline + metronidazole) Rescue therapy: Levofloxacin-based regimens for clarithromycin-resistant strains.Avoid triple therapies (PPI + two antibiotics) due to suboptimal efficacy (<80%) | Stewardship programs, compliance audits (98% adherence in Spanish studies), and resistance surveillance | Emphasizes adherence to complex regimens and side-effect management (e.g., stool discoloration with bismuth) | [79] |
|  | **Sweden** | Swedish CAP Guidelines | 2012 | Evidence-based review, expert consensus | SSID | National | Addresses Streptococcus pneumoniae (low resistance to penicillin) and avoids broad-spectrum use | Recommends narrow-spectrum antibiotics (e.g., penicillin G) for mild-moderate CAP; combination therapy (cefotaxime/macrolide) for severe cases | Includes CRB-65 scoring, microbiological testing protocols, and stewardship alignment with Strama programs | Emphasizes smoking cessation and vaccination (influenza/pneumococcal) | [80] |
|  | **Switzerland** | Swiss PAP Guidelines | 2022 | Evidence-based, database-informed surgical prophylaxis | PIGS | National | Addresses resistance risks from overuse of broad-spectrum antibiotics (e.g., amoxicillin/clavulanic acid | Recommends narrow-spectrum agents (e.g., cefazolin) for most procedures; limits duration to ≤24 hours to reduce resistance risks | Includes compliance audits and stewardship programs; highlights gaps in adherence requiring educational interventions | Limited explicit guidance; focuses on clinician adherence | [81] |
|  | **Trinidad and Tobago** | T&T ARI Guidelines | 2020 | GRADE | MOH | National | Targets resistance due to overuse of amoxicillin, co-amoxiclav, and macrolides | Not mentioned | Training programs for GPs, public health campaigns, and resistance surveillance | Educates caregivers on avoiding unnecessary antibiotic use | [82] |
|  | **UK** | GMMMG Guideline | 2024 | NICE evidence based | by Greater Manchester HCCM team | Local | Local resistance patterns for a wide range of pathogens (e.g., Escherichia coli, Staphylococcus aureus, Pseudomonas aeruginosa | Tailor antibiotic therapy to local resistance patterns. Promote antimicrobial stewardship. Use narrow-spectrum antibiotics where appropriate | Regular audits of antibiotic prescribing. Monitor resistance patterns and adherence. Outcome measures: Reduced antimicrobial resistance and improved patient outcomes | Educate patients on appropriate antibiotic use. Provide info on completing courses and recognizing side effects. Advise on preventive measures (e.g., vaccination) | [83] |
|  |  | BSW ICB Guideline | 2024 | evidence based | BSW-ICB | Local | Local resistance patterns for common pathogens (e.g., Escherichia coli, Staphylococcus aureus, Streptococcus spp.). | Tailor antibiotic therapy to local resistance patterns. Promote antimicrobial stewardship. Use narrow-spectrum antibiotics where appropriate | Regular audits of antibiotic prescribing. Monitor resistance patterns and adherence. Outcome measures: Reduced antimicrobial resistance and improved patient outcomes. | Educate patients on appropriate antibiotic use. Provide info on completing courses and recognizing side effects. Advise on preventive measures | [84] |
|  |  | Penumonia NICE Guideline | 2023 | GRADE | NICE | International | Targets antibiotic misuse through stewardship; addresses resistance linked to broad-spectrum use | CRB65/CURB65 scoring for severity assessment; Avoid routine glucocorticoids; 3. Timely antibiotic therapy within 4 hours of hospital admission | Integrated with NHS audits, stewardship programs, and compliance monitoring | Explains symptom timelines (e.g., fever resolution in 1 week) | [85] |
|  |  | BTS Guideline | 2020 | GRADE | BTS | National | Recommendations consider the risk of macrolide resistance and advocate for judicious use | Long-term macrolides should be considered for reducing exacerbations in COPD and bronchiectasis. Monitor for macrolide resistance and side effects. Use should be individualized and reviewed regularly. | Regular monitoring of patient response and side effects. Audits of macrolide prescribing practices. Reduction in exacerbation frequency, improved quality of life, and minimized resistance | Educate patients on the benefits and risks of long-term macrolide therapy. Discuss the importance of adherence and monitoring for side effects. Provide information on managing chronic respiratory conditions | [86] |
|  |  | UK BA SH HIV national guideline | 2020 | Evidence-based review, expert consensus | BASHH | National | Recommendations consider local resistance patterns for sexually transmitted infections (STIs) and urinary pathogens (e.g., Chlamydia trachomatis, Neisseria gonorrhoeae, Escherichia coli). | - Empirical antibiotic therapy based on likely pathogens (e.g., ceftriaxone + doxycycline for STIs, ofloxacin for urinary pathogens). Adjust therapy based on culture results and local resistance patterns. Partner notification and treatment for STIs | . Regular audits of antibiotic prescribing practices. Monitoring of resistance patterns and adherence to guidelines. Outcome measures: Reduction in complications, recurrence rates, and STI transmission. | Educate patients on the importance of completing antibiotic courses. Provide information on safe sexual practices and partner notification. Advise on recognizing complications and seeking timely medical care. | [87] |
|  |  | AMP in Dentistry-GPG | 2020 | GRADE | FDS-RCS | National | Local resistance patterns for oral pathogens (e.g., Streptococcus spp., anaerobes). | Antibiotics only for spreading infection/systemic involvement. First-line: Amoxicillin or phenoxymethylpenicillin. Avoid overuse of broad-spectrum antibiotics | Regular audits of antibiotic prescribing. Monitor resistance patterns and adherence Outcome measures: Reduced inappropriate antibiotic use | Educate on appropriate antibiotic use for dental conditions. Advise on oral hygiene and preventive measures. Provide info on recognizing signs of infection | [88] |
|  |  | HAP  NICE Guideline | 2019 | Evidence-based review, expert consensus | NICE | International | Recommendations consider local antibiotic resistance patterns, particularly for Streptococcus pneumoniae and other common pathogens | Use CURB-65 or CRB-65 to guide antibiotic choice and hospitalization.  Empirical antibiotic therapy tailored to and local resistance patterns and severity  Avoid overuse of broad-spectrum antibiotics. | Regular audits of antibiotic prescribing practices. Monitoring of resistance patterns and adherence to guidelines.  Outcome measures: Reduction in mortality, hospitalization rates, and antibiotic resistance | Educate patients on completing antibiotic courses as prescribed.  Provide information on vaccination (pneumococcal and influenza).Advise on recognizing worsening symptoms and seeking timely medical care. | [89] |
|  |  | CAP  APG | 2019 | GRADE | NICE | International | Antimicrobial resistance in S. pneumoniae, H. influenzae, M. pneumoniae | First-line therapy: Amoxicillin for low-severity CAP; amoxicillin + macrolide for moderate-severity CAP. Avoid broad-spectrum antibiotics (e.g.,fluoroquinolones) unless resistance or allergy concerns. Limit duration (5 days for low/moderate severity; 7–10 days for severe CAP). Tailor therapy based on local resistance data | Antibiotic stewardship programs in primary and secondary care.  Diagnostic tools (e.g., CURB-65, CRB-65 scores) to guide therapy.  Track resistance rates via UK surveillance systems.  4.Audit-and-feedback for prescribers. | Emphasizes adherence to therapy and recognition of complications | [90] |
|  |  | BSAC Guideline ED | 2012 | Evidence-based review, expert consensus | By BSAC | National | Local resistance patterns for common pathogens (e.g., Staphylococcus aureus, Streptococcus spp., Enterococcus spp.) | Empirical antibiotic therapy based on likely pathogens. Tailor therapy based on culture results and local resistance patterns. Prolonged antibiotic courses for effective treatment | Regular audits of antibiotic prescribing. Monitor resistance patterns and adherence. Outcome measures: Reduced mortality and complications. | Educate patients on the importance of completing antibiotic courses. Provide info on recognizing symptoms of complications. Advise on preventive measures (e.g., dental hygiene) | [91] |
| **Upper Middle income** | **Georgia** | GPAS CAP Guidelines | 2023 | GRADE | Children health care | National | Evidence based | Briefly mentioned | Integrated with NHS audits, stewardship programs, and compliance monitoring | Explains symptom timelines | [92] |
| **Regions of the Americas** | | | | | | | | | | | |
| **High Income** | **Canada** | CCPG-Rhinosinusitis | 2011 | Evidence-based review, expert consensus | CSOHNS, CRWG | National | this guideline does not reflect current resistance trends | Avoid antibiotics for viral rhinosinusitis (e.g., symptoms <10 days without severity). First-line therapy: Amoxicillin or amoxicillin-clavulanate for bacterial rhinosinusitis. Limit duration (5–7 days for acute cases). Avoid macrolides due to high pneumococcal resistance | Diagnostic criteria standardization. Antibiotic stewardship programs to reduce overprescribing. Track resistance rates via Canadian surveillance systems. | Educate patients on self-care (e.g., nasal saline, analgesics). Counsel on avoiding antibiotics for viral cases. Communicate risks of antibiotic resistance | [93] |
|  |  | CA-MRSA-2006 | 2006 | Evidence-based review, expert consensus | CMA, CIDS | Local | MRSA resistance to β-lactams, macrolides, fluoroquinolones | Empiric therapy: Trimethoprim-sulfamethoxazole (TMP-SMX) or doxycycline for mild/moderate CA-MRSA infections. Reserve vancomycin for severe or invasive infections. Avoid unnecessary antibiotics to reduce resistance risk. Promote infection control measures (e.g., hand hygiene, wound care). | Surveillance of CA-MRSA prevalence and resistance trends. Antibiotic stewardship programs to optimize therapy. Infection control protocols in healthcare settings. Provider education on CA-MRSA management | Educate patients on wound care and hygiene. Counsel on adherence to prescribed regimens. Communicate risks of antibiotic overuse (e.g., resistance, side effects). | [94] |
|  | **USA** | IDSA Guidelines | 2024 | GRADE | IDSA | International | Targets resistance in CRE, ESBL-producing Enterobacteriaceae, and carbapenem-resistant P. aeruginosa (CRPA) | Recommends newer β-lactam/β-lactamase inhibitors (e.g., ceftazidime-avibactam) and cefiderocol for CRE/CRPA; limits colistin use | Includes stewardship programs, rapid diagnostics (e.g., PCR, MALDI-TOF), and outcome audits | Emphasizes adherence to therapy and infection control | [95] |
|  |  | Sanford-VAP- | 2024 | evidence-based recommendations. | Jay P. Sanford, M.D. in 1969 | International | MDR pathogens: MRSA, Pseudomonas aeruginosa, Acinetobacter baumannii, ESBL-producing Enterobacterales, carbapenem-resistant organisms (CRO) | Empiric broad-spectrum therapy (e.g., anti-MRSA + antipseudomonal β-lactam + aminoglycoside) in high-risk/MDR settings. DE-escalate based on culture/susceptibility results. Limit duration of therapy (7–8 days) to reduce resistance risk. Avoid unnecessary antibiotics in colonization vs. true infection | Antibiotic stewardship programs for ICU-specific protocols. Surveillance of local ICU resistance trends. Routine microbiologic testing (e.g., bronchoalveolar lavage cultures) | Not mentioned | [96] |
|  |  | CDC-Antibiotic- | April 2024 | evidence-based practices & expert consensus | By CDC | National | Antimicrobial resistance in common outpatient infections (e.g., Streptococcus pneumoniae, Escherichia coli, Staphylococcus aureus) | Avoid antibiotics for viral infections (e.g., common cold, viral bronchitis). Use first-line agents (e.g., amoxicillin for sinusitis, nitrofurantoin for uncomplicated UTIs). Limit duration of therapy (e.g., 5–7 days for most infections). Avoid fluoroquinolones for uncomplicated infections due to resistance and side effects. | Antibiotic stewardship programs in outpatient settings. Diagnostic tools (e.g., rapid strep tests, urine cultures) to guide therapy. Track prescribing patterns via EHR audits and feedback | Educate patients on when antibiotics are unnecessary (e.g., viral infections). | [97] |
|  |  | SCCM-Corticosteroids | May 2024 | GRADE Approach used. | SCCM | international | his guideline does not directly address antimicrobial resistance but emphasizes monitoring for secondary infections due to corticosteroid-induced immunosuppression. | Corticosteroids recommended for severe CAP, ARDS, and septic shock with high inflammatory response. Avoid routine use in mild CAP or sepsis without shock to prevent adverse effects. Monitor for secondary infections (e.g., fungal, bacterial) due to immunosuppression | Protocols for corticosteroid use in ICUs and general wards. Monitoring for complications (e.g., hyperglycemia, infections). Stewardship programs to guide appropriate use and avoid overuse | Educate patients/families on risks/benefits of corticosteroids. Counsel on monitoring for side effects (e.g., infections, hyperglycemia). Provide post-discharge follow-up for long-term complications. | [98] |
|  |  | IWGDF/IDSA-DFI | 2023 | GRADE | IWGDF, IDSA | International | MRSA, Pseudomonas aeruginosa, ESBL-producing Enterobacterales, carbapenem-resistant organisms (CRO | Empiric broad-spectrum therapy (e.g., vancomycin + piperacillin-tazobactam) for severe infections in high-resistance regions. De-escalate to narrow-spectrum agents once culture/susceptibility results are available. Avoid prolonged antibiotic courses. Tailor therapy to local antibiograms (e.g., MRSA prevalence). | Multidisciplinary care teams (e.g., podiatrists, infectious disease specialists). Antibiotic stewardship programs in diabetic foot clinics. Surveillance of local resistance trends. Standardized wound cultures to guide therapy | Educate on foot care to prevent infections. Counsel on adherence to antibiotic regimens. Teach recognition of infection signs (e.g., redness, swelling). Promote glycemic control for healing | [99] |
|  |  | Diabetic Foot IG | 2023 | Grade | (IWGDF/IDSA | International | Targets resistance in Staphylococcus aureus (MRSA), Pseudomonas aeruginosa, and ESBL-producing Gram-negative bacteria | Use clinical signs and severity classification for infection Antibiotics: Avoid empiric P. aeruginosa coverage in temperate climates; reserve for confirmed cases in Asia/North Africa Surgery: hyperbaric oxygen | - Stewardship programs  - Compliance audits  - Multidisciplinary teams (surgeons, infectious disease specialists) | Emphasize adherence to treatment, wound care, and prevention of recurrence | [99] |
|  |  | Michigan UTI Guidelines | 2021 | Evidence-based review, expert consensus | MHHA | International | Targets resistance in E. coli (ESBL-producing), Klebsiella pneumoniae, and Enterococcus | First-line therapy: Nitrofurantoin or trimethoprim-sulfamethoxazole for uncomplicated UTIs. Avoid fluoroquinolones unless resistance confirmed .Limit treatment to 3–7 days for uncomplicated cases | Stewardship programs, automated alerts for inappropriate prescriptions, and urine culture audits | Emphasizes adherence to short-course therapy and for recurrence prevention | [100] |
|  |  | AAP-RedBook- | 2021 | GRADE | AAP | International | MRSA, penicillin-resistant S. pneumoniae, ESBL-producing Enterobacter ales, multidrug-resistant TB, antifungal resistance | Tailor empiric antibiotics to local resistance patterns (e.g., MRSA coverage for severe infections). Avoid unnecessary antibiotics for viral infections. Use narrow-spectrum agents when possible. Promote stewardship in pediatric settings | Stewardship programs in pediatric hospitals/clinics. Surveillance of resistance trends via CDC networks. Vaccination advocacy to reduce antibiotic use (e.g., pneumococcal, influenza vaccines). Guideline dissemination to pediatric providers | Counsel on vaccine adherence to prevent resistant infections. Educate caregivers. Communicate risks of antibiotic misuse | [101] |
|  |  | IWGDF/IDSA-DFI | 2020 | GRADE | (IWGDF/IDSA | International | Targets resistance in Staphylococcus aureus (MRSA), Pseudomonas aeruginosa, and ESBL-producing Gram-negative bacteria | Use clinical signs and severity classification for infection Antibiotics:: hyperbaric oxygen | - Stewardship programs  - Compliance audits | Emphasize adherence to treatment, wound care, and prevention of recurrence | [102] |
|  |  | ATS CAP Guideline | 2019 | GRADE | ATS &IDSA | International | Streptococcus pneumoniae, atypical pathogens, and antibiotic-resistant organisms | Recommendations for empiric and targeted antibiotic therapy based on resistance patterns and patient risk factors | discussed. methods to implement guideline recommendations effectively., | Education on medication adherence, signs of worsening illness, and prevention | [103] |
|  |  | IDSA OPAT Guidelines | 2018 | GRADE | IDSA | International | Antimicrobial resistance patterns for specific pathogens (e.g., MRSA, resistant Gram-negative bacteria) | Recommendations for antimicrobial selection, monitoring, and duration based on resistance profile. | Strategies for adherence, monitoring treatment outcomes, and follow-up | Education on medication administration and side effects. | [104] |
|  |  | IDSADiarrhea | 2018 | GRADE | IDSA | International | Antimicrobial resistance in enteric pathogens (e.g., Campylobacter, Shigella, C. difficile | 1. Avoid antibiotics for most cases of acute diarrhea. Reserve antibiotics for severe cases or specific pathogens (e.g., Shigella, C. difficile). Use targeted therapy based on culture/susceptibility results. Avoid fluoroquinolones for Campylobacter due to rising resistance | Diagnostic testing (e.g., stool cultures, PCR) for high-risk cases. Antibiotic stewardship programs to reduce inappropriate prescribing. Track resistance trends via public health surveillance. | Educate patients on oral rehydration and when to seek care. Counsel on hygiene to prevent transmission. Communicate risks of unnecessary antibiotics) | [105] |
|  |  | IDSA/SHEA CDI Guidelines | 2017 | GRADE | IDSA/SHEA | International | Addresses hypervirulent strains (e.g., ribotype 027) and recurrence risks due to antibiotic misuse | Recommends fidaxomicin or vancomycin for initial episodes; limits metronidazole to mild cases in resource-limited settings | Includes stewardship programs, infection control protocols, and compliance audits | Emphasizes hand hygiene and avoidance of unnecessary antibiotics | [106] |
|  |  | Adult & Pediatric APG | 2017 | Evidence-based, systematic review consensus | Department of Health | National | Macrolides like azithromycin are not recommended due to high levels of resistance in S. pneumoniae (~40%).In Acute Pharyngitis: There is increasing resistance to clindamycin and azithromycin among Group A Streptococcus (GAS) | Acute Rhinosinusitis Macrolides like azithromycin are not recommended due to high resistance in S. pneumoniae (~40%). Amoxicillin/clavulanate is recommended if mild/moderate with no risk factors for resistance. In Acute Pharyngitis: Increasing resistance to clindamycin and azithromycin among Group A Streptococcus (GAS | No information provided | Not mentioned | [107] |
|  |  | AAO-HNS-OME | 2016 | Evidence-based review, expert consensus | AAO-HNS | International | Antimicrobial resistance in S. pneumoniae, H. influenzae, M. catarrhalis | Avoid antibiotics for OME unless acute infection is present. Use first-line agents (e.g., amoxicillin) for acute otitis media with effusion. Limit duration of therapy. Avoid unnecessary antibiotics. | Diagnostic criteria standardization (e.g., tympanometry, otoscopy). Antibiotic stewardship programs in pediatric clinics. Track prescribing patterns via EHR audits. | . Educate caregivers on the self-limiting nature of OME. Counsel on avoiding antibiotics for asymptomatic cases. Provide written materials on OME management | [108] |
|  |  | ACG-Diarrhea- | 2016 | Evidence-based review, expert consensus | ACG | International | Antimicrobial resistance in enteric pathogens (e.g., Campylobacter, Shigella, E. coli, Salmonella) | Avoid antibiotics for mild/self-limiting diarrhea (viral or non-invasive bacterial). Reserve antibiotics for severe cases, immunocompromised patients, or traveler’s diarrhea (e.g., azithromycin, rifaximin). Avoid fluoroquinolones in regions with high Campylobacter resistance. Tailor therapy to local resistance and travel history. | Diagnostic testing (stool cultures, PCR) for high-risk cases. Antibiotic stewardship programs to reduce inappropriate use. Track resistance trends via public health surveillance. | Educate on oral rehydration and dietary management. Counsel on hand hygiene and food safety to prevent transmission. Advise against self-treatment with antibiotics for mild cases | [109] |
|  |  | ACP/CDC-ARTI | 2016 | evidence-based practices. | ACP/CDC | National | Outdated Guideline: Published in 2016, this guideline does not reflect current resistance patterns (e.g., rising macrolide resistance in S. pneumoniae) | Avoid antibiotics for uncomplicated bronchitis (viral etiology in >90% of cases). Use first-line agents (e.g., amoxicillin for bacterial sinusitis, penicillin for strep throat).Limit duration of therapy (e.g., 5–7 days for sinusitis). | Antibiotic stewardship programs in outpatient clinics. Diagnostic tools (e.g., rapid strep tests, clinical scoring systems).Audit-and-feedback for prescribers. Track prescribing patterns via EHR audits. | Provided counseling on duration of symptoms and the potential harms of inappropriate antibiotic use. | [110] |
|  |  | AAO-HNS Sinusitis Guideline | 2015 | GRADE | AAO-HNS | International | Indirectly addresses resistance via antibiotic stewardship | Avoid antibiotics for viral sinusitis .Limit antibiotics to bacterial cases meeting criteria (e.g., symptoms >10 days or severe onset) | Diagnostic tools (e.g., symptom criteria) Antibiotic stewardship programs.Adherence to guideline criteria | Educate on appropriate antibiotic use, symptom relief and prevention | [111] |
|  |  | IDSA-SSTI- | 2014 | Evidence-based review, expert consensus | IDSA | International | MRSA, β-lactam resistance in streptococci, gram-negative resistance in diabetic foot infections | Empiric MRSA coverage (e.g., doxycycline, clindamycin, TMP-SMX) for purulent infections in high-prevalence areas. De-escalate antibiotics once cultures confirm susceptibility. Avoid antibiotics for uncomplicated abscesses after drainage. Use narrow-spectrum agents (e.g., cephalexin) for non-purulent cellulitis | Antibiotic stewardship programs to guide empiric therapy. Culture-guided therapy for severe/complicated infections. Monitor local MRSA prevalence and resistance trends | Educate on wound care and signs of worsening infection. Counsel on completing prescribed antibiotics. Avoid sharing personal items to prevent MRSA spread | [112] |
|  |  | ASHP-Surgical- | 2013 | GRADE | ASHP, IDSA, SIS, and SHEA | International | Resistance in surgical pathogens (e.g., MRSA, ESBL-producing Enterobacterales, C. difficile) | Use narrow-spectrum agents (e.g., cefazolin) for most procedures. Limit duration (single dose pre-incision; discontinue within 24 hours post-op). Avoid vancomycin/fluoroquinolones unless high MRSA risk or β-lactam allergy. Tailor therapy to local resistance patterns (e.g., MRSA prevalence | Pre-operative checklists for antibiotic timing/dosing. Stewardship programs to audit compliance. Surveillance of surgical site infections (SSIs) and resistance trends. Provider education on guideline adherence. | Counsel on wound care to prevent infection. Educate on risks of unnecessary antibiotics (e.g., C. difficile). Post-discharge instructions for adherence to prophylaxis (if extended). | [113] |
|  |  | IDSA ABRS Guideline | 2012 | GRADE | IDSA | International | Streptococcus pneumoniae, Haemophilus influenzae, Moraxella catarrhalis (focus on penicillin resistance in S. pneumoniae and beta-lactamase production in H. influenzae) | Amoxicillin-clavulanate as first-line therapy in regions with high penicillin-resistant S. pneumoniae.Avoid macrolides due to resistance. | Antibiotic stewardship, follow-up for treatment failure, and culture-guided therapy for refractory cases | Education on avoiding unnecessary antibiotics, completing prescribed courses, and symptom relief . | [114] |
|  |  | AAP-UTI | 2011 | GRADE | AAP | International | this guideline does not reflect current resistance trends (e.g., rising ESBL-producing E. coli). Refer to updated AAP/IDSA guidelines or local antibiograms for modern recommendations | First-line therapy: Oral amoxicillin/clavulanate or cephalosporins (e.g., cefixime). Avoid TMP-SMX or ampicillin as empiric therapy in regions with high resistance. Tailor therapy based on local susceptibility patterns. Limit antibiotic use to confirmed UTIs (via urinalysis/culture) | Standardize diagnostic criteria (e.g., urinalysis, urine culture). Antibiotic stewardship programs to optimize empiric therapy. Track resistance rates via pediatric antibiograms. Educate providers on guideline adherence. | 1. Counsel caregivers on completing antibiotic courses. Educate on signs of UTI recurrence. Communicate risks of antibiotic overuse (e.g., resistance, side effects). | [115] |
|  |  | CAP Guideline by | 2011 | GRADE | PIDS & IDSA | International | Streptococcus pneumoniae, Mycoplasma pneumoniae, and other pathogens with emerging resistance patterns | Recommendations for antibiotic selection, duration, and escalation based on resistance patterns and severity. | Mentioned Outcome Measures only. | Education on medication Adherence | [116] |
|  |  | ACC/AHA-IE- | 2008 | Evidence-based ,expert consensus | By ACC/AHA | International | Outdated Guideline: Published in 2008, this guideline does not reflect current resistance patterns (e.g., rising vancomycin resistance in Enterococcus or MRSA trends). Refer to updated ACC/AHA guidelines (e.g., 2020) for modern recommendations | Empiric therapy: Vancomycin + ceftriaxone or gentamicin for suspected IE, adjusted based on culture results. Targeted therapy: Use narrow-spectrum agents (e.g., penicillin for streptococci) when susceptibility is confirmed.Avoid prolonged prophylaxis to reduce resistance risk | 1. Antibiotic stewardship programs to optimize IE treatment and prophylaxis. Surveillance of local resistance patterns (e.g., MRSA prevalence). Monitoring adherence to IE prophylaxis guidelines. | Highlights the importance of counseling patients on IE prevention and antibiotic adherence. | [117] |
|  |  | AAO-HNS-Sinusitis- | 2007 | evidence-based recommendations | AAO-HNS | International | General antimicrobial resistance (not explicitly detailed | Reserve antibiotics for confirmed or suspected bacterial sinusitis (e.g., symptoms >10 days or worsening after initial improvement). First-line therapy: Amoxicillin or amoxicillin-clavulanate. Avoid antibiotics for viral sinusitis to reduce resistance risk. Limit duration to 10–14 days for uncomplicated cases | Diagnostic criteria (e.g., symptom duration/severity) to reduce overprescribing. Clinician education on differentiating viral vs. bacterial cases. Track antibiotic prescribing rates in primary care | advises clinicians to educate and counsel patients with chronic rhinosinusitis (CRS) or recurrent acute rhinosinusitis regarding control measures. | [118] |
|  |  | ACCPCough | 2006 | GRADE. | ACCP | International | Outdated.  this guideline does not reflect current resistance patterns | Avoid antibiotics for uncomplicated acute bronchitis (viral etiology in >90% of cases).Reserve antibiotics for suspected bacterial cases (e.g., pertussis, Mycoplasma).Use narrow-spectrum agents (e.g., amoxicillin) if antibiotics are indicated | Diagnostic criteria standardization to differentiate viral vs. bacterial causes. Antibiotic stewardship programs to reduce inappropriate prescribing. Track antibiotic use and resistance trends via EHR audits | . Educate patients on the self-limiting nature of viral bronchitis. Counsel on symptomatic management: Hydration, cough suppressants, rest. Communicate risks of unnecessary antibiotics (e.g., resistance, side effects) | [119] |
|  |  | IDSA GAS-Pharyngitis | 2002 | Evidence-based, systematic review consensus | IDSA | International | Antimicrobial resistance (e.g., macrolide resistance in S. pyogenes | Confirm GAS pharyngitis with rapid antigen detection test (RADT) or throat culture before antibiotic use. First-line antibiotic: Penicillin or amoxicillin (10-day course). Alternative agents: For penicillin-allergic patients, use first-generation cephalosporins, clindamycin, or macrolides (if low resistance). Avoid antibiotics for viral pharyngitis to reduce resistance risk. | 1. Diagnostic standardization: Use RADT or throat culture to confirm GAS. Antibiotic stewardship programs to monitor prescribing practices. Track compliance via EHR audits and feedback to clinicians. | Educate patients/caregivers on the importance of completing the full antibiotic course. Counsel on symptom management: Analgesics, hydration, rest. Communicate risks of unnecessary antibiotics (e.g., resistance, side effects) | [120] |
|  |  | IDSA-ID- | 2001 | Evidence-based systematic review consensus | IDSA | International | Antimicrobial resistance in pathogens (e.g., Campylobacter, Shigella, Salmonella spp. resistance to fluoroquinolones, TMP-SMX) | Limit antibiotic use to severe cases or specific pathogens. First-line agents: Fluoroquinolones for adults, azithromycin for children (if indicated). Avoid empiric antibiotics for mild/self-limiting diarrhea. Adjust therapy based on local resistance patterns. | Diagnostic testing (stool cultures, PCR) for high-risk cases. Antibiotic stewardship programs to reduce inappropriate prescribing. Monitor resistance trends via public health surveillance | Educate on oral rehydration and when to seek care. Hygiene counseling to prevent transmission. Avoid antimotility agents in inflammatory diarrhea. | [121] |
|  |  | AAP-CPG-Sinusitis | 2001 | Evidence-based, systematic review consensus | AAP | National | Antimicrobial resistance (general) | Differentiate viral vs. bacterial sinusitis. Reserve antibiotics for severe or prolonged symptoms. Use amoxicillin as first-line therapy. | Clinician education on diagnostic criteria. Monitoring adherence to antibiotic stewardship principles. Tracking antibiotic prescribing rates | Educate caregivers on avoiding antibiotic overuse. Counsel on symptom management (e.g., nasal saline, analgesics). | [122] |
|  |  | IDSACAP Guideline | 1998 | Expert consensus, literature review | CID & IDSA | International | Streptococcus pneumoniae, Haemophilus influenzae, Mycoplasma pneumoniae (focus on penicillin resistance in S. pneumoniae) | Beta-lactams (e.g., ceftriaxone) + macrolides for empiric therapy. Fluoroquinolones as alternatives in penicillin-allergic patients | Adherence to empiric therapy guidelines, monitoring clinical response, and avoiding overuse of broad-spectrum antibiotics | Education on completing antibiotic courses, recognizing complications (e.g., pleural effusion) | [123] |
| **Upper Middle Income** | **Brazil** | Brazilian CAP Guidelines | 2009 | Evidence-based recommendations, expert consensus, literature review | BTS | National | Addresses resistance in Streptococcus pneumoniae (e.g., penicillin resistance) and atypical pathogens (e.g., Mycoplasma pneumoniae) | Recommends amoxicillin or macrolides (e.g., azithromycin) for outpatient care; respiratory fluoroquinolones for high-risk patients | Includes severity stratification (CURB-65 score), microbiological testing protocols, and stewardship alignment | Emphasizes vaccination (pneumococcal, influenza) and smoking cessation | [124] |
|  |  | Brazilian CAP Guidelines | 2004 | Evidence-based recommendations, expert consensus | BSP | National | Addresses resistance risks in Streptococcus pneumoniae and atypical pathogens (e.g., Mycoplasma pneumoniae, Chlamydia trachomatis) | Recommends: Outpatient: Amoxicillin or penicillin G procaine. Inpatient (severe): Crystalline penicillin/ampicillin. - Very severe: Oxacillin + chloramphenicol or ceftriaxone. Atypical pathogens: Macrolides (e.g., erythromycin) | Integrated with compliance audits and stewardship programs; studies highlight suboptimal adherence (26.1% inadequate empiric therapy) | Emphasizes caregiver education on adherence and vaccination | [125] |
|  | **Guyana** | Guyana STG | 2015 | Evidence-based recommendations, expert consensus, local AMR | MOH | National | Addresses resistance in common pathogens (e.g., E. coli, Streptococcus pneumoniae) | Recommends first-line antibiotics (e.g., amoxicillin, cotrimoxazole) for common infections; discourages overuse of broad-spectrum agents | Integrated with national health programs, training for healthcare workers, and community education | Includes patient education on adherence and prevention | [126] |
|  | **Jamaica** | Jamaican UTI Guidelines | 2018 | Evidence-based review, expert consensus | JKKF | National | Addresses resistance in E. coli (e.g., ESBL-producing strains, trimethoprim-sulfamethoxazole resistance) and other uropathogens | Recommends first-line antibiotics (e.g., nitrofurantoin, cefixime) based on local susceptibility; discourages empiric amoxicillin use | Includes diagnostic algorithms, urine culture protocols, and stewardship alignment with national programs | Emphasizes hydration, adherence, and follow-up testing | [127] |
| **South-East Asia Region** | | | | | | | | | | | |
| **Upper-middle income** | **Maldives** | Maldives NAMSP | 2020 | National policy framework for antimicrobial stewardship and resistance control | Ministry of health | National | Targets resistance in common pathogens (e.g., ESBL-producing Enterobacteriaceae, MRSA) | Recommends stewardship activities (e.g., formulary restrictions, audits) and promotes rational antibiotic use | Includes compliance monitoring, healthcare worker training, and public awareness campaigns | Emphasizes community education on antibiotic misuse prevention | [128] |
|  | **Thailand** | Asthma Guideline | 2022 | Evidance based | daily management, recognition of worsening symptoms, and appropriate responses | National | do not specifically address antibiotic resistance patterns | Promotes rational prescribing | Include compliance monitoring | daily management, recognition of worsening symptoms, and appropriate responses | [129] |
| **Lower middle income** | **Bangladesh** | BSMMU Guidelines | 2023 | Evidence-based recommendations aligned with local antibiograms | BSMMU/WHO | National | Targets resistance in Escherichia coli, Staphylococcus aureus, Pseudomonas aeruginosa, and Klebsiella pneumoniae | Recommends culture-guided therapy, narrow-spectrum antibiotics (e.g., ceftriaxone), and avoidance of imipenem in specific cases | Integrated with stewardship audits; compliance studies show 50–70% adherence in hospital settings | Emphasizes hygiene and adherence to prescribed regimen | [130] |
|  |  | Bangladesh STG | 2021 | Evidence-based protocols aligned with WHO standards | Directorate General of Health Services Ministry of Health and Family Welfare | National | Targets resistance in E. coli, Salmonella typhi, and Vibrio choler | Prioritizes narrow-spectrum antibiotics (e.g., co-trimoxazole); restricts carbapenems for severe cases | Stewardship training, formulary management, and resistance surveillance | Community education on rational antibiotic use | [131] |
|  |  | AP for BIRDEM Hospital | Not specified | Hospital-specific recommendations based on local AMR data, expert consensus | BGH | Hospital level Coverage | Addresses resistance in E. coli, Klebsiella pneumoniae, and Pseudomonas aeruginosa | Recommends empiric therapy (e.g., ceftriaxone for sepsis) with de-escalation based on culture results | Includes stewardship protocols and compliance audits | Limited explicit guidance | [132] |
|  | **Bhutan** | Bhutan-ABG | 2018 | Evidence-based review, expert consensus | MOH | National | Antimicrobial resistance in common pathogens (e.g., S. pneumoniae, E. coli, S. aureus, K. pneumoniae) | Empiric therapy: Use local resistance data to guide treatment (e.g., amoxicillin for respiratory infections). Avoid unnecessary antibiotics for viral infections. Limit duration (e.g., 5–7 days for most infections). Tailor therapy based on culture/susceptibility results. | Antibiotic stewardship programs in healthcare facilities. Surveillance of local resistance trends. Provide education on guideline adherence. | Educate patients on antibiotic risks and self-care for viral illnesses. Counsel on adherence to prescribed regimens. Communicate risks of antibiotic overuse (e.g., resistance, side effects). | [133] |
|  | **Timor Leste** | Antibiotic Guidelines,  HNG | 2016 | Evidence based guidelines | Hospital Nacional Guido Valadares | National | Addresses resistance in E. coli, Klebsiella pneumoniae, and Staphylococcus aureus (e.g., MRSA) | Recommends ceftriaxone and ampicillin as first-line for common infections; restricts carbapenems and vancomycin to severe cases | Includes antimicrobial stewardship training, point-prevalence surveys, and improved microbiological testing capacity | Limited explicit guidance; focuses on clinician training | [134] |
|  | **India** | Indian ICU-IC Guideline | 2019 | Evidence-based recommendations, systematic reviews, expert consensus | ICMR /CCS | Local | targets resistance in Carbapenem-resistant Enterobacteriaceae (CRE), ESBL-producing Gram-negatives, and Azole-resistant Candida | Recommends early empiric broad-spectrum therapy (e.g., carbapenems) with de-escalation based on culture/sensitivity; restricts colistin to confirmed CRE | Stewardship programs, biomarker-guided therapy (e.g., procalcitonin), and audit tools | Counseling on infection prevention and strict adherence to regimens | [135] |
|  |  | ICU Antibiotic Guidelines | 2019 | Evidence-based recommendations, expert consensus, alignment with local AMR data. | ISCCM | National | Targets MRSA, ESBL-producing Enterobacteriaceae, and carbapenem-resistant Pseudomonas | Recommends early microbiological diagnosis, de-escalation based on culture results, and stewardship protocols | Integrated with ASPs, audits, and compliance monito[ring | Emphasizes clinician adherence to protocols | [136] |
|  |  | India AMR Guidelines | 2016 | Evidence-based protocols aligned with WHO GAP | NCDC/ MOH | National | Addresses resistance in Klebsiella pneumoniae, Acinetobacter baumannii, and MRSA | Recommends antibiotic restriction policies, culture-guided therapy, and stewardship training | Includes national surveillance, formulary restrictions, and compliance audits | Mention importance of hand hygiene and infection control measures to reduce the need for antibiotics. | [137] |
|  | **Myanmar** | Myanmar NOGTH Guidelines | 2019 | Evidence-based recommendations aligned with WHO standards to combat AMR | GTH& WHO | Local | Addresses resistance in E. coli (ampicillin resistance) and Klebsiella pneumoniae (ceftriaxone resistance) | Recommends empiric antibiotics (e.g., ceftriaxone, ciprofloxacin) tailored to local resistance patterns | Integrated with AMASS (AutoMated tool for Antimicrobial resistance Surveillance System) for data analysis and reporting | Limited explicit guidance; focuses on clinician adherence | [138] |
|  | **Sri Lanka** | Sri Lanka AMR Guidelines | 2024 | Evidence based guideline. | SCM/MOH/NIM | National | Addresses resistance in Staphylococcus aureus (MRSA), E. coli, and Klebsiella pneumoniae | Recommends early microbiological diagnosis, tailored therapy, and avoidance of unnecessary broad-spectrum use | Integrated with stewardship programs and compliance audits | Emphasizes clinician and patient adherence to prescriptions | [139] |
|  |  | SLMA Guidelines | 2014 | Evidence based Guidelines | SLMA | National | Targets resistance due to misuse of antibiotics in outpatient and inpatient settings | Advises strict adherence to prescriptions, discourages over-the-counter antibiotic sales | Includes regulatory oversight by NMRA and public awareness campaigns | Promotes pharmacist responsibility in dispensing antibiotics | [140] |
| **Western Pacific Region** | | | | | | | | | | | |
| **High Income** | **Australia** | TG Antibiotic | 2024 | Evidence based recommendations. | TGL, | National | Addresses antibiotic resistance trends (e.g., resistance in E. coli, Staphylococcus aureus) | Emphasizes judicious antibiotic use, first-line and alternative options based on resistance patterns | Provides prescribing guidelines, monitoring advice, and stewardship strategies | symptomatic treatment is recommended for most children, with advice to return if symptoms don’t improve within 72 hours. | [141] |
|  |  | CHQ-P  Antibiocard  2022 | 2024 | initial treatment recommendations, AS, daily review and TDM. | CHQP | Local | Addresses local antibiotic resistance patterns (e.g., penicillin-resistant Streptococcus pneumoniae) | Recommends specific antibiotics based on resistance patterns; promotes antimicrobial stewardship | Provides clear dosing, duration, and monitoring guidelines for compliance | Includes guidance for caregivers | [142] |
|  |  | Surgical Antimicrobial Prophylaxis Guideline | 2021 | Evidence-based review, expert consensus | Govt. SAAGAR | Local | South Australia-focused; adaptable to local resistance patterns | Use narrow-spectrum agents (e.g., cefazolin) for most procedures. Limit duration (single dose pre-incision; discontinue within 24 hours post-op).Avoid vancomycin/fluoroquinolones unless high MRSA risk or β-lactam allergy. Tailor therapy to local resistance patterns (e.g., MRSA prevalence). | Pre-operative checklists for antibiotic timing/dosing. Antibiotic stewardship programs to audit compliance. Surveillance of surgical site infections (SSIs) and resistance trends. Provide education on guideline adherence | Counsel on wound care to prevent infection. Educate on risks of unnecessary antibiotics (e.g., C. difficile). Post-discharge instructions for adherence to prophylaxis. | [143] |
|  |  | KHA-CARI UTI Guidelines | 2015 | GRADE | KHA-CARI | National | Indirectly addresses resistance by promoting rational antibiotic use and avoiding overprescribing | Urine culture confirmation before treatment Oral/parenteral antibiotics (e.g., amoxicillin-clavulanate) as first-line. Reserve imaging (renal ultrasound) for confirmed UTIs to detect anomalies | Recommends specific diagnostic and treatment protocols for UTIs in children Regular reassessment and follow-up are suggested. | informed about the importance of avoiding constipation, increasing fluid intake, improving cleaning methods after bowel motions as these measures possibly beneficial for preventing UTIs in children | [144] |
|  | **Darussalam** | Brunei Darussalam  GAPP | 2019 | Stewardship-focused recommendations, expert consensus, and alignment with WHO standards | MOH | National | Targets resistance in Staphylococcus aureus (MRSA) and Pseudomonas aeruginosa | Prioritizes narrow-spectrum antibiotics (e.g., penicillin V) and de-escalation based on culture results | Integrated with antimicrobial stewardship programs (ASPs) and compliance audits | Includes public campaigns on antibiotic misuse prevention | [145] |
|  | **Cook Islands** | CookIslands-ABG | 2023 | Evidence based, local AMR data | MOH | National | Antimicrobial resistance in common pathogens (e.g., S. pneumoniae, E. coli, S. aureus, P. aeruginosa) | Empiric therapy: Use local resistance data to guide treatment (e.g., amoxicillin-clavulanate for respiratory infections) Avoid unnecessary antibiotics for viral infections. Limit duration. Tailor therapy based on culture/susceptibility results. | Antibiotic stewardship programs in healthcare facilities. Surveillance of local resistance trends. Provide education on guideline adherence. Diagnostic tools (e.g., urine cultures, rapid strep tests). | Educate patients on antibiotic risks and self-care for viral illnesses. Counsel on adherence to prescribed regimens. Communicate risks of antibiotic overuse (e.g., resistance, side effects). | [146] |
|  | **Japan** | JGA Chronic Diarrhea Guideline | 2023 | Evidence-based recommendations, expert consensus, and local data | JGA | National | Indirectly addresses resistance by discouraging unnecessary antibiotics (limited evidence for their use in functional diarrhea) | Recommends probiotics and anti-diarrheals as first-line therapies; avoids antibiotics unless secondary infections are confirmed | Includes diagnostic flowcharts, stewardship alignment, and monitoring of treatment efficacy | Emphasizes lifestyle/dietary modifications and adherence to therapy | [147] |
|  |  | JSSI Guideline | 2021 | GRADE Delphi method | JSSI | International | Addresses resistance in common pathogens (e.g., Streptococcus pneumoniae, MRSA, ESBL-producing Enterobacteriaceae) | recommends narrow-spectrum antibiotics (e.g., cefazolin), limits prophylactic duration (≤24h), and avoids universal decolonization (due to mupirocin resistance risks) | Includes compliance audits, stewardship programs, and surveillance of SSI rates | Recommendations include addressing malnutrition, smoking cessation, and alcohol abstinence before surgery.  Prophylactic Antibiotics: Guidelines on the timing and duration of antibiotic administration. | [148] |
|  |  | Japan AMS Manual | 2017 | Evidence-based recommendations, expert consensus, and RCTS. | MOH/LWHSBT/IDCD | National | Addresses resistance in common pathogens (e.g., Streptococcus pneumoniae, MRSA, ESBL-producing Enterobacteriaceae) | Recommends evidence-based antibiotic use, de-escalation, and stewardship programs to reduce resistance risks | Includes multidisciplinary AMS teams, compliance audits, and outcome monitoring | Emphasizes clinician training and patient education on adherence | [149] |
|  |  | jRS CAP Guidelines | 2006 | Evidence-based recommendations, expert consensus, and local data | JRS | National | Addresses resistance in common pathogens (e.g., Streptococcus pneumoniae, MRSA, ESBL-producing Enterobacteriaceae) | Recommends evidence-based antibiotic use, de-escalation, and stewardship programs to reduce resistance risks | Integrated with antimicrobial stewardship programs and hospital compliance audits | Emphasizes vaccination (pneumococcal, influenza) and adherence to treatment | [150] |
|  | **Korea** | Korean AGE Guidelines | 2019 | GRADE | KSID/KSAD | National | Addresses resistance in common pathogens (e.g., Salmonella, E. coli, Campylobacter) | Recommends antibiotics only for severe cases (e.g., dysentery, systemic symptoms) or confirmed bacterial infections to reduce misuse and resistance | Provides treatment algorithms, monitoring protocols, and stewardship tools | Advises against unnecessary antibiotics for viral or mild cases | [151] |
|  |  | Korean UTI Guidelines | 2018 | Evidence-based , local resistance, expert consensus | KSID | National | Addresses antibiotic resistance in UTIs (e.g., resistance in E. coli, Klebsiella pneumoniae) | Recommends antibiotics based on local resistance patterns; promotes antimicrobial stewardship | Provides treatment algorithms, monitoring, and follow-up strategies | Includes patient education on UTI management | [152] |
|  |  | KGU-CAP- | 2018 | Evidence-based review, expert consensus | KGU | National | Antimicrobial resistance in S. pneumoniae, H. influenzae, M. pneumoniae | First-line therapy: Amoxicillin or amoxicillin-clavulanate for low-severity CAP. Avoid unnecessary antibiotics for mild or viral cases. Limit duration (5–7 days for low/moderate severity). Tailor therapy based on local resistance data. | Antibiotic stewardship programs in primary care Diagnostic tools (e.g., CURB-65, CRB-65 scores) to guide therapy. Track resistance rates via national surveillance systems. Provider education on guideline adherence. | Educate patients on completing antibiotic courses Counsel on signs of worsening infection. Communicate risks of antibiotic overuse. | [153] |
|  |  | KGU-ARTI | 2017 | Evidence-based review, expert consensus | KGU | National | Antimicrobial resistance in S. pneumoniae, H. influenzae, S. pyogenes | Avoid antibiotics for viral infections (e.g., common cold, viral pharyngitis).Use first-line agents (e.g., amoxicillin for bacterial sinusitis, penicillin for strep throat).Limit duration (5–7 days for sinusitis, 10 days for strep throat).Avoid macrolides for strep throat due to resistance risks | Diagnostic tools (e.g., rapid strep tests, clinical scoring systems). Antibiotic stewardship programs to reduce overprescribing. Track resistance rates via national surveillance systems. | Educate patients on the self-limiting nature of viral ARTIs. Counsel on symptomatic management (e.g., hydration, rest, analgesics). Communicate risks of unnecessary antibiotics . | [154] |
|  |  | Korea BJI Guidelines | 2014 | GRADE | KSC | National | Targets MRSA, Pseudomonas aeruginosa, and ESBL-producing Enterobacteriaceae (e.g., E. coli) | Empiric therapy: Vancomycin + ceftazidime/cefepime for MRSA and Gram-negative coverage . De-escalate to culture-guided narrow-spectrum agents (e.g., clindamycin for susceptible S. aureus) .Prolonged IV-to-oral therapy (4–6 weeks) to prevent relapse | Multidisciplinary teams (surgeons, infectious disease specialists), stewardship audits, and therapeutic drug monitoring | Counseling on adherence to prolonged regimens and post-surgical wound care | [155] |
|  | **Newzealand** | bpacnz Primary Care Antibiotic Guide | 2024 | Evidence-based review, expert consensus and Local AMR | bpacnz | Local | Addresses antibiotic resistance trends (e.g., resistance in E. coli, Staphylococcus aureus) | Emphasizes appropriate antibiotic selection, duration, and resistance-aware prescribing | Provides prescribing algorithms, monitoring tools, and stewardship resources | Includes patient information leaflets | [156] |
|  |  | bpacnz-ABGuide- | 2017 | Evidence-based review, expert consensus | BPAC | National | Antimicrobial resistance in common pathogens (e.g., S. pneumoniae, H. influenzae, E. coli, S. aureus) | 1. Avoid antibiotics for viral infections (e.g., viral URI, acute bronchitis).Use first-line agents (e.g., amoxicillin for sinusitis, nitrofurantoin for uncomplicated UTIs). Limit duration (e.g., 5 days for uncomplicated UTIs) | 1. Diagnostic tools (e.g., rapid strep tests, urine cultures).Antibiotic stewardship programs in primary careTrack resistance rates via national surveillance systems.Provider education on guideline adherence. | 1. Educate patients on antibiotic risks and self-care for viral illnesses. Counsel on adherence to prescribed regimens.Provide written materials on antibiotic resistance. | [157] |
|  |  | ANZPID-ASAP Guideline | 2016 | Evidence-based recommendations, expert consensus, and local AMR | ANZPID-ASAP) | National | Addresses resistance linked to prolonged antibiotic use (e.g., Staphylococcus aureus, Gram-negative bacteria | Recommends shorter antibiotic courses and early IV-to-oral switch to reduce resistance risks | Provides protocols for duration, monitoring, and stewardship in Paediatric care | Includes caregiver guidance on adherence | [158] |
|  | **Singapore** | Singapore SAP Guideline | 2022 | ADAPTE method, evidence-based grading | NCID | National | Addresses resistance in common pathogens (e.g., MRSA, ESBL-producing Enterobacteriaceae) | Recommends narrow-spectrum antibiotics (e.g., cefazolin) for most procedures; limits duration to ≤24 hours to reduce resistance risks | Includes compliance audits, stewardship programs, and hospital protocols for monitoring | . Limited explicit guidance; focuses on clinician adherence | [159] |
|  | **Taiwan** | Taiwan MDRO Guidelines | 2022 | Evidence-based recommendations, expert consensus, local AMRdata | TSM, IDST | National | Addresses resistance in Carbapenem-resistant Enterobacteriaceae (CRE), MRSA, ESBL-producing Gram-negatives, and Acinetobacter baumannii | Recommends combination therapy (e.g., colistin-carbapenem for CRE), de-escalation post-culture, and reserve antibiotics (e.g., tigecycline) | Stewardship programs, resistance surveillance, and hospital-level audits | Counseling on infection control and adherence to treatment | [160] |
|  |  | Taiwan UP Guidelines | 2011 | Evidence-based consensus guidelines aligned with international standards and local AMR data | TUA | National | Targets resistance in E. coli, Enterococcus spp., and Gram-negative bacilli | . Cefazolin as first-line prophylaxis; 2. Aminoglycosides for high-risk cases; 3. Limit duration to ≤24 hours post-op | Integrated with hospital stewardship programs and surgical audits | Clinician education on adherence to protocols | [161] |
|  |  | Taiwan CAP Guidelines | 2008 | based on epidemiologic data, clinical studies, lab investigations, and imaging studies. | TPA | National | Addresses macrolide-resistant Mycoplasma pneumoniae (12–23% resistance) and penicillin-non-susceptible S. pneumoniae | 1. Amoxicillin (90 mg/kg/day) as first-line for bacterial CAP; 2. Macrolides (e.g., azithromycin) for atypical pathogens; 3. Hospitalization for hypoxemia (SpO₂ <90%) or severe respiratory distress | Integrated with vaccination programs (PCV13, HibCV), clinician training, and surveillance for resistance trends | Emphasizes adherence to antibiotic regimens and symptom monitoring | [162] |
|  |  | Taiwan Surgical Prophylaxis Guidelines | 2004 | Evidence-based recommendations, expert consensus, and local resistance data | IDSC/TSA | National | Addresses resistance in Staphylococcus aureus (MRSA), Gram-negative bacilli (e.g., E. coli) | Recommends single-dose cefazolin for most procedures; emphasizes timing (30-60 mins pre-incision) | Compliance audits, stewardship programs, and cost-reduction strategies | N/A (provider-focused) | [163] |
|  |  | Taiwan UTI Guidelines | 2000 | based on expert consensus and review of existing literature | IDSC/TSA | National | Targets resistance in E. coli, Klebsiella pneumoniae, and ESBL-producing bacteria | Recommends nitrofurantoin or fosfomycin for uncomplicated UTIs; reserves fluoroquinolones for severe cases | Stewardship programs, antibiogram monitoring, and clinician training | Emphasizes adherence to prescribed regimens | [164] |
| **Upper Middle Income** | **China** | Chinese HAP/VAP Guidelines | 2018 | GRADE | CTS, CMA | National | Addresses resistance in Acinetobacter baumannii, Pseudomonas aeruginosa, and Klebsiella pneumoniae (e.g., carbapenem resistance) | Recommends empiric therapy with broad-spectrum β-lactams (e.g., piperacillin-tazobactam) + fluoroquinolones/aminoglycosides; de-escalation based on culture results | Includes severity stratification, microbiological testing, and stewardship programs | Limited explicit guidance; focuses on clinician adherence | [165] |
|  |  | CTS CAP Guidelines  2016 | 2016 | Evidence-based recommendations, expert consensus, and local data | CTS, CMA | National | Addresses resistance in Streptococcus pneumoniae (penicillin non-susceptibility), Mycoplasma pneumoniae (macrolide resistance >80%) | Recommends β-lactams (e.g., amoxicillin/clavulanate) + macrolides or respiratory fluoroquinolones for empiric therapy; avoids macrolide monotherapy | Includes severity stratification (CURB-65), microbiological testing, and stewardship programs | Emphasizes vaccination (pneumococcal, influenza) and adherence | [166] |
|  | **Fiji** | Fiji Antibiotic Guidelines | 2019 | Evidence based, expert opinion | MOH & MSGF | National | Addresses resistance in Leptospira spp., Salmonella spp., and Staphylococcus aureus | Recommends doxycycline for leptospirosis; limits broad-spectrum antibiotics to severe cases | Includes stewardship training and compliance audits | Community awareness campaigns | [167] |
|  | **Malaysia** | Malaysia NAG | 2024 | Evidence-based guidelines evolve over time. | MOH | National | Targets resistance in Acinetobacter baumannii, CRE, and ESBL-producing Enterobacteriaceae | Recommends culture-guided therapy; promotes carbapenem-sparing regimens for non-severe infections | Integrated with digital stewardship tools and real-time resistance dashboards | . Includes patient leaflets on antibiotic adherence | [168] |
|  |  | Malaysia  NAG | 2014 | aligns with the AMS Program and incorporates updated evidence on AMR | MOH | National | Targets resistance in Klebsiella pneumoniae, Acinetobacter baumannii, and MRSA | Recommends narrow-spectrum antibiotics (e.g., amoxicillin) for mild infections; restricts carbapenems | Integrated with antimicrobial stewardship programs (ASPs) and audits | Includes public awareness campaigns | [169] |
|  |  | PPUKM Guideline | 2012 | Interdisciplinary panel updates evidence-based guidelines | MOH | National | Addresses resistance in Staphylococcus aureus (MRSA), ESBL-producing Enterobacteriaceae | Recommends empiric therapy (e.g., piperacillin-tazobactam) for severe infections; promotes de-escalation | Includes stewardship protocols and compliance audits | Limited explicit guidance | [170] |
|  |  | MalaysiaNAG | 2008 | Based on current evidence, drug formulary, AMR patterns | MOH | National | Focuses on resistance in Streptococcus pneumoniae and Haemophilus influenzae | Recommends penicillin-based therapy for CAP; limits fluoroquinolones to severe cases | Early stewardship initiatives and formulary restrictions | Limited explicit guidance | [171] |
| **Lower Middle Income** | **Cambodia** | CPG SAT guidelines | 2016 | revision of the original practice guidelines | SHCH/HMC | International | Addresses resistance in common pathogens (e.g., E. coli, Klebsiella pneumoniae, Staphylococcus aureus) | Recommends first-line antibiotics (e.g., amoxicillin, cotrimoxazole) for common infections; discourages overuse of broad-spectrum agents | Integrated with national health programs, training for healthcare workers, and community education | Includes patient education on adherence and prevention | [172] |
|  | **Papua New Guinea** | PNG HIV Guidelines | 2019 | Evidence-based recommendations, expert consensus, data. | NdoH | National | Addresses HIV drug resistance and resistance in opportunistic pathogens (e.g., Mycobacterium tuberculosis) | Recommends standardized ART regimens, resistance testing for treatment failure, and prophylaxis for OIs | Integrated with HIV programs, healthcare worker training, and resistance monitoring | Adherence counseling for ART and infection prevention | [173] |
|  |  | PNG Pediatric STGs | 2016 | Evidence-based, expert-reviewed. | PSPG | National | Targets resistance in E. coli and Streptococcus pyogene | Recommends oral rehydration for diarrhea; reserves antibiotics for dysentery | Integration with immunization programs | Parental guidance on infection prevention | [174] |
|  |  | PNG Adult STGs | 2012 | scoping reviews, field research, epidemiological principles, comprehensive and evidence-based approach. | NDOH/WHO | National | Addresses resistance in Streptococcus pneumoniae and Haemophilus influenzae | Recommends amoxicillin as first-line for pneumonia; limits macrolide use | Training for healthcare workers in rural settings | Community education on antibiotic completion | [175] |
|  | **Philippine** | PIDS Antibiotic Guidelines | 2017 | Review of evidence-based local and international guidelines and literature. | NAGCOM | National | Addresses resistance in Salmonella typhi, Staphylococcus aureus, and Mycobacterium tuberculosis | Recommends tailored regimens for drug-resistant TB; limits carbapenems to severe infections | Includes stewardship training and diagnostic proto | Emphasizes adherence to TB treatment regimens | [176] |
|  |  | PSMID UTI Guidelines | 2015 | Evidence-based recommendations, expert consensus, and local AMR | PSMID | National | Addresses resistance in E. coli (ESBL-producing), Klebsiella pneumoniae, and Enterococcus | Uncomplicated UTI: First-line nitrofurantoin or trimethoprim-sulfamethoxazole (if local resistance <20%). Complicated UTI: Reserve fluoroquinolones/carbapenems for confirmed resistance . Culture-guided therapy: Mandate urine culture for recurrent/persistent infections. | Stewardship programs, clinician training, and prescription audits | Emphasize adherence to treatment, hydration, and hygiene practices | [177] |
|  | **Samoa** | PIOA Guidelines | 2017 | Evidence based Guideline. | PIOA | Local/Regional | Targets resistance in E. coli and Streptococcus pyogenes | Recommends prophylactic antibiotics for surgeries and culture-guided therapy for infections | Integration with immunization programs | Patient education on post-surgical care | [178] |
| **Low income** | **Tuvalu** | TST Guideline | 2010 | Evidence-based Practice. | MOH | National | Not specified | Prioritizes essential antibiotics; discourages empiric use | Integrated with national healthcare programs | Community education on adherence | [179] |
|  | **Solomon** | Pacific Islands Pediatric STGs | 2017 | Evidence-based pediatric care protocols | UNICEF, WHO | National | Targets resistance in E. coli and Streptococcus pyogenes | Recommends oral rehydration for diarrhea; reserves antibiotics for severe infections | Integration with immunization programs | Parental guidance on infection prevention | [180] |
|  |  | Solomon Islands NCD Guidelines | 2011 | evidence-based, WHO recommendations, local and international input. | MOH/MS  & WHO | National | Addresses resistance in Streptococcus pneumoniae and E. coli | Recommends rational antibiotic use for NCD-related infections | Integrated with NCD management programs | Community education on NCD prevention and management | [181] |

**References:**

1. Bonkat, G., et al., EAU guidelines on urological infections. European association of urology, 2017. **18**: p. 22-26.

2. Schoffelen, T., et al., European society of clinical microbiology and infectious diseases guidelines for antimicrobial stewardship in emergency departments (endorsed by European association of hospital pharmacists). Clinical Microbiology and Infection, 2024. **30**(11): p. 1384-1407.

3. Martin-Loeches, I., et al., ERS/ESICM/ESCMID/ALAT guidelines for the management of severe community-acquired pneumonia. Intensive care medicine, 2023. **49**(6): p. 615-632.

4. Tacconelli, E., et al., ESCMID-EUCIC clinical guidelines on decolonization of multidrug-resistant Gram-negative bacteria carriers. Clinical microbiology and infection, 2019. **25**(7): p. 807-817.

5. Polverino, E., et al., European Respiratory Society guidelines for the management of adult bronchiectasis. 2017. **50**(3).

6. Torres, A., et al. International ERS/ESICM/ESCMID/ALAT guidelines for the management of hospital-acquired pneumonia and ventilator-associated pneumonia: Guidelines for the management of hospital-acquired pneumonia (HAP)/ventilator-associated pneumonia (VAP) of the European Respiratory Society (ERS), European Society of Intensive Care Medicine (ESICM), European Society of Clinical Microbiology and Infectious Diseases (ESCMID) and Asociación Latinoamericana del Tórax (ALAT). 2017 July 24, 2024]; Available from: <https://pubmed.ncbi.nlm.nih.gov/28890434/>.

7. Van de Beek, D., et al., ESCMID guideline: diagnosis and treatment of acute bacterial meningitis. Clinical microbiology and infection, 2016. **22**: p. S37-S62.

8. Klastersky, J., et al., Management of febrile neutropaenia: ESMO clinical practice guidelines. 2016. **27**: p. v111-v118.

9. Butler, K., Manual of Childhood Infections. 2016: Oxford University Press.

10. Habib, G., et al., 2015 ESC Guidelines for the management of infective endocarditis. 2016. **69**(1).

11. Stein, R., et al., Urinary tract infections in children: EAU/ESPU guidelines. European urology, 2015. **67**(3): p. 546-558.

12. Debast, S.B., et al., European Society of Clinical Microbiology and Infectious Diseases: update of the treatment guidance document for Clostridium difficile infection. Clinical microbiology and infection, 2014. **20**: p. 1-26.

13. Pelucchi, C., et al., Guideline for the management of acute sore throat: ESCMID Sore Throat Guideline Group. 2012. **18**: p. 1-28.

14. Health, M.o. Standard Treatment Guidelines for Seychelles 2003; Available from: <https://extranet.who.int/ncdccs/Data/SYC_D1_Standard%20Treatment%20Guidlines-Seychelles.pdf>.

15. Health, M.o. Namibia Standard Treatment Guidelines. 2011; Available from: <http://www.man.com.na/files/news/1501069447namibia-standard-treatment-guidelines.pdf>.

16. health, M.o., Eswatini Standard Paediatric Treatment Guidelines and Essential Medicines List of Common Medical Conditions in the Kingdom of Eswatini

2020.

17. Health, M.o. Standard Treatment Gideline and EML,Eswatini. 2012; Available from: <https://www.medbox.org/document/standard-treatment-guidelines-and-essential-medicines-list-of-common-medical-conditions-in-the-kingdom-of-swaziland>.

18. Health, G.m.o. Provisional Standard Treatment Guidelines for Novel Coronavirus Infection COVID - 19 Guidelines for Ghana. 2020 June 22, 2024]; Available from: <https://www.moh.gov.gh/wp-content/uploads/2016/02/COVID-19-STG-JUNE-2020-1.pdf>.

19. Health, M.o. Ghana National Drugs Programme (GNDP) Standard Treatment Guidelines 2017. 2017 June 25, 2024]; 7th edition]. Available from: <https://www.moh.gov.gh/wp-content/uploads/2020/07/GHANA-STG-2017-1.pdf>.

20. Health, M.o. National Antimicrobial Antimicrobial Stewardship Guidelines For Health care Settings in Kenya

2020 June 29, 2024]; Available from: <http://guidelines.health.go.ke:8000/media/National_Antimicrobial_Stewardship_Guidelines_for_Health_Care_Settings_in_Kenya_-_March_2020.pdf>.

21. Gitaka, J., et al., Combating antibiotic resistance using guidelines and enhanced stewardship in Kenya: a protocol for an implementation science approach. 2020. **10**(3): p. e030823.

22. Agweyu, A., N. Opiyo, and M.J.B.p. English, Experience developing national evidence-based clinical guidelines for childhood pneumonia in a low-income setting-making the GRADE? 2012. **12**: p. 1-12.

23. Oladele, R., et al., Antibiotic Guidelines for Critically Ill Patients in Nigeria. West African Journal of Medicine, 2023. **40**(9): p. 962-972.

24. Ministry of Health. Nigeria Standard Treatment Guidelines. 2016 July 04, 2024]; Available from: <https://www.medbox.org/document/nigeria-standard-treatment-guidelines#GO>.

25. Health, M.o. Standard Treatment Guideline and EML for Tanzania mainland 2021 [cited 6th Edition; Available from: <https://medicine.st-andrews.ac.uk/igh/wp-content/uploads/sites/44/2022/01/STG-NEMLIT-2021.pdf>.

26. Ministry of Health, T. Policy Guideline for Implementing Antimicrobial Stewardship. 2020 June 28, 2024]; Available from: <https://www.moh.go.tz/storage/app/uploads/public/657/6b5/58e/6576b558e32b1485010391.pdf>.

27. Health, M.o. Standard Treatment Guideline and NEML, Tanzania Mainland 2017 June 26, 2024]; Available from: <https://hssrc.tamisemi.go.tz/storage/app/uploads/public/5ab/e9b/b21/5abe9bb216267130384889.pdf>.

28. MOH. Essential newborn care Guidelines,Zambia. 2014; Available from: <https://www.afro.who.int/sites/default/files/2019-06/ESSENTIAL%20NEWBORN%20CARE%20GUIDELINES%202014.pdf>.

29. Ministry of Health, E. Standard Treatment Guidelines for Primary Hospital. 2014 July 05, 2024]; Available from: <https://siapsprogram.org/wp-content/uploads/2014/12/Primary-Hospital-Final.pdf>.

30. MOH. Malawi Standard Treatment Guidelines (MSTG). 2015(5th Edition). 2015; Available from: <https://extranet.who.int/ncdccs/Data/MWI_D1_Malawi-Standard-Treatment-Guidelines-Essential-Medicines-List-2015.pdf>.

31. MOH. Guide to Common Infections in Madagascar 2020; Available from: <https://www.fondation-merieux.org/en/news/the-first-guide-to-common-infections-in-madagascar-to-improve-antibiotic-use/>.

32. SPIM. Antibiotika Tsara, antibiotic Guide Madagascar. 2018; Available from: <https://smartbiotic.ai/product/antibiotika-tsara-madagaskar/>.

33. Health, M.o. Rwanda STG, The guideline for the use of Antibiotics in Rwanda. 2022 May 11, 2024]; Available from: <https://www.moh.gov.rw/index.php?eID=dumpFile&t=f&f=92525&token=65bb1585f37424835c9eeb4c9ad4747b38a80514>.

34. Ministry of Health, R. COVID-19 CLINICAL MANAGEMENT GUIDELINES. 2020; Available from: <https://www.rbc.gov.rw/fileadmin/user_upload/guide/Guidelines/COVID-19%20Clinical%20Managment%20guidelines.pdf>.

35. Department of Health, S.A. Guidelines for the Prevention and Containment of Antimicrobial Resistance in South African Hospitals. 2023 June 16, 2024]; Available from: <https://knowledgehub.health.gov.za/system/files/elibdownloads/2023-04/Guidelines%2520for%2520the%2520prevention%2520and%2520containment%2520of%2520AMR%2520in%2520SA%2520hospitals.pdf>.

36. South Africa, C. African Antibiotic Treatment Guidelines for Common Bacterial Infections and Syndromes—Recommended Antibiotic Treatments in Neonatal and Pediatric Patients. 2022; Available from: <https://africaguidelines.onehealthtrust.org/wp-content/uploads/2021/11/Guidelines_Adults_Peds_English.pdf>.

37. GUIDELINES TO ANTIMICROBIAL THERAPY AND THE LABORATORY DIAGNOSIS OF INFECTIOUS DISEASES. 2017; Available from: <https://www.ampath.co.za/storage/416/2017-antibiotic-guide---june-2017---toc.pdf>.

38. Boyles, T.H., et al., South African guideline for the management of community-acquired pneumonia in adults. Journal of Thoracic Disease, 2017. **9**(6): p. 1469.

39. SAASP. A POCKET GUIDE TO ANTIBIOTIC PRESCRIBING FOR ADULTS IN SOUTH AFRICA, . 2014; Available from: <https://sahivsoc.org/Files/Guide%20to%20Antibiotice%20prescribing%20for%20adults%20in%20SA_2014%20(Oct%202014).pdf>.

40. Health, M.o. Uganda Clinical Guidelines

2023 June 29, 2024]; Available from: <https://library.health.go.ug/sites/default/files/resources/Uganda%20Clinical%20Guidelines%202023.pdf>.

41. Health, M.o. National Guidelines for Antimicrobial Consumption and Use Surveillance in Human Health Published by the Ministry of Health, Republic of Uganda. 2020; Available from: <https://cphl.go.ug/web/sites/default/files/2024-10/National%20Guidelines%20for%20AMCU%20in%20HH%2017.06.2020.pdf>.

42. 7th Essential Medicines List and Standard Treatment Guidelines for Zimbabwe. 2015.

43. NHRA, B. Treatment Guidelines and Pathways Jan 2022 [cited 12; Available from: <https://www.nhra.bh/MediaHandler/GenericHandler/documents/Announcements/COVID-19/General/Bahrain%20Treatment%20Protocol%20(V12.0)%20Jan%202022%20(English).pdf>.

44. Health, M.o. National Antimicrobial Guidelines , Oman. 2016; Available from: <https://www.who.int/publications/m/item/oman--national-formulary-2016-%28english%29?utm_source>.

45. Health, M.o. National Surgical Antibiotic Prophylaxis Guideline. 2015; Available from: <https://moh.gov.om/documents/236878/0/national+surgical+antimicrobial+prophylaxis/dd57462f-2f8b-47c6-b78b-f2821ad17fc9>.

46. Ibrahim, W. Pneumnia Guidelines Development Group. The diagnosis and management of community acquired pneumonia. National Clinical Guidelines 2016 July 02, 2024]; Available from: <https://www.researchgate.net/publication/312040120_Pneumnia_Guidelines_Development_Group_The_diagnosis_and_management_of_community_acquired_pneumonia_National_Clinical_Guidelines_-_Qatar_httpswwwmophgovqaclinical-guidelines>.

47. Ministry of Health, S.A. UTI infection managment protocol. July 02, 2024]; Available from: <https://www.moh.gov.sa/Ministry/MediaCenter/Publications/Documents/Urinary-Tract-Infection-management-protocol.pdf>.

48. Ministry of Health, S.A. Lower Respiratory tract infection Protocol. 2020 July 04, 2024]; Available from: <https://www.moh.gov.sa/Ministry/MediaCenter/Publications/Documents/Lower-Respiratory-Tract-Infections-Management-Protocol.pdf>.

49. Ministry of Health, S.A. National Antimicrobial Therapy Guidelines for Community and Hospital Acquired Infections in Adults. 2018 Available from: <https://saudithoracicsociety.org/wp-content/uploads/2019/11/National-Antimicrobial-Guidelines-for-Community-and-Hospital-Acquired-Infections-in-Adults.pdf>.

50. Alzomor, O., et al., Management of community-acquired pneumonia in infants and children: Clinical practice guidelines endorsed by the Saudi Pediatric Infectious Diseases Society. 2017. **4**(4): p. 153-158.

51. Ministry of Health, U. National Guidelines on the Empiric Antibiotic Treatment of Intra-abdominal Infections, UAE guideline. 2022; Available from: <https://mohap.gov.ae/documents/20117/626089/61c6912c-df03-4bc6-93c0-e2837bd093c2/1208b629-2260-45c4-7bc0-5d9fb6f576e4>.

52. EDA/NARC. NATIONAL GUIDELINES FOR PREAUTHORIZATION OF RESTRICTED ANTIMICROBIALS IN HOSPITALS,Egypt. Dec 2022 4]; Available from: <https://www.edaegypt.gov.eg/media/of1bgvnf/national-guidelines-for-preauthorization-of-restricted-ab-in-hospitals-issue-4.pdf>.

53. Moghnieh, R., et al., The Lebanese Society for Infectious Diseases and Clinical Microbiology (LSIDCM) guidelines for adult community-acquired pneumonia (CAP) in Lebanon. 2014. **103**(1006): p. 1-8.

54. BIH. Antibiotic Guideline 2023, Bahria International Hospital, Lahore. 2023; Available from: <https://bahriainternationalhospital.com/5952-2/>.

55. DRAP. GUIDELINES ON RESPONSIBLE USE OF ANTIMICROBIALS IN HUMAN HEALTH. 2022 June 17, 2024]; Available from: <https://www.dra.gov.pk/wp-content/uploads/2022/02/Guidelines-Responsible-Use-of-Antimicrobials-1.pdf>.

56. MMIDSP. Typhoid Management Guidelines ,Pakistan. 2022; Available from: <https://www.mmidsp.com/wp-content/uploads/2023/03/Typhoid-Management-Guideline-2022-Jun-22.pdf>.

57. Society, P.C. Guidelines for Managment of COPD,Pakistan. 2020; Available from: <https://www.pakistanchestsociety.pk/wp-content/uploads/2020/09/COPD-Update.pdf>.

58. PARN, I.a. IDSP & PARNGuidelines 2019; Available from: <https://www.mmidsp.com/wp-content/uploads/2019/09/Guidelines-for-Antimicrobial-Use-2.pdf>.

59. Bokhari, N., et al. Pakistan Chest Society. Guidelines for the management of community acquired pneumonia in adults. 2017; Available from: <https://www.pakistanchestsociety.pk/wp-content/uploads/79_archives.pdf>.

60. Hashmi, M., et al., Developing local guidelines for management of sepsis in adults: Sepsis Guidelines for Pakistan (SGP). 2015. **19**(2): p. 196.

61. Health, M.o. Somalia National Treatment Guidelines in line with the Essential Package of Health Services. 2015; Available from: <https://moh.gov.so/en/wp-content/uploads/2020/07/Primary-Health-Unit-I-Somali-Treatment-Guidelines-2015.pdf>.

62. Beneš, J., et al., Updated Czech guidelines for the treatment of patients with colitis due to Clostridioides difficile. 2022. **28**(3): p. 77-94.

63. Authority, D.H.a.m. Guidelines on prescribing Antibiotics ,Denmark. 2013; Available from: <https://www.sundhedsstyrelsen.dk/-/media/Udgivelser/2013/Publ2013/Guidelines-on-prescribing-antibiotics.ashx>.

64. Mauritz, M.D., et al., Clinical recommendations for the inpatient management of lower respiratory tract infections in children and adolescents with severe neurological impairment in Germany. 2024: p. 1-13.

65. Rose, M., et al., Guidelines for the Management of Community Acquired Pneumonia in Children and Adolescents (Pediatric Community Acquired Pneumonia, pCAP)-Issued under the Responsibility of the German Society for Pediatric Infectious Diseases (DGPI) and the German Society for Pediatric Pulmonology (GPP). National Library of Medicine, 2020. **74**(8): p. 515-544.

66. De With, K., et al., Strategies to enhance rational use of antibiotics in hospital: a guideline by the German Society for Infectious Diseases. 2016. **44**: p. 395-439.

67. Windfuhr, J.P., et al., Clinical practice guideline: tonsillitis I. Diagnostics and nonsurgical management. European Archives of Oto-Rhino-Laryngology, 2016. **273**: p. 973-987.

68. Höffken, G., et al., Epidemiology, diagnosis, antimicrobial therapy and management of community-acquired pneumonia and lower respiratory tract infections in adults. Guidelines of the Paul-Ehrlich-Society for Chemotherapy, the German Respiratory Society, the German Society for Infectiology and the Competence Network CAPNETZ Germany. National Library of medicine, 2009. **63**(10): p. e1-e68.

69. CHI. Antimicrobial Guidelines ,Ireland

2020 August 02, 2024]; Available from: <https://www.iaem.ie/wp-content/uploads/2021/02/Antimicrobial-Guidelines-2020.pdf>.

70. Barry, E., et al., PIPc study: development of indicators of potentially inappropriate prescribing in children (PIPc) in primary care using a modified Delphi technique. BMJ, 2016. **6**(9): p. e012079.

71. Autore, G., et al. Antibiotic prophylaxis for the prevention of urinary tract infections in children: guideline and recommendations from the Emilia-Romagna Pediatric Urinary Tract Infections (UTI-Ped-ER) study group,Italy. 2023 [cited 12 6]; 1040]. Available from: <https://pubmed.ncbi.nlm.nih.gov/37370359/>.

72. Bassetti, M., et al., Clinical management of adult patients with COVID-19 outside intensive care units: guidelines from the Italian Society of Anti-Infective Therapy (SITA) and the Italian Society of Pulmonology (SIP). 2021. **10**(4): p. 1837-1885.

73. Guideline, S. Management of Community-Acquired Pneumonia in Adults: the 2024 Practice Guideline, Netherland. 2024 June 20, 2024]; Available from: <https://swab.nl/en/exec/file/download/300>.

74. Akhloufi, H., et al., The development and implementation of a guideline-based clinical decision support system to improve empirical antibiotic prescribing. 2022. **22**(1): p. 127.

75. Aisanov, Z., et al., Russian guidelines for the management of COPD: algorithm of pharmacologic treatment. PMC, 2018: p. 183-187.

76. Guidance, S. Guidance on Prevention and Control of Clostridium difficile Infection (CDI) in Care Settings in Scotland. 2014 June 16, 2024]; Available from: <https://citeseerx.ist.psu.edu/document?repid=rep1&type=pdf&doi=64eb111d42cbf505519fb9542e77b60ef9ace075>.

77. SMA. SLOVENIAN GUIDELINES ON ANTIBIOTIC PROPHYLAXIS APPLICATION IN NEUROSURGICAL OPERATIONS. 2014 September 10, 2024]; Available from: <https://vestnik.szd.si/index.php/ZdravVest/article/view/1017/915>.

78. MSF. Clinical guidelines - Diagnosis and treatment manual,Spain. 2024; Available from: <https://medicalguidelines.msf.org/sites/default/files/pdf/guideline-170-en.pdf>.

79. Gisbert, J.P., et al., V Spanish Consensus Conference on Helicobacter pylori infection treatment. Gastroenterología y Hepatología, 2022. **45**(5): p. 392-417.

80. Spindler, C., et al., Swedish guidelines on the management of community-acquired pneumonia in immunocompetent adults—Swedish Society of Infectious Diseases 2012. National Library of Medicine, 2012. **44**(12): p. 885-902.

81. Paioni, P., et al., Swiss recommendations on perioperative antimicrobial prophylaxis in children. National Library of Medicine, 2022. **152**(3738): p. w30230-w30230.

82. Nagassar, R.P. Recommendations for antibiotic prescriptions for upper respiratory symptoms in children in Trinidad and Tobago: GRADE-ADOLOPMENT APPROACH. 2020; Available from: <https://www.caribbeanmedicaljournal.org/2020/05/09/recommendations-for-antibiotic-prescriptions-for-upper-respiratory-symptoms-in-children-in-trinidad-and-tobago-grade-adolopmen>.

83. HCCM, G. Greater Manchester Antimicrobial Guidelines. 2024 June 20, 2024]; Available from: <https://gmmmg.nhs.uk/wp-content/uploads/2024/02/GM-Antimicrobial-guidelines-January-2024-v15.pdf>.

84. ICB, B. Management of Infection Guidance for Primary Care,UK. 2024 September 24, 2024]; Available from: <https://bswtogether.org.uk/medicines/wp-content/uploads/sites/3/2025/01/FULL-antibiotics-guidance-update-Dec-2024-recurrent-UTI-update.pdf>.

85. NICE. Pneumonia in adults: diagnosis and management NICE Guideline. 2023; Available from: <https://www.nice.org.uk/guidance/cg191>.

86. Smith, D., et al., British Thoracic Society guideline for the use of long-term macrolides in adults with respiratory disease. BMJ open respiratory research, 2020. **7**(1): p. e000489.

87. Chirwa, M., et al., United Kingdom British association for sexual health and HIV national guideline for the management of epididymo-orchitis, 2020. National Library of Medicine, 2021. **32**(10): p. 884-895.

88. FDS, F.U.a. Antimicrobial Prescribing in Dentistry – Good Practice Guidelines,UK. 2020; Available from: <https://www.rcseng.ac.uk/dental-faculties/fds/faculty/news/archive/antimicrobial-prescribing-guidelines/>.

89. NICE. Pneumonia (hospital-acquired): antimicrobial prescribing,UK. 2019 July 08, 2024]; Available from: <https://www.nice.org.uk/guidance/ng139/resources/pneumonia-hospitalacquired-antimicrobial-prescribing-pdf-66141727749061?utm>.

90. NICE. NICE GUIDELINE Pneumonia (community acquired): antimicrobial prescribing,UK. 2019; Available from: <https://www.nice.org.uk/guidance/ng138>.

91. Gould, F.K., et al. Guidelines for the diagnosis and antibiotic treatment of endocarditis in adults: a report of the Working Party of the British Society for Antimicrobial Chemotherapy. National Library of Medicine 2012 [cited 67 2]; 269-289]. Available from: <https://pubmed.ncbi.nlm.nih.gov/22086858/>.

92. Stewardship, G.P.A. Georgia Pediatric Antibiotic Stewardship Guidelines Guidelines,. 2023; Available from: <https://www.gpas-online.org/guidelines/>.

93. Desrosiers, M., et al., Canadian clinical practice guidelines for acute and chronic rhinosinusitis. Allergy, Asthma & Clinical Immunology, 2011. **7**: p. 1-38.

94. Barton, M., et al., Guidelines for the prevention and management of community‐associated methicillin‐resistant Staphylococcus aureus: a perspective for Canadian health care practitioners. Canadian Journal of Infectious Diseases and Medical Microbiology, 2006. **17**: p. 4C-24C.

95. Tamma, P.D., et al., Infectious Diseases Society of America 2024 guidance on the treatment of antimicrobial-resistant gram-negative infections. Clinical infectious diseases, 2024: p. ciae403.

96. Guide, S. Pneumonia Ventilator associated 2024. 2024 June 09, 2024]; Available from: <https://web.sanfordguide.com/en/sanford-guide-online/disease-clinical-condition/pneumonia-ventilator-associated>.

97. CDC. Adult Antibiotic Prescribing Guidelines. 2023 June 14, 2024]; Available from: <https://cha.com/wp-content/uploads/2018/04/Telligen-Version-CDC-Outpatient-Antibiotic-Treatment-Guidelines.pdf>.

98. Chaudhuri, D., et al., 2024 Focused Update: Guidelines on Use of Corticosteroids in Sepsis, Acute Respiratory Distress Syndrome, and Community-Acquired Pneumonia. National Library of Medicine, 2024. **52**(5): p. e219-e233.

99. Senneville, É., et al., IWGDF/IDSA guidelines on the diagnosis and treatment of diabetes‐related foot infections (IWGDF/IDSA 2023). Diabetes/metabolism research and reviews, 2024. **40**(3): p. e3687.

100. (MHHA), M.H.H.A. Guidelines for Treatmentof Urinary Tract Infections. 2021 June 01, 2024]; Available from: <https://www.mi-hms.org/sites/default/files/UTI%20Guideline-6.9.21.pdf>.

101. Book, R. Red Book (2021): Report of the Committee on Infectious Diseases. 2021 June 05, 2024]; Available from: <https://publications.aap.org/aapbooks/book/663/Red-Book-2021-Report-of-the-Committee-on?autologincheck=redirected>.

102. Lipsky, B.A., et al., Guidelines on the diagnosis and treatment of foot infection in persons with diabetes (IWGDF 2019 update). Diabetes/metabolism research and reviews, 2020. **36**: p. e3280.

103. Jackson, C.D., D.C. Burroughs‐Ray, and N.A. Summers, Clinical guideline highlights for the hospitalist: 2019 American Thoracic Society/Infectious Diseases Society of America update on community‐acquired pneumonia. Journal of Hospital Medicine, 2020. **15**(12): p. 743-745.

104. Norris, A.H., et al., 2018 Infectious Diseases Society of America clinical practice guideline for the management of outpatient parenteral antimicrobial therapy. Clinical infectious diseases, 2019. **68**(1): p. e1-e35.

105. Randel, A., Infectious diarrhea: IDSA updates guidelines for diagnosis and management. American Family Physician, 2018. **97**(10): p. 676-677.

106. McDonald, L.C., et al., Clinical practice guidelines for Clostridium difficile infection in adults and children: 2017 update by the Infectious Diseases Society of America (IDSA) and Society for Healthcare Epidemiology of America (SHEA). Clinical infectious diseases, 2018. **66**(7): p. e1-e48.

107. CDC, U. ADULT ANTIBIOTIC PRESCRIBING GUIDELINES. 2017 une 13, 2024]; Available from: <https://www.health.ny.gov/publications/1174_8.5x11.pdf>.

108. Rosenfeld, R.M., et al., Clinical practice guideline: otitis media with effusion (update). Otolaryngology–Head and Neck Surgery, 2016. **154**(1_suppl): p. S1-S41.

109. Riddle, M.S., H.L. DuPont, and B.A. Connor, ACG clinical guideline: diagnosis, treatment, and prevention of acute diarrheal infections in adults. Official journal of the American College of Gastroenterology| ACG, 2016. **111**(5): p. 602-622.

110. Bredemeyer, M.J.A.F.P. ACP/CDC provide guidelines on the use of antibiotics for acute respiratory tract infection. 2016 June 06, 2024].

111. Rosenfeld, R.M., et al., Clinical practice guideline (update): adult sinusitis. Otolaryngology–Head and Neck Surgery, 2015. **152**(2_suppl): p. S1-S39.

112. Stevens, D.L., et al., Practice guidelines for the diagnosis and management of skin and soft tissue infections: 2014 update by the Infectious Diseases Society of America. Clinical infectious diseases, 2014. **59**(2): p. e10-e52.

113. Bratzler, D.W., et al., Clinical practice guidelines for antimicrobial prophylaxis in surgery. 2013. **14**(1): p. 73-156.

114. Chow, A.W., et al., IDSA clinical practice guideline for acute bacterial rhinosinusitis in children and adults. Clinical infectious diseases, 2012. **54**(8): p. e72-e112.

115. Roberts, K.B., S.C.o.Q.I. Subcommittee on Urinary Tract Infection, and M.J. Pediatrics, Urinary tract infection: clinical practice guideline for the diagnosis and management of the initial UTI in febrile infants and children 2 to 24 months. 2011, American Academy of Pediatrics Elk Grove Village, IL, USA. p. 595-610.

116. Bradley, J.S., et al., The management of community-acquired pneumonia in infants and children older than 3 months of age: clinical practice guidelines by the Pediatric Infectious Diseases Society and the Infectious Diseases Society of America. 2011. **53**(7): p. e25-e76.

117. Nishimura, R.A., et al., ACC/AHA 2008 guideline update on valvular heart disease: focused update on infective endocarditis: a report of the American College of Cardiology/American Heart Association Task Force on Practice Guidelines: endorsed by the Society of Cardiovascular Anesthesiologists, Society for Cardiovascular Angiography and Interventions, and Society of Thoracic Surgeons. 2008. **118**(8): p. 887-896.

118. Rosenfeld, R.M., et al., Clinical practice guideline: adult sinusitis. 2007. **137**(3): p. S1-S31.

119. Braman, S.S.J.C., Chronic cough due to acute bronchitis: ACCP evidence-based clinical practice guidelines. 2006. **129**(1): p. 95S-103S.

120. Bisno, A.L., et al., Practice guidelines for the diagnosis and management of group A streptococcal pharyngitis. Clinical infectious diseases, 2002. **35**(2): p. 113-125.

121. Guerrant, R.L., et al., Practice guidelines for the management of infectious diarrhea. Clinical infectious diseases, 2001. **32**(3): p. 331-351.

122. Pediatrics, A.A.o., Clinical practice guideline: management of sinusitis. Pediatrics, 2001. **108**(3): p. 798-808.

123. Bartlett, J.G., et al., Community-acquired pneumonia in adults: guidelines for management. Clinical infectious diseases, 1998. **26**(4): p. 811-838.

124. Corrêa, R.d.A., et al., Brazilian guidelines for community-acquired pneumonia in immunocompetent adults-2009. Jornal Brasileiro de Pneumologia, 2009. **35**: p. 574-601.

125. Nascimento-Carvalho, C.M.S.-M. Recommendation of the Brazilian Society of Pediatrics for antibiotic therapy in children and adolescents with community-acquired pneumonia. 2004 July 05, 2024]; 380-387]. Available from: <https://pubmed.ncbi.nlm.nih.gov/15272984/>.

126. Health, M.o. Standard Treatment Guidelines for Primary Health Care,Guyana. 2015 [cited 2nd Edition; Available from: <https://extranet.who.int/ncdccs/Data/GUY_D1_Guyana%20STG%202015_online.pdf>.

127. Young Peart S, T.C.R., Abel C,. Management Guidelines for Urinary Tract Infections in Jamaican Children 2018. 2018; Available from: <https://kidneykidsja.com/wp-content/uploads/Jamaican-2018-guidelines-for-UTI-investigation-in-kids.pdf>.

128. health, M.o. National Antimicrobial Stewardship program (NAMSP),Maldives. 2020; Available from: <https://health.gov.mv/storage/uploads/eJYk04qR/yc1ousrq.pdf>.

129. Kawamatawong, T., et al., Guidelines for the management of asthma in adults: evidence and recommendations. Asian Pacific Journal of Allergy and Immunology, 2022. **40**(1): p. 1-21.

130. BSMMU. BSMMU Guidelines 2023 June 11, 2024]; Available from: <https://forms.bsmmu.ac.bd/antibiotic_guideline/list.html>.

131. Health, M.o. Standard Treatment Guidelines (STG) on Antibiotic Use in Common Infectious Diseases of Bangladesh

2021 August 03, 2024]; Available from:

<https://amr.cdc.gov.bd/wp-content/uploads/2018/10/STG-guideline-for-antimicrobial-use-version-1.0-date-1-december21.pdf>.

132. HOSPITAL, B. Antibiotic Protocol for BIRDEM General Hospital 2021 July 01, 2024]; Available from: <https://file-dhaka.portal.gov.bd/uploads/c1124d76-9c11-4f4a-98ba-020b19f169d5/650/f1e/ae2/650f1eae254a3562310116.pdf>.

133. National-Antibiotic-Guideline. 2018.

134. Valadares, H.N.G. ANTIBIOTIC GUIDELINES 2016 August 11, 2024]; Available from: <https://extranet.who.int/uhcpartnership/sites/default/files/reports/Timor-Leste-Antibiotic-Guidelines.pdf>.

135. Kulkarni, A.P., et al., Indian antimicrobial prescription guidelines in critically ill immunocompromised patients. Indian Journal of Critical Care Medicine: Peer-reviewed, Official Publication of Indian Society of Critical Care Medicine, 2019. **23**(Suppl 1): p. S64.

136. Khilnani, G., et al., Guidelines for antibiotic prescription in intensive care unit. Indian journal of critical care medicine: peer-reviewed, official publication of Indian Society of Critical Care Medicine, 2019. **23**(Suppl 1): p. S1.

137. Control, N.C.f.D. National treatment guidelines for antimicrobial use in infectious diseases, India. 2016; Available from: <https://ncdc.mohfw.gov.in/wp-content/uploads/2024/04/File622.pdf>.

138. WHO, G. North Okkalapa General and teaching Hospital Antibiotic Guidelines. Myanmar. 2019 1st Edition]; Available from: <https://medbox.org/pdf/62fb4f0042d0cad2d90d2495>.

139. Health, M.o. empirical and prophylactic use of antimicrobials national guidelines ,Srilanak. 2016; Available from: <https://slmicrobiology.lk/empirical-and-prophylactic-use-of-antimicrobials-national-guidelines-sri-lanka-2024/>.

140. Association, S.M. SLMA Guidelines on the use of Antimicrobial Agents,Srilanka. 2014; Available from: <https://slma.lk/wp-content/uploads/2014/12/Guidelines-on-the-use-of-antimicrobial-agents.pdf>.

141. TGL. Therapeutic Guidelines Antibiotic prescribing in primary care: . 2024 July 24, 2024]; Available from: <https://ccmsfiles.tg.org.au/s3/PDFs/GPSummary_v15.pdf>.

142. CHQHospital. Children’s Health Queensland Paediatric Antibiocard: Empirical Antibiotic Guidelines. 2022 July 01, 2024]; Available from: <https://www.childrens.health.qld.gov.au/__data/assets/pdf_file/0037/176878/Antibiocard.pdf>.

143. Govt south Australia. Surgical Antimicrobial Prophylaxis Guidelines. Dec 2021 June 18, 2024]; Available from: <https://www.sahealth.sa.gov.au/wps/wcm/connect/public+content/sa+health+internet/clinical+resources/clinical+programs+and+practice+guidelines/medicines+and+drugs/antimicrobial+guidelines/antimicrobial+guidelines>.

144. McTaggart, S., et al., KHA-CARI guideline: Diagnosis and treatment of urinary tract infection in children. Nephrology, 2015. **20**(2).

145. mMinistry of health, B.D. NATIONAL BEST PRACTICEGUIDANCE FOR GOOD ANTIBIOTICPRESCRIBING PRACTICE (GAPP). 2019; Available from: <https://moh.gov.bn/wp-content/uploads/2024/10/MOH-GAPP-Booklet.pdf>.

146. Ministry of Health, C.I. Guidelines for empiric and targeted antibiotic treatment, prophylaxis, dosing and allergies. 2023; Available from: <https://www.health.gov.ck/wp-content/uploads/2024/01/Cook-Islands-Handbook-Sep23_FINAL-digital.pdf>.

147. Ihara, E., et al., Evidence-based clinical guidelines for chronic diarrhea 2023. Digestion, 2024. **105**(6): p. 480-497.

148. Ohge, H., et al., The Japan Society for Surgical Infection: guidelines for the prevention, detection, and management of gastroenterological surgical site infection, 2018. 2021. **51**: p. 1-31.

149. Ministry of Health. Manual of Antimicrobial Stewardship,Japan. June 2017 Ist Edition]; Available from: <https://www.mhlw.go.jp/file/06-Seisakujouhou-10900000-Kenkoukyoku/0000193504.pdf>.

150. Miyashita, N., T. Matsushima, and M. Oka, The JRS guidelines for the management of community-acquired pneumonia in adults: an update and new recommendations. Internal Medicine, 2006. **45**(7): p. 419-428.

151. Kim, Y.J., et al., Guideline for the antibiotic use in acute gastroenteritis. 2019. **51**(2): p. 217-243.

152. Kang, C.-I., et al., Clinical practice guidelines for the antibiotic treatment of community-acquired urinary tract infections. Infection & chemotherapy, 2018. **50**(1): p. 67.

153. Lee, M.S., et al., Guideline for antibiotic use in adults with community-acquired pneumonia. Infection & chemotherapy, 2018. **50**(2): p. 160.

154. Yoon, Y.K., et al., Guidelines for the antibiotic use in adults with acute upper respiratory tract infections. Infection & chemotherapy, 2017. **49**(4): p. 326-352.

155. Chemotherapy, K.S.f., K.S.o.I. Diseases, and K.O. Association, Clinical guidelines for the antimicrobial treatment of bone and joint infections in Korea. Infection & Chemotherapy, 2014. **46**(2): p. 125-138.

156. bpacnz Primary Care Antibiotic Guide May 2024; Available from: <https://bpac.org.nz/antibiotics/guide.aspx>.

157. Zealand, b.N. The bpacnz antibiotic guide. [cited 2017 Edition September 02, 2024]; Available from: <https://bpac.org.nz/2017/docs/abguide.pd>.

158. ANZPID-ASAP. ANZPID-ASAP Guidelines for Antibiotic Duration and IV-Oral Switch in Children, Newzealand. 2016; Available from: <https://starship.org.nz/search/?k=antibiotic%20guidelines&m_us=Health%20Professional>.

159. Chung, W.T.G., et al., National surgical antibiotic prophylaxis guideline in Singapore. 2022. **51**(11): p. 695-711.

160. Sy, C.L., et al., Recommendations and guidelines for the treatment of infections due to multidrug resistant organisms,Taiwan. 2022. **55**(3): p. 359-386.

161. Chou, Y.-H., et al., Taiwanese recommendations for antimicrobial prophylaxis in urological surgery. 2011. **22**(2): p. 63-69.

162. Lee, P.I., et al., Guidelines for the management of community-acquired pneumonia in children. Zhonghua Minguo xiao er ke yi xue hui za zhi [Journal]. Zhonghua Minguo xiao er ke yi xue hui, 2007. **48**(4): p. 167-180.

163. China, I.D.S.o.t.R.o. and T.S. Association, Guidelines for the use of prophylactic antibiotics in surgery in Taiwan. Journal of microbiology, immunology, and infection= Wei mian yu gan ran za zhi, 2004. **37**(1): p. 71-74.

164. China, I.D.S.o.t.R.o., Guidelines for antimicrobial therapy of urinary tract infections in Taiwan. Journal of microbiology, immunology, and infection= Wei mian yu gan ran za zhi, 2000. **33**(4): p. 271-272.

165. Shi, Y., et al. Chinese guidelines for the diagnosis and treatment of hospital-acquired pneumonia and ventilator-associated pneumonia in adults (2018 Edition). 2019 July 23, 2024]; 2581]. Available from: <https://pubmed.ncbi.nlm.nih.gov/31372297/>.

166. Cao, B., et al., Diagnosis and treatment of community‐acquired pneumonia in adults: 2016 clinical practice guidelines by the Chinese Thoracic Society, Chinese Medical Association. The clinical respiratory journal, 2018. **12**(4): p. 1320-1360.

167. Health, M.o. Fiji Antibiotic Guidelines

2019; Available from: <https://ohpl.com.fj/publication/fiji-antibiotic-guidelines-4th-edition/>.

168. Ministry of Health, M. National Antimicrobial Guideline (NAG) 2024 July 06, 2024]; Available from: <https://sites.google.com/moh.gov.my/nag>.

169. Health, M.o. NATIONAL ANTIBIOTIC GUIDELINE 2014; Available from: <https://pharmacy.moh.gov.my/sites/default/files/document-upload/national-antibiotic-guideline-2014-full-versionjun2015_1.pdf>.

170. PPUKM. PPUKM Anti-Infective Guideline, Malaysia. 2012; Available from: <https://hctm.ukm.my/farmasi/wp-content/uploads/2020/08/Anti-Infective-Guideline-2012-2MB.pdf>.

171. NATIONAL ANTIBIOTIC GUIDELINE 2008; Available from: <https://www.moh.gov.my/moh/resources/auto%20download%20images/589d720fe09a1.pdf>.

172. SHCH/HMC. Clinical Practice Guidelines,Standard antibiotic treatment guidelines. 2016 July 22, 2024]; Available from: <https://niph.org.kh/niph/uploads/library/pdf/GL061_CPG_AB_SHCH_V2_0.pdf>.

173. Health, N.d.o.o. Papua New Guinea National Guidelines for HIV Care and Treatment 2019; Available from: <https://www.aidsdatahub.org/sites/default/files/resource/papua-new-guinea-national-guidelines-hiv-care-and-treatment-2019.pdf>.

174. PSPG. Standard Treatment for Common Illnesses of Children in Papua New Guinea. 2016 [cited 10th Edition Available from: <https://pngpaediatricsociety.org/wp-content/uploads/2016/11/PNG-Standard-Treatment-Book-10th-edition-2016.pdf>.

175. NDOH/WHO. Standard Treatment Guidelines for Adults in Papua New Guinea

2012; Available from: <https://extranet.who.int/ncdccs/Data/PNG_D1_Standard-Treatment-Guidelines-for-Common-Illness-of-Adults-in-PNG.pdf>.

176. NAGCOM. National Antibiotic Guideline 2017 , Philippine. 2017; Available from: <http://www.pidsphil.org/home/wp-content/uploads/2018/09/National-Antibiotic-Guidlines-Dr.-Carmina-Delos-Reyes.pdf>.

177. PSMID. Philippine Clinical Practice Guidelines on the Diagnosis and Management of Urinary Tract Infections in Adults 2015 Update: Part 2 Asymptomatic Bacteriuria in Adults, Recurrent Urinary Tract Infection, and Complicated Urinary Tract Infection. 2015 July 16, 2024]; Available from: <https://www.psmid.org/diagnosis-and-management-of-urinary-tract-infections-in-adults-2015-update-part-2/>.

178. PIOA. PACIFIC ISLANDS ORTHOPAEDIC ASSOCIATION (PIOA) ANTIBIOTIC USE GUIDELINES. 2017; Available from: <https://www.pioa.net/wp-content/uploads/2019/08/PIOA-Antibiotic-guidelines.pdf>.

179. Health, M.o. Tuvalu standard treatment Guidelines 2010 [cited 1st edition June 22, 2024]; Available from: <https://www.scribd.com/document/480482038/Tuvalu-standard-treatment-guidelines>.

180. UNICEF. STANDARD TREATMENT MANUAL FOR CHILDREN," 2017. 2017 July 26, 2024]; Available from: <https://www.unicef.org/pacificislands/media/851/file/Standard-Treatment-Manual.pdf>.

181. Health, M.o. Solomon Islands guidelines for the management of major non-communicable diseases (NCDs) in primary health care 2011; Available from: <https://aaopenplatform.accessaccelerated.org/resource-library/content/solomon-islands-guidelines-management-major-non-communicable-diseases-ncds-primary-health>.
